# Mining Chromatographic Enantioseparation Data Using Matched Molecular Pair Analysis

**DOI:** 10.3390/molecules21101297

**Published:** 2016-09-29

**Authors:** Robert P. Sheridan, Patrick Piras, Edward C. Sherer, Christian Roussel, William H. Pirkle, Christopher J. Welch

**Affiliations:** 1 Department of Structural Chemistry, Merck Research Laboratories, Rahway, NJ 07065, USA; edward_sherer@merck.com; 2 Aix Marseille Université, Centrale Marseille, CNRS, iSm2, 13397 Marseille CEDEX 20, France; christian.roussel@univ-amu.fr; 3 Department of Chemistry, University of Illinois, Urbana, IL 61801, USA; whpirkle@gmail.com; 4 Department of Process Research & Development, Merck Research Laboratories, Rahway, NJ 07055, USA

**Keywords:** matched molecular pairs, chiral chromatography, chiral recognition

## Abstract

We apply matched molecular pair (MMP) analysis to data from ChirBase, which contains literature reports of chromatographic enantioseparations. For the 19 chiral stationary phases we examined, we were able to identify 289 sets of pairs where there is a statistically significant and consistent difference in enantioseparation due to a small chemical change. In many cases these changes highlight enantioselectivity differences between pairs or small families of closely related molecules that have for many years been used to probe the mechanisms of chromatographic chiral recognition; for example, the comparison of N-H vs. N-Me analytes to determine the criticality of an N-H hydrogen bond in chiral molecular recognition. In other cases, statistically significant MMPs surfaced by the analysis are less familiar or somewhat puzzling, sparking a need to generate and test hypotheses to more fully understand. Consequently, mining of appropriate datasets using MMP analysis provides an important new approach for studying and understanding the process of chromatographic enantioseparation.

## 1. Introduction

Finding which type of chiral stationary phase (CSP) will provide the best chromatographic separation for the enantiomers of a given molecule is mostly a trial-and-error process, and since the number of commercial CSPs is now very large, testing a representative sample of columns becomes difficult and time-consuming, even with automated screening of columns. It would be useful to predict in advance which CSP to choose for a particular molecule. There are longstanding rules of thumb to aid in the prediction of enantioseparability for specific CSPs [1,2,3,4,5,6,7], and several attempts have been made over the years to create more broadly applicable statistical models of enantioseparation based on literature data [8,9,10,11]. Recently, we attempted to generate descriptor-based QSAR (quantitative structure-activity relationship) enantioselectivity models [12] for a subset of 19 CSPs based on literature data compiled in ChirBase, a database containing more than 200,000 entries of literature-reported chromatographic enantioseparations [13]. This attempt was a partial success, leading to the generation of self-consistent QSAR models for four of the 19 CSPs, and allowing for reasonably accurate predictions of enantioseparability for in-house racemic compounds from the Merck sample repository that were not included in the training sets of the models.

The limited ability to find trends in more CSPs using descriptor-based QSAR methods was somewhat disappointing, but conventional QSAR is not the only way to find activity trends. In this paper we discuss the use of matched molecular pair (MMP) methods [14] for investigating enantioseparation data. MMP techniques find local chemical changes in similar compounds and look for trends where the same type of chemical change is associated with a consistent change in activity. Trends identified by MMP are complementary to those found by conventional QSAR, mostly because conventional QSAR is looking for features that correlate with absolute low or high activity, whereas MMP is looking for activity differences between molecules that can be anywhere in the activity range. There are two major types of algorithms for identifying matched pairs: ones based on maximum common substructure and ones based on the change of predefined fragments. These types of algorithms can identify different pairs.

In this paper we apply MMP analysis to the enantioseparation data from our previous study, and find that, using this approach, many more statistically significant trends are found among the 19 CSPs than were obtained using conventional QSAR. We can see many small changes in structure that lead to very large differences in enantioseparation on specific CSPs. While the causes of these effects are not completely understood, we hope that providing this data may induce others to study this problem and experimental approach in further detail. To our knowledge, this is the first attempt to apply MMP methods to chromatographic enantioseparation data.

## 2. Results and Discussion

Definitions of alpha, Zmax, etc. are given in the Experimental Section. The number of clusters, the expected Zmax, and the number of statistically significant clusters is shown in Table 1. Interestingly, all four CSP_NOs that were statistically significant in cross-validation in our previous QSAR study have at least one statistically significant cluster, but 10 CSP_NOs for which we could not find any trend in our previous study have at least one statistically significant cluster. Especially interesting is CSP_NO 23735 (Chiralpak-AD), which afforded no statistically significant QSAR model in our previous study, but which has 65 statistically significant MMP clusters. The full list of Z, meanDiff, StdDiff, plus citations and SMILES for individual compounds in significant clusters, regardless of restrictions about citations, are provided in Supporting Information.

**Figure 1:**
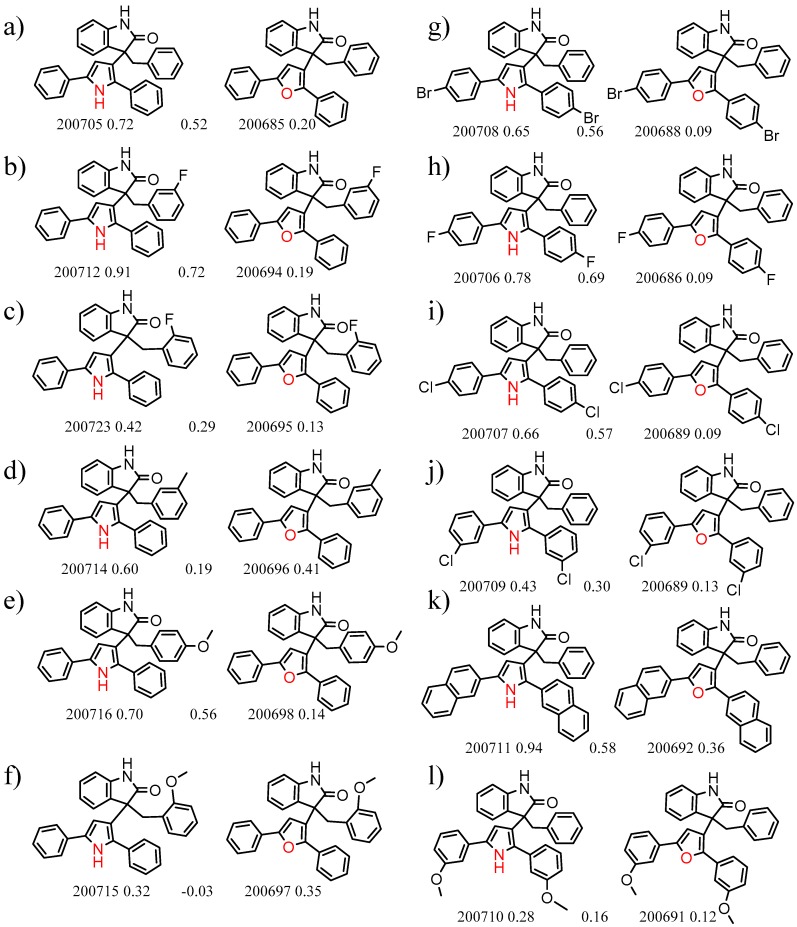
Expanded view of Cluster 1 for CSP_NO 23735 (Chiralpak-AD). There are 12 pairs (a–l) in this cluster. Differences in the structure are highlighted in red. Next to each compound is shown the Chirbase compound number and log(α). The meanDiff within each pair is written between the molecules. This is an example of a cluster where all the values of α come from the same reference.

An example cluster is shown in Figure 1 (Cluster 1 from CSP_NO 23735). In this example, activity data for all 12 pairs was taken from a single publication by Yuan and coworkers [15]. Interestingly, this publication was focused on the synthesis of these compounds, and did not comment on the somewhat remarkable differences in chromatographic enantioselectivity.

**Figure 2:**
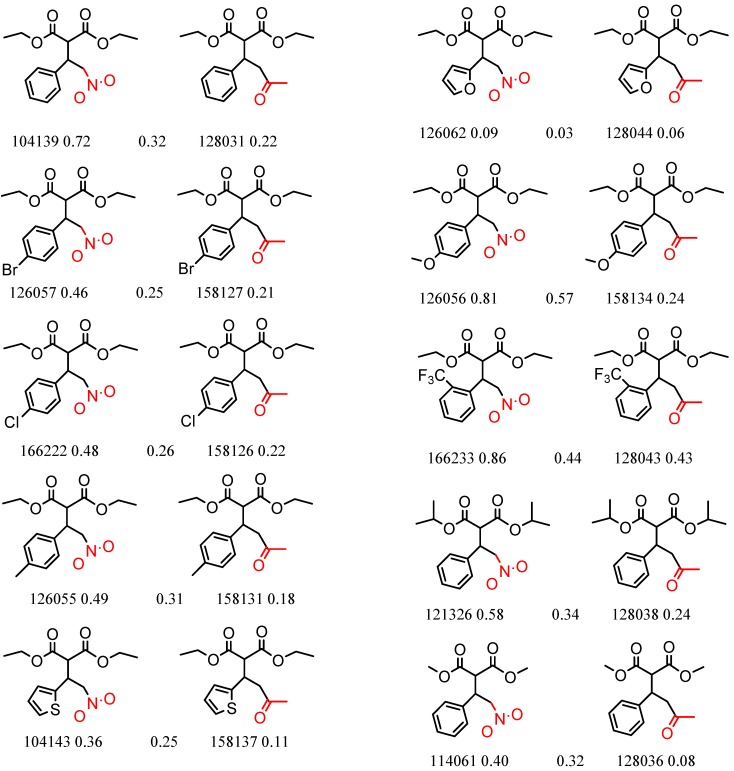
Expanded view of Cluster 2 for CSP_NO 23735 (Chiralpak-AD). There are 10 pairs in this cluster. Differences in structure are highlighted in red. Next to each compound is shown the Chirbase compound number and log(α). The meanDiff within each pair is written between the molecules. This is an example of a cluster were the values of α can come from different references. See supporting information for conformational modeling showing differences in preferred conformations of nitro and methyl ketone analogs.

Figure 2 shows Cluster 2 from CSP_NO 23735, a highly significant cluster with 10 pairs coming from seven different citations. Increased enantioselectivity for nitro vs. methyl ketone is observed across the entire series.

Table 2 lists a summary of statistically significant clusters where within each A-B pair the value of α comes from the same citation or the same set of authors, and the cluster contains at least two pairs. The citations are also listed in the table. The chemical change is summarized by the first pair in the cluster. Since the order of pairs in the cluster is arbitrary, the first pair may not have the largest change in enantioseparation. The other pairs have the same change in very similar molecules, as is seen in Figure 1 and Figure 2.

Overall, a general tendency can be seen for MMPs with small chemical changes in structure, such as adding, changing, or moving a single atom, to give rise to large differences in enantioselectivity, up to 0.8–0.90 in log (α), or a factor of 6–8 in α. Clearly, MMPs showing such profound effects of structure on enantioselectivity can be helpful in understanding the mechanisms underlying the chromatographic separation of enantiomers. CSPs showing more unique molecule entries in ChirBase tend to show a greater number of significant clusters, a not unsurprising result given that with more molecules there is a greater chance of seeing something above noise. While a detailed investigation is beyond the scope of this study, several MMPs appearing in Table 2 that have a bearing on established theories for chiral chromatographic recognition will be pointed out. For the sake of brevity, the MMP clusters presented in the following discussion will be represented using a single pair from each cluster, however, the entire data set is available in the Supporting Information.

The chromatographic separation of enantiomers on CSPs relies upon the formation of transient diastereomeric adsorbates of differing free energy, with the more strongly adsorbed enantiomer spending proportionally more time on the column, and consequently eluting after the less strongly adsorbed enantiomer. Enantioseparation relies on a difference in the ability of the two enantiomers to undergo simultaneous bonding (or repulsive) interactions with the CSP. A variety of molecular interactions have been invoked to explain chiral molecular recognition on CSPs, with hydrogen bonding, π-π complexation of aromatic rings, ionic interactions and steric repulsion being the most common. Investigations into the mechanisms of chiral recognition on CSPs often examine the effect of systematic changes in chemical structure on chromatographic retention and enantioselectivity, with comparison between pairs or small families of closely related molecules sometimes being used to probe the mechanism of chromatographic chiral recognition for a given analyte-CSP combination.

For example, in Figure 1, replacement of the pyrrole nitrogen (NH) with a furan oxygen (O) leads to a marked decrease in enantioselectivity on the Chiralpak AD CSP, with a difference of 0.72 log(α) being observed in the most extreme case (α of 1.53 for the furan; α of 8.07 for the pyrrole). While a detailed investigation into the chiral recognition mechanisms for these molecules on this CSPs is beyond the scope of the present study, the extreme difference observed in this MMP cluster could suggest beneficial involvement of a pyrrole N-H hydrogen bond in chiral recognition with the chiral selector on the Chiralpak AD CSP. More subtle effects on chiral recognition for this series can also be observed. For example, *m*-F substitution on the benzyl aromatic group (b) enhances the enantioseparation gap between the pyrrole and furan, whereas *m*-Me (d), *o*-F or *o*-OMe substitution (structures d, c and f) leads to a sharp decrease the enantioseparation gap. Substitution in the *para* position of the aryl groups flanking the pyrrole or furan is seen to increase the enantioselectivity gap (g, h, I, k) while *meta* substitution decreases it (j, l).

**Figure 3:**
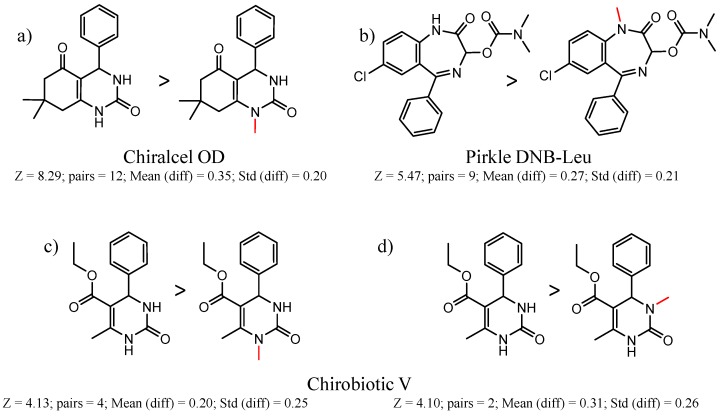
Matched Molecular Pairs illustrating the effects of N-H methylation on several analyte-CSP combinations.

Hypotheses about the involvement of an N-H hydrogen bond in chiral recognition can be also be tested by comparing enantioselectivity with the corresponding N-Me analog [61]. Four MMP clusters where NH methylation leads to a significant decrease in α are shown in Figure 3, suggesting an important beneficial role of the targeted N-H in chiral recognition in each of these cases.

**Figure 4:**
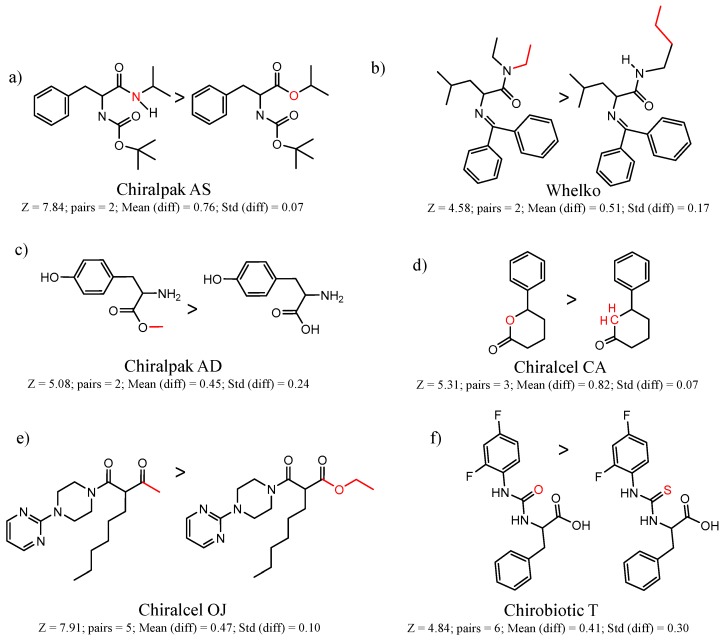
Matched Molecular Pairs illustrating effects of hydrogen bonding on chiral recognition.

Similarly, Figure 4 shows several MMP clusters that provide insight into the relative importance of hydrogen bonding of particular groups in chiral recognition on different CSPs. In Figure 4a, replacement of an ester O with an amide N-H leads to a strong increase in enantioselectivity, again suggesting involvement of the NH in chiral recognition. On the other hand, 4b and 4c show cases where the more strongly hydrogen bonding substituent leads to decreased enantioselectivity, a result that can arise from specifically increased adsorption of the less retained enantiomer or through generalized non-specific adsorption [62].

Figure 4d shows an interesting case suggesting the importance of a lactone oxygen in chiral recognition while Figure 4e compares keto amide clusters to analogs where the ketone is replaced with an ester, leading to a sharp decrease in enantioselectivity. Conformational modeling (SI) reveals distinctly different conformations of the two analytes, with intramolecular CH-hydrogen bonding of the methyl ketone leading to a preferred conformation with *anti*-disposed carbonyl oxygens and the ester analog showing considerable conformational flexibility and a preference for *syn* oriented carbonyls. Figure 4f compares clusters containing ureas and thioureas, showing much higher enantioselectivity in the former case. Again, the results are somewhat puzzling, but may be attributable to differences revealed by conformational modeling, with the thiourea showing a strong preference for an intramolecularly hydrogen bonded conformation where the aryl rings are also participating in π-π stacking (SI).

**Figure 5:**
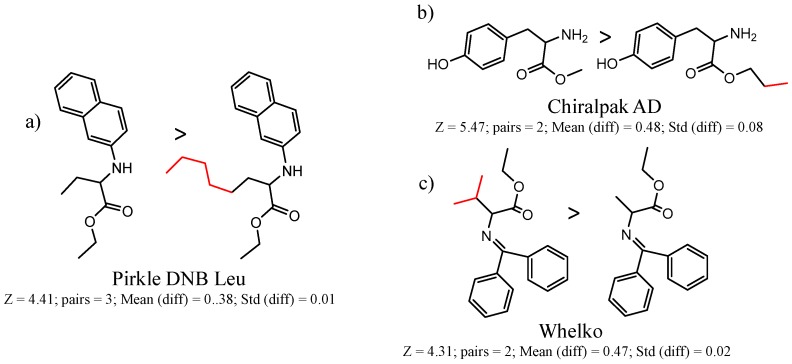
Matched Molecular Pairs illustrating effects of chain length and steric bulk on chiral recognition.

Figure 5 shows several MMP clusters in which change in the length or bulk of an alkyl substituent leads to a large difference in enantioselectivity. Examples where shorter chain lengths lead to improved enantioselectivity are shown in Figure 5a,b, although many cases where the opposite trend is observed are also known. The classic studies of Pirkle and co-workers [63,64] provide detailed illustrations where, depending on orientation, increase in chain length can lead to dramatic improvement or decrease in enantioselectivity, or to situations where little change is observed. Figure 5c shows a cluster where change of a methyl to an isopropyl leads to enhanced enantioselectivity, a frequently observed trend toward improved enantioseparation with increased steric bulk at the stereocenter [1].

**Figure 6:**
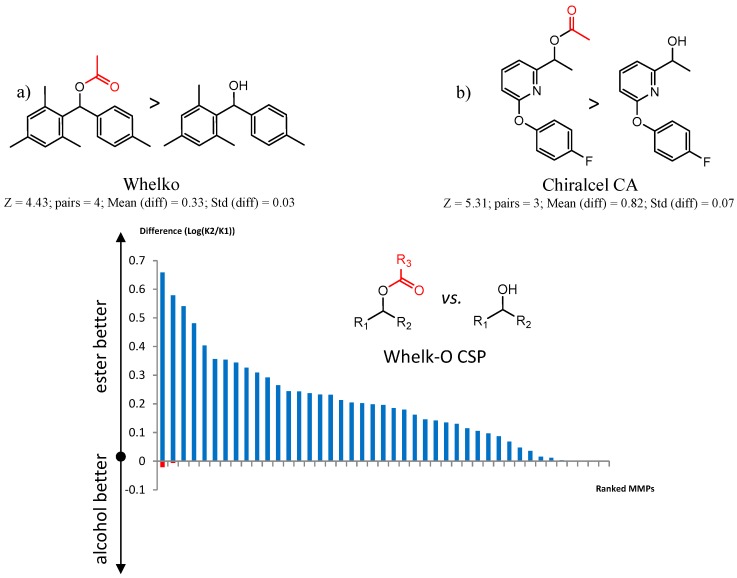
Matched Molecular Pairs illustrating effects of ester vs. alcohol on chiral recognition. (**a**) MMP from Whelk-O CSP (**b**) MMP from Chiralcel CA CSP (**c**) Distribution of MMPs illustrating effects of ester vs. alcohol on chiral recognition on Whelk-O CSPs. MMPs are ranked according to difference in log(α).

Figure 6 illustrates two MMP clusters showing improvement in separation of the enantiomers of carbinols as their acetyl derivatives. This general trend has been observed and commented on for a variety of acyl groups with the Whelko CSPs [65], where the improvement in enantioselectivity is thought to be due to a mechanism-based advantage in the placement of a hydrogen bonding acceptor out of plane from the aromatic group. An analysis of MMPs for alcohol vs. the corresponding ester on the Whelko CSPs shows 39 instances of improved enantioselectivity for the acyl derivatives, with only two cases where the parent alcohol showed no improvement of enantioselectivity (Figure 6c). The same trend is observed for Chiralcel CA-1 with only three cases where the parent alcohol showed improved enantioselectivity. However, this trend is not universal for all the 19 CSPs.

**Figure 7:**
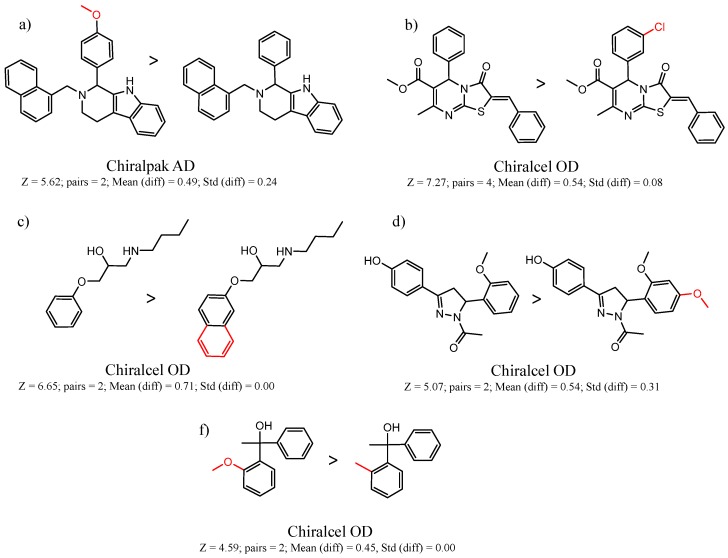
Matched Molecular Pairs illustrating effects of aromatic substituents on chiral recognition.

Figure 7 shows a number of MMPs in which changes in aromatic substituents lead to large differences in enantioselectivity. Investigating aromatic substituent effects has long been used as a means to better understand chiral molecular recognition, particularly for the π-acidic and π-basic CSPs where π-π interaction is believed to be important. Hammet-type analysis has often been used to gain an appreciation of the relative contribution of these electronic effects, although the results are often only roughly directional in nature [3,66,67,68].

**Figure 8:**
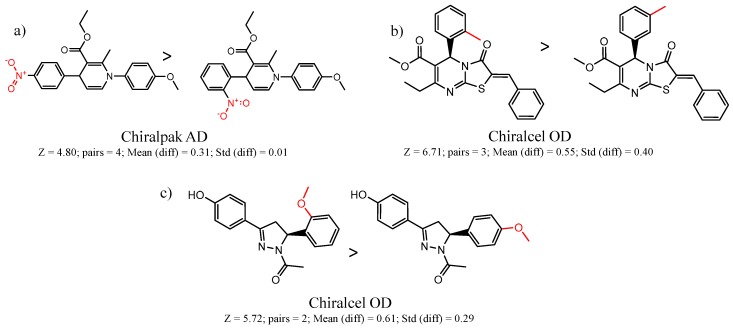
Matched Molecular Pairs illustrating the effects of *ortho* substitution on chiral recognition.

Figure 8 shows several MMP clusters where the effects of *ortho* aromatic substitution on chiral recognition can be observed. *Ortho* substitution of aromatic rings often leads to unusual and unexpected results in chromatographic enantioseparation, when compared with substitution at the *para* or *meta* positions [61,63]. This behavior is thought to be caused by disruption of molecular conformation caused by steric effects of the *ortho* substituent. In Cluster a, *ortho* substitution dramatically decreases enantioselectivity, whereas in clusters b and c, *ortho* substitution improves enantioselectivity.

**Figure 9:**
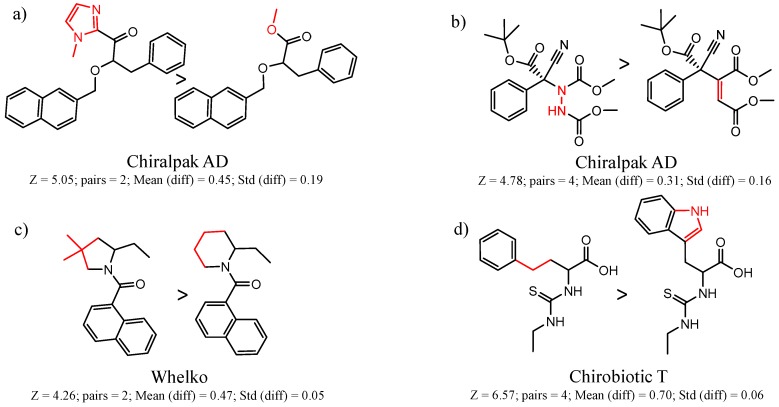
Matched Molecular Pairs illustrating difficult to categorize effects that would normally escape notice.

While some MMPs turned up in the study help to corroborate previous notions about chromatographic enantioseparation, others are unusual, strange, or in some way difficult to interpret. Surfacing of these new relationships may be one of the most valuable aspects of the MMP data mining approach. Several examples of puzzling MMP clusters are presented in Figure 9. For example, the hydrazine and the olefin shown in 9b are markedly different in terms of structure, electronic properties, ability to participate in hydrogen bonding and bond rotatability, but the very fact that the MMP search brings this pair to our attention forces us to consider certain aspects of chiral recognition that would otherwise escape our notice.

It is important to point out that caution must be exercised when interpreting differences between MMPs, as a simple atom or group differences can often lead to multiple effects. For example, change of an amide NH to N-Me not only eliminates a hydrogen bond donor site, but also leads to increased electron density on the amide carbonyl oxygen and often to a shifting in the population of amide rotors. Similarly, introduction of a methoxy substituent on an aromatic ring leads to increased π-basicity, but also introduces a group that can participate in hydrogen bonding or steric interactions. Finally, in this study we have discussed MMP changes in terms of particular analyte-CSP interactions, but in some cases, more general effects could be at play. For example, some MMPs may reflect a general tendency for improved enantioselectivity on any CSP, simply reflecting a molecular change that makes a subtly chiral molecule more easily discriminated by a variety of different CSPs. An extreme example would be the MMP comparing a compound containing a ‘chiral methylene’ (H and D substituents on the stereocenter—almost impossible to resolve chromatographically) with another molecule where D is replaced with almost anything (N, O, Me, etc. generally easily resolved chromatographically).

## 3. Experimental Section

### 3.1. Source of Data

Beginning in the late 1980s, Roussel and co-workers began to develop ChirBase, a database of chiral HPLC separations. The database, which now comprises more than 200,000 entries for over 1800 CSPs, is widely used as a tool for analytical and preparative chromatographic method development. The data included in ChirBase are extracted from the printed and online scientific literature. Each ChirBase record contains different categories of information related to sample, CSP, bibliographic reference, experimental conditions, retention and separation data. Since ChirBase can report only what is reported in the literature, some information may be missing for any given separation record.

### 3.2. Definition of Enantioselectivity

Several metrics for distinguishing the best enantioseparation entry are possible, with chromatographic resolution (Rs) being the most practical term that describes how well two peaks are resolved. However, resolution values are only rarely reported in non-chromatographic literature. Alternatively, the chromatographic separation factor (α) is a useful proxy value that can be a good indicator of the quality of separation [69], and this is the measure selected for this study. The term α is equal to the ratio of the retention factors for the two enantiomers on the CSP (k_2_/k_1_) and is also given by ((t_2_ − t_0_)/t_0_)/((t_1_ − t_0_)/t_0_) where t_1_ and t_2_ are the retention times for the first and second eluted enantiomers, and where t_0_ is the retention time for an unretained solute (the so called ‘void time’ [70]). While many Chirbase entries fail to report α, many entries report retention times for the two enantiomers, column dimensions, and mobile phase flow rate, from which an estimate of t_0_ can be made, thereby allowing an estimate of α [12]. Of course, α is to some degree dependent upon temperature, with some enantioseparations being improved at sub-ambient temperatures and others being improved at elevated temperatures. These changes in enantioselectivity are generally small in the range within which chromatographic experiments are typically carried out. While most of the separations in Chirbase are carried out near room temperature, not all reported separations in the database contain an explicit description of temperature, and for the purposes of these studies, the effect of temperature is considered as a second order perturbation, and not explicitly factored into the modeling approach.

### 3.3. Selection of Data to Model

In our previous work we needed to filter the separation records in Chirbase. Data used in that earlier study and this one required that there be a clearly defined structure for the molecule being separated, that there be a single stereogenic center, and that the reported value of α be within a reasonable range (1–12).

In ChirBase at least some data is missing for most determinations of α, and the only data guaranteed to be present is the structure of the molecule being separated and the stationary phase, represented as the CSP_NO, which is an index specific to ChirBase. ChirBase may represent two stationary phases of opposite chirality, e.g., CrownPak-CR(+) and CrownPak-CR(−), as a single CSP_NO since both should separate enantiomers of a given racemic molecule in an identical manner. Our approach was to build a model predicting α for individual CSP_NO. For that, one must generate a set of unique molecules tested on that CSP_NO and their values of α. Chirbase contains a number of determinations for each unique molecule on a CSP_NO under a variety of “conditions” (temperature, mobile phase, etc.), but as noted above, only some conditions are noted for each record, and even if all the conditions were recorded, there would not be enough data to make a model for each condition. Therefore for our QSAR study we needed to model each unique molecule under “the best recorded condition” for that CSP_NO. The α we use as an activity for a molecule is the 95^th^ percentile value of α over all occurrences of that molecule on the specific CSP_NO. In that way the activity represents the “best recorded condition” for that molecule and CSP_NO while eliminating extreme outliers.

There are ~1800 unique CSP_NOs in the ChirBase, and it would not make sense to model all of them. For most CSP_NOs, not enough unique molecules have been tested to make a generalizable QSAR model. Also, for most CSP_NOs, there is not enough variation in α to make modeling possible, i.e., if there are no examples of both poorly-separated and well-separated molecules, there is no possibility of modeling the ease of separation. Therefore, in our previous paper we focused on 19 CSP_NOs that had ≥100 unique molecules and a standard deviation in α ≥ 1.5. In this study we look at the same CSP_NOs as in the previous study, so a comparison between conventional QSAR and MMP analysis can be made.

### 3.4. Transformation of Data

In QSAR, one may transform the activity (the property being measured or estimated) to obtain a more self-consistent model. In our previous paper we tried α and log(α), the base-10 log. The cross-validated R^2^ for log(α) was almost always better than α. In retrospect this is not surprising since α is a ratio and empirically, QSAR models using the log of the ratio instead of the ratio itself generally perform better. We will use log(α) as the activity in our MMP analysis. For example log(α) = 0 represents an α value of 1, i.e., no enantioseparation, and log(α) = 1 represents an α value of 10, a very high degree of separation. Differences in activity will also be in units of log(α).

### 3.5. Method for Matched Molecular Pairs

We will use the T-ANALYZE [71] method developed in our laboratory. It uses a maximum common substructure method to find the matched pairs. That approach is useful for finding changes within rings, of which we have several examples. Imagine a dataset of molecules with an activity assigned to each. Steps are the following:

1. Pairs of molecules A and B are selected such that A and B are topologically similar (similarity > 0.65 using the atom-pair descriptor and the Dice similarity index). This step is useful because finding maximum common substructure (MCS) is slow and the selection reduces the number of pairs that must be examined by MCS.
2. A MCS is defined for each pair A-B. The change in structure A → B is defined by the RECS (remainder after elimination of common substructure), which is the set of atoms that remain in A and B after the atoms in the MCS are eliminated. One can think of A → B as the “change in structure”. Some A → B changes may be discarded if the changes occur in more than one place on the molecule.
3. Sets of A → B pairs are clustered such that all pairs in each cluster have the same type of chemical change, all the pairs are distinct, and all the molecules in the cluster are topologically similar. That is, each pair in the cluster contains the same change in the same molecular context. Here we will use Npair to be the number of pairs in a cluster. Some clusters may contain a single pair. Nclusters will represent the number of clusters from a particular dataset.
  So far, the analysis has ignored activity. Further steps take the activity into account.
4. Each molecule in the cluster is assigned an activity. Consider a cluster of three pairs A1 → B1, A2 → B2, A3 → B3. One can calculate the difference in activity between A1 and B1, A2 and B2, etc. The mean difference in activity for the cluster is called meanDiff and the standard deviation is called StdDiff. If the absolute value of meanDiff is large and StdDiff is small, the same chemical change is producing an equivalent change in activity. A metric Z compares meanDiff of the cluster with Npair pairs with the meanDiff expected by assembling Npair randomly selected pairs not necessarily in the same cluster:
Z = (meanDiff(real) − meanDiff(random))/StdDiff(random,Npairs)(1)
  For randomly selected pairs, the changes in activity will tend to cancel out, so the expected meanDiff(random) is close to zero and StdDiff will have a value that depends on Npairs. As a consequence, as Npairs gets larger, the StdDiff(random,Npairs) becomes smaller.
  We use Z is to score clusters in a dataset by statistical significance. A cluster with a smaller absolute meanDiff can have a higher absolute Z than a cluster with a larger absolute meanDiff if there are more pairs in the first cluster.
5. By convention, we want the first molecule in the pair to have the higher activity, so we may reassign molecules in all pairs in the cluster as A and B so that meanDiff and Z is positive.
6. Clusters can be written in order of decreasing Z, decreasing meanDiff, or decreasing Npairs. The default of T-ANALYZE is to sort by decreasing Z.

### 3.6. Selection of Statistically Significant Clusters

The term, Z, provides a measure of the statistical significance of a single cluster compared to a cluster composed of randomly selected pairs. This term helps to identify the truly important clusters with a high level of statistical significance and ignore those clusters that may reflect nothing more than chance or statistical fluctuation. One aspect that was not considered in the original formulation of T-ANALYZE is what value of Z one would consider statistically significant given that a very large number of clusters can be produced from a given dataset. If Nclusters is large, it is possible for a high Z to occur by chance even if there is no real consistency between chemical changes and activity changes in the dataset.

**Figure 10:**
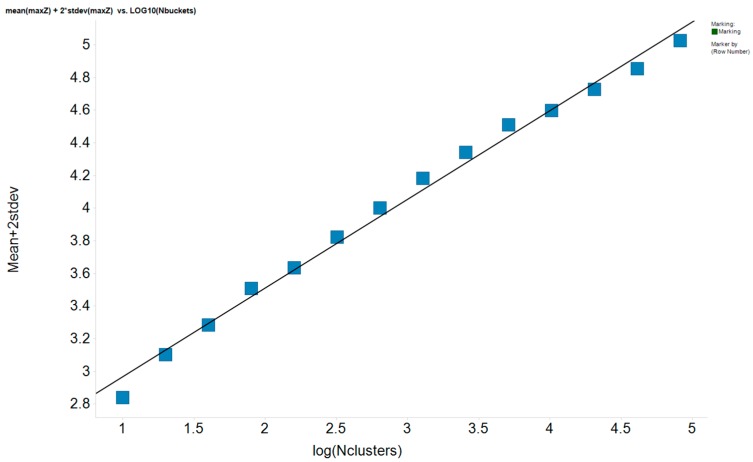
Relationship between the statistically significant level of maxZ and the number of clusters.

We approach this issue by simulating assembling a set of Nclusters clusters. Given that any Z is already normalized by Npairs, we need no longer pay attention to the number of pairs in each cluster. To simulate the Z of a cluster, pick a random number from a Gaussian distribution with a standard deviation of 1 and mean of 0 and take its absolute value. (The absolute value is taken since all Z > 0 by convention in step 5 above). This value for Z represents one simulated cluster. For arbitrary values of Ncluster, generate Ncluster simulated clusters and note the maximum simulated Z over all the clusters, which we will call maxZ. Do this, say, 1000 times. One can calculate a mean and standard deviation for maxZ over these 1000 trials. Only five percent of the time we would expect maxZ to be above mean(maxZ) + 2 standard deviations(maxZ), so any real Z in a group of Nclusters clusters that is above this value is “real” with 95% confidence. That is, in only one out of 20 times will that dataset produce that large a Zmax by chance. We can repeat the above analysis for many values of Nclusters. Figure 10 shows maxZ+2 standard deviations as a function of log(Nclusters). Clearly there is a linear relationship such that we can predict maxZ for any value of Nclusters:

Expected maxZ (95% percentile) = 2.42 + 0.543 log(Nclusters).
(2)

For a dataset with Nclusters clusters, we need consider only those clusters where Z of the cluster exceeds the value in the above equation, as these will be “statistically significant.”

### 3.7. Selection of Clusters

There may be criteria other than statistical significance that we may wish to consider to determine which clusters are interesting. Given that determinations of compounds in a given cluster may be from different labs with the enantioseparation run under different conditions, it is possible that the difference in α between A and B can reflect differences between labs, rather than real differences between molecules. Therefore we might suggest that the values of α in the entire cluster need to be taken from the same citation, or at least from citations with a common set of authors (presumably with the experiment run in the same lab). A weaker version of this might be to require only that the α values of A and B within each pair be from the same authors, the assumption being that what matters is the difference between A and B within each pair, not the difference between pairs.

Another possible criterion for considering a cluster interesting is that it contain at least two pairs. Clusters containing one pair may be statistically significant, but it is better to have confirmation that a particular chemical change produced a change in enantioseparation in the same direction.

## 4. Conclusions

To our knowledge, this is the first time that MMP analysis has been carried out to study chromatographic enantioseparation. We have only begun to investigate this topic, and there are no doubt many more interesting clusters in ChirBase. Here, we confined our analysis to 19 CSP_NOs that were thought to have the best chance of showing activity trends by descriptor-based QSAR methods. However, these results strongly suggest that many other CSP_NOs might have statistically significant clusters by MMP even though they show no trend by QSAR. Also, we did not discuss significant clusters in which the α values for A and B were from citations from different authors. In some of these cases (e.g. in Figure 2) we did see very significant clusters and some of the trends might be real.

Finally it is possible that alternative methods for identifying matched pairs would identify pairs that our maximum common substructure algorithm would not. The fact that small changes in structure gives rise to large changes in enantioseparation in a large variety of CSPs is consistent with the idea that enantiomers make very specific contacts with the CSP. This also might be an explanation as to why making conventional QSAR models of enantioseparation is so difficult, as it is generally accepted that a rugged activity landscape makes finding QSAR difficult.

## Figures and Tables

**Table 1:** Stationary Phase in Chirbase Studied in this Work.

CSP_NO	Description	Unique Molecules	Mean α	Stdev α	Total Clusters	Expected maxZ (95th Percentile)	Number of Significant Clusters
**394**	Chiral-AGP	**574**	**1.65**	**0.90**	**519**	**3.9**	**0**
**2966**	Crownpak-CR(+) ^1^	**346**	**2.03**	**1.52**	**416**	**3.8**	**4**
**3575**	Ultron-ES-OVM	**190**	**1.55**	**0.59**	**90**	**3.4**	**0**
**15723**	Chirobiotic-R	**462**	**1.93**	**1.51**	**636**	**3.9**	**0**
**23735**	Chiralpak-AD	**11167**	**1.45**	**0.64**	**8348**	**4.6**	**65**
**45167**	Chiralpak-AS	**3665**	**1.48**	**0.68**	**4296**	**4.4**	**30**
**45172**	Chiralpak-IA	**1373**	**1.53**	**0.81**	**2099**	**4.2**	**11**
**45173**	Chiralcel-OD	**14350**	**1.47**	**0.64**	**22112**	**4.8**	**48**
**90201**	Chiralcel-CA-1	**470**	**1.82**	**1.51**	**561**	**3.9**	**2**
**90220**	Chiralcel-OF	**291**	**1.40**	**0.63**	**257**	**3.7**	**4**
**90246**	Chiralcel-OJ	**4256**	**1.44**	**0.63**	**5108**	**4.4**	**20**
**90292**	(DNB)-Leu ^1^	**610**	**1.71**	**1.17**	**1296**	**4.1**	**5**
**90589**	Whelk-O ^1^	**1754**	**1.59**	**1.01**	**2246**	**4.2**	**14**
**90613**	Chiralcel-OZ	**314**	**1.53**	**0.76**	**204**	**3.6**	**0**
**90704**	Chirobiotic-V	**351**	**1.37**	**0.73**	**411**	**3.8**	**16**
**90752**	Chiralpak-AY	**216**	**1.67**	**0.99**	**108**	**3.5**	**0**
**90879**	Chirobiotic-T ^1^	**1154**	**2.20**	**1.96**	**1920**	**4.2**	**20**
**91119**	Chirobiotic-TAG	**308**	**1.87**	**1.59**	**329**	**3.8**	**7**
**91423**	Chiralpak-IB	**680**	**1.39**	**0.62**	**876**	**4.0**	**11**

^1^ Statistically significant by descriptor-based QSAR.

**Table 2:** Clusters where M > 1 and the activities for A and B come from the same reference.

CSP_NO	Cluster	Z	No. Pairs	Mean (Diff)	Std (Diff)	Example	Reference
23735 Chiralpak-AD	1	11.52	12	0.43	0.23	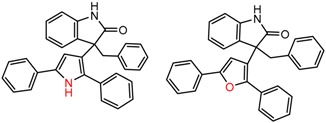	[15]
	5	6.93	6	0.35	0.03	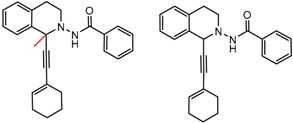	[16]
	27	5.62	2	0.49	0.24	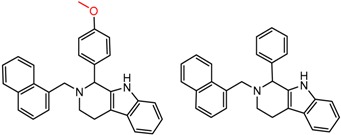	[17]
	28	5.61	2	0.49	0.27	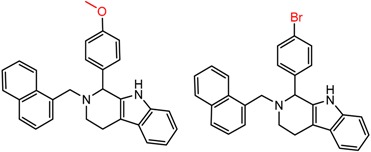	[17]
	35	5.47	2	0.48	0.08	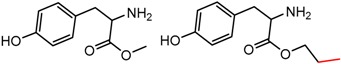	[18,19]
	44	5.14	2	0.46	0.07	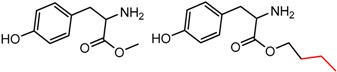	[18,19]
	45	5.10	5	0.29	0.24	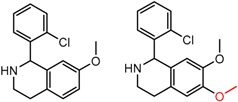	[20]
	46	5.08	2	0.45	0.24	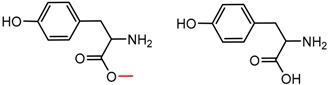	[19,21]
	47	5.05	2	0.45	0.19	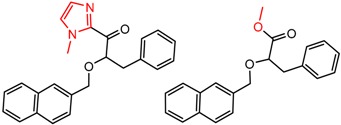	[22]
	61	4.80	4	0.31	0.01	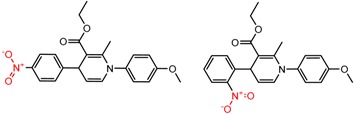	[23]
	65	4.78	4	0.31	0.16	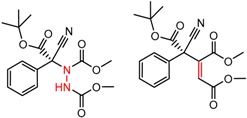	[24,25]
45167 Chiralpak-AS	1	7.84	2	0.76	0.07	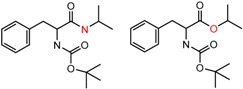	[26,27,28]
	28	4.57	2	0.44	0.29	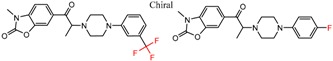	[29]
45173 Chiralcel-OD	1	8.36	2	0.90	0.01	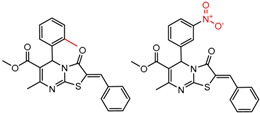	[30]
	2	8.29	12	0.35	0.20	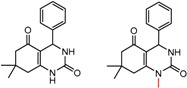	[31]
	3	8.05	2	0.86	0.08	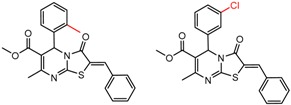	[30]
	5	7.27	4	0.54	0.08	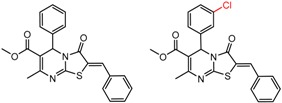	[30]
	6	7.00	4	0.52	0.09	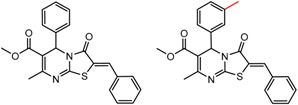	[30]
	7	6.86	4	0.51	0.05	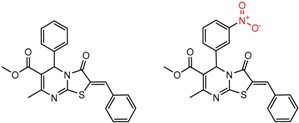	[30]
	9	6.71	3	0.55	0.40	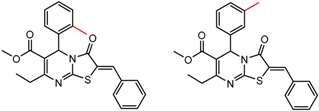	[30,32]
	11	6.65	2	0.71	0.00	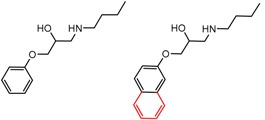	[33]
	15	6.17	3	0.51	0.09	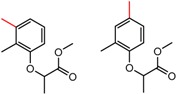	[34,35]
	16	5.86	2	0.62	0.06	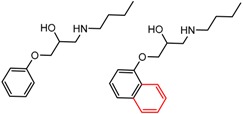	[36,37]
	17	5.85	2	0.62	0.06	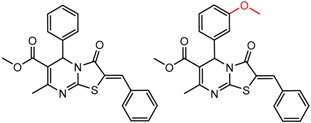	[30]
	18	5.82	2	0.62	0.06	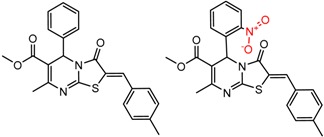	[30]
	19	5.76	3	0.48	0.13	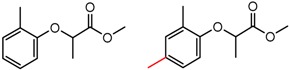	[30,34,35]
	20	5.72	2	0.61	0.29	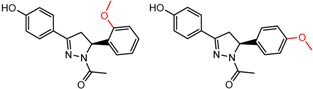	[37]
	23	5.61	2	0.60	0.26	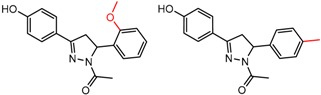	[37]
	25	5.49	2	0.58	0.46	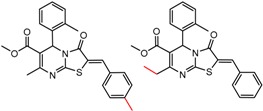	[30,32]
	27	5.43	2	0.57	0.01	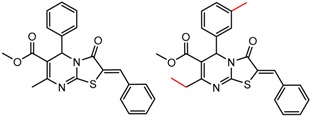	[30,32]
	28	5.43	2	0.58	0.06	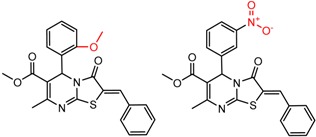	[30]
	33	5.11	2	0.55	0.01	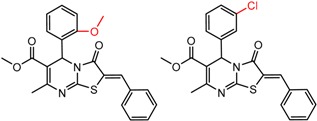	[30]
	35	5.10	2	0.54	0.03	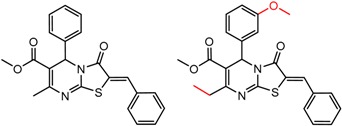	[30,32]
	37	5.07	2	0.54	0.31	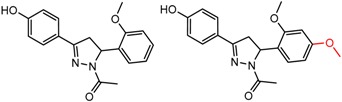	[37]
	44	4.95	3	0.41	0.08	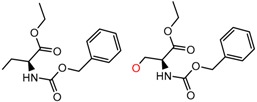	[38,39,40]
	47	4.82	2	0.52	0.01	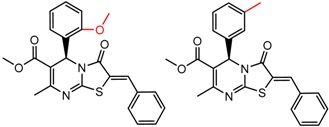	[30]
90201 Chiralcel-CA-1	1	5.31	3	0.82	0.07	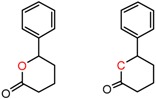	[41,42]
	2	4.25	8	0.42	0.29	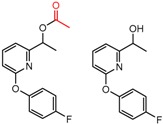	[43]
90246 Chiralcel-OJ	1	7.91	5	0.47	0.10	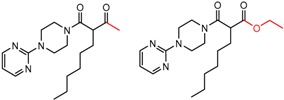	[44,45,46]
	16	4.59	2	0.45	0.00	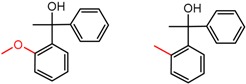	[47,48]
	19	4.45	2	0.43	0.07	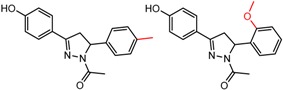	[37]
90292 Pirkle DNB-Leu	1	5.47	9	0.27	0.21	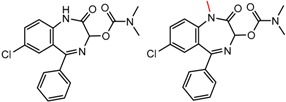	[49,50,51,52]
	4	4.41	3	0.38	0.01	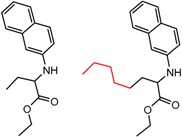	[53]
90589 Whelk-O	10	4.58	2	0.51	0.17	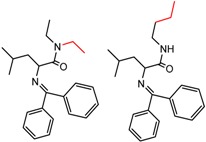	[54]
	12	4.43	4	0.33	0.03	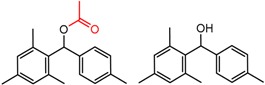	[55]
	13	4.31	2	0.47	0.02	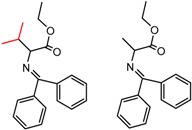	[54]
	14	4.26	2	0.47	0.05	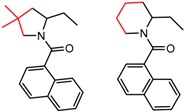	[56,57]
90704 Chirobiotic-V	12	4.52	2	0.34	0.34	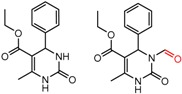	[58]
	13	4.30	2	0.33	0.36	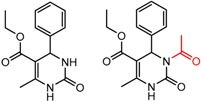	[58]
	15	4.13	4	0.20	0.25	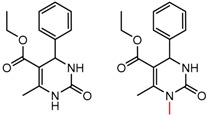	[58]
	16	4.10	2	0.31	0.26	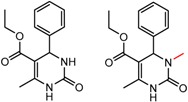	[58]
90879 Chirobiotic-T	1	6.57	4	0.70	0.06	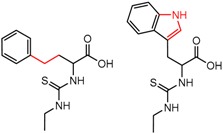	[59]
90879	3	5.29	2	0.85	0.02	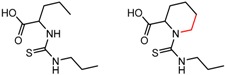	[59]
90879	4	4.84	6	0.41	0.30	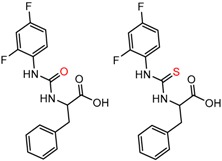	[60]
90879	5	4.76	2	0.77	0.17	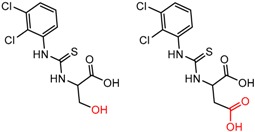	[60]
90879	6	4.73	2	0.76	0.05	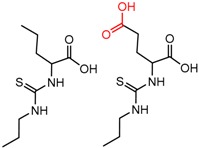	[59]
90879	10	4.62	4	0.49	0.33	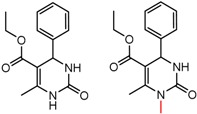	[58]
90879	15	4.48	2	0.72	0.01	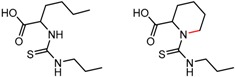	[59]

## Supplementary Materials

Supplementary materials can be accessed at: http://www.mdpi.com/1420-3049/21/10/1297/s1.
