# Supplementary material for: Mining Chromatographic Enantioseparation Data Using Matched Molecular Pair Analysis
Source: Molecules. 2016 Sep 29;21(10):1297. doi: 10.3390/molecules21101297 (PMC6273938; doi:10.3390/molecules21101297)
Supplement: Supplementary file 1 [file molecules-21-01297-s001.pdf]

Supporting Information: Mining Chromatographic Enantioseparation Data using Matched Molecular Pair Analysis

Robert P. Sheridan, Patrick Piras, Edward C. Sherer, Christian Roussel, William H. Pirkle and Christopher J. Welch

1. SMILES for All the Compounds in Set 1

COMPOUND_NAME SMILES
71 O(c1c(OCC=C)cccc1)C[C@H](CNC(C)C)O
73 c12c(OC[C@H](CNC(C)C)O)cccc1cccc2
74 c12c([nH]cc2)cccc1OC[C@H](CNC(C)C)O
105 n1c(Oc2ccc(F)cc2)cccc1[C@@H](O)C
106 n1c(Oc2ccc(Cl)cc2)cccc1[C@@H](O)C
107 n1c(Oc2ccc(Br)cc2)cccc1[C@@H](O)C
108 n1c(Oc2ccc(I)cc2)cccc1[C@@H](O)C
110 n1c(Oc2ccc(cc2)OC)cccc1[C@@H](O)C
111 n1c(Oc2ccc(C#C)cc2)cccc1[C@@H](O)C
112 n1c(Oc2ccc(C=C)cc2)cccc1[C@@H](O)C
113 n1c(Oc2ccccc2)cccc1[C@@H](O)C
114 n1c(Oc2ccccc2)cccc1[C@H](OC(=O)C)C
115 n1c(Oc2ccc(F)cc2)cccc1[C@@H](OC(=O)C)C
116 n1c(Oc2ccc(Cl)cc2)cccc1[C@@H](OC(=O)C)C
117 n1c(Oc2ccc(Br)cc2)cccc1[C@@H](OC(=O)C)C
118 n1c(Oc2ccc(I)cc2)cccc1[C@@H](OC(=O)C)C
120 n1c(Oc2ccc(cc2)OC)cccc1[C@@H](OC(=O)C)C
121 n1c(Oc2ccc(C#C)cc2)cccc1[C@@H](OC(=O)C)C
122 n1c(Oc2ccc(C=C)cc2)cccc1[C@@H](OC(=O)C)C
159 C1[C@@H](c2ccccc2)CCCC1=O
161 C1(OCC[C@@H](C1)c1ccccc1)=O
163 O1[C@@H](c2ccccc2)COCC1
164 O1C[C@H](c2ccccc2)CCC1
165 C1(O[C@H](c2ccccc2)CCO1)=O
1390 N1(C([C@](c2ccccc2)(CC)C(NC1=O)=O)=O)C
1782 C1([C@@H](c2ccccc2)CCCC1)=O
2077 C([C@H](Oc1ccccc1)C)(=O)O
2089 C(N[C@H](C(=O)O)c1ccccc1)(=O)OCc1ccccc1
2091 C(N[C@H](C(=O)O)C(C)C)(=O)OCc1ccccc1
3751 C(O[C@H](c1ccccc1)C)(=O)c1ccccc1
4564 C(N[C@H](C(=O)OCC)C)(=O)OCc1ccccc1
4631 C(N[C@H](C(=O)OCC)CC)(=O)OCc1ccccc1
4632 C(N[C@H](C(OCc1ccccc1)=O)CC)(=O)OCc1ccccc1
4647 C(N[C@H](C(=O)OCC)CO)(=O)OCc1ccccc1

4648 C(N[C@@H](C(OCc1ccccc1)=O)CO)(=O)OCc1ccccc1
4674 C(N[C@H](C(=O)OCC)CC)(=O)OCc1ccccc1
4676 C(N[C@H](C(=O)OCC)C(C)C)(=O)OCc1ccccc1
4678 C(N[C@H](C(=O)OCC)CCC)(=O)OCc1ccccc1
4682 C(N[C@H](C(=O)OCC)CCCC)(=O)OCc1ccccc1
5312 O1[C@](c2c3c(ccc(c3)C)cc(c2)C)(c2c(cccc2)C1=O)C
7444 C([C@@H](c1ccccc1)N)(=O)O
7928 C(c1ccccc1)[C@H](N)C
7979 c1([C@@H](N)CO)ccccc1
8937 N1=C(c2c(ccc(c2)Cl)NC([C@H]1C)=O)c1ccccc1
8940 N1=C(c2c(ccc(c2)Cl)NC([C@H]1C(C)C)=O)c1ccccc1
8944 N1=C(c2c(ccc(c2)Cl)NC(C1Cc1ccccc1)=O)c1ccccc1
8949 N1=C(c2c(ccc(c2)Cl)N(C([C@H]1C)=O)C)c1ccccc1
8950 N1=C(c2c(ccc(c2)Cl)N(C([C@H]1C(C)C)=O)C)c1ccccc1
8964 N1=C(c2c(ccc(c2)Cl)NC([C@H]1OC(=O)c1ccccc1)=O)c1ccccc1
9103 C([C@H](Nc1cc2ccccc2cc1)C)(=O)OCC
9104 C([C@H](Nc1cc2ccccc2cc1)CC)(=O)OCC
9105 C([C@H](Nc1cc2ccccc2cc1)CCC)(=O)OCC
9115 C([C@H](Nc1cc2ccccc2cc1)CCCCC)(=O)OCC
9116 C([C@H](Nc1cc2ccccc2cc1)CCCCCC)(=O)OCC
9117 C([C@H](Nc1cc2ccccc2cc1)CCCCCCC)(=O)OCC
10688 C(#C[C@H](\C=C\c1ccccc1)O)c1ccccc1
11565 [nH]1c2c(c(OC[C@H](CNC(C)C)O)ccc2)c2c1cccc2
12253 C([C@H](Oc1ccc(cc1)Cl)C)(=O)OC
12258 C([C@H](Oc1ccccc1)C)(=O)OC
12355 O1[C@](c2c(c(cc3c2cccc3)C)C)(c2c(cccc2)C1=O)C
12553 C([C@@H](Oc1c(C)cccc1)C)(=O)O
12554 O(c1c(C)cccc1)[C@@H](C(=O)OC)C
12555 O(c1c(C)cccc1)[C@H](C(=O)OCC)C
12558 C([C@@H](Oc1cc(C)ccc1)C)(=O)O
12562 C([C@@H](Oc1ccc(C)cc1)C)(=O)O
12564 O(c1c(Cl)cccc1)[C@@H](C(=O)OC)C
14161 N=1[C@@H](C(N(C)c2c(C1c1ccccc1)cc(cc2)Cl)=O)OC(=O)c1ccccc1
16609 O(c1c(cc(cc1)Cl)Cl)[C@H](C(=O)OC)C
18625 N1=C(c2c(ccc(c2)Cl)NC([C@H]1OC(N(C)C)=O)=O)c1ccccc1
18630 N=1[C@H](C(N(C)c2c(C1c1ccccc1)cc(cc2)Cl)=O)OC(N(C)C)=O
20048 C=1([C@@H](C(C(=O)OC)=C(NC1C)C)c1c(c(Cl)ccc1)Cl)C(=O)OCC
22008 O1C(=O)CCC[C@@H]1c1ccccc1
22016 N(C[C@H](COc1ccccc1)O)C(C)C
22017 C(O[C@H](CNC(C)C)COc1ccccc1)(=O)C
22256 c12c(c(O)ccc1OC[C@H](CNC(C)C)O)cccc2
24136 C(C[C@@H](\C=C\c1ccccc1)O)(=O)c1ccccc1
24647 C(N[C@H](C(=O)OC)CC)(=O)OCc1ccccc1

24648 C(N[C@H](C(=O)OC)CCC)(=O)OCc1ccccc1
24708 C(N[C@H](CC(=O)OC)c1ccccc1)(=O)OCc1ccccc1
24709 C(N[C@H](CC(=O)OC)c1occc1)(=O)OCc1ccccc1
24710 C(N[C@H](CC(=O)OC)c1sccc1)(=O)OCc1ccccc1
25319 N=1[C@H](C(N(C)c2c(C1c1ccccc1)cc(cc2)Cl)=O)OC(=O)NC
25320 N1=C(c2c(ccc(c2)Cl)NC([C@@H]1OC(=O)NC)=O)c1ccccc1
25321 N=1[C@@H](C(N(C)c2c(C1c1ccccc1)cc(cc2)Cl)=O)OC(N(CO)C)=O
25322 N1=C(c2c(ccc(c2)Cl)NC([C@H]1OC(N(CO)C)=O)=O)c1ccccc1
25323 N=1[C@H](C(N(C)c2c(C1c1ccccc1)cc(cc2)Cl)=O)OC(=O)N
25324 N1=C(c2c(ccc(c2)Cl)NC([C@@H]1OC(=O)N)=O)c1ccccc1
25325 N=1[C@H](C(N(C)c2c(C1c1ccccc1)cc(cc2)Cl)=O)OC(NCO)=O
25326 N1=C(c2c(ccc(c2)Cl)NC([C@@H]1OC(NCO)=O)=O)c1ccccc1
25327 N=1[C@H](C(N(C)c2c(C1c1ccccc1)cc(cc2)Cl)=O)OC(N(CO)CO)=O
25328 N1=C(c2c(ccc(c2)Cl)NC([C@@H]1OC(N(CO)CO)=O)=O)c1ccccc1
26214 c1(oc2ccccc2c1)[C@H](n1cncc1)c1ccc(cc1)Cl
26955 c1([S@](Cc2ccccc2)=O)c(C)cccc1
27472 N(C[C@@H](COc1ccc(cc1)O)O)C(C)C
27987 C([C@H](c1ccccc1)O)(=O)N
29317 C([C@H](Oc1ccccc1)CC)(=O)O
31206 c1([C@H](c2ccccc2)O)c(OC)cccc1
31208 c1([C@H](c2ccccc2)O)c(F)cccc1
31209 c1([C@H](c2ccccc2)O)c(C)cccc1
31515 C1(Oc2ccccc2C1)(C(=O)O)CC
31518 O1[C@](C(=O)OC)(Cc2ccccc12)CC
31521 O1[C@](C(=O)N)(Cc2ccccc12)CC
37727 C([C@@H](Cc1cnccc1)N)(=O)O
40459 C([C@H](Oc1ccc(cc1)CC)C)(=O)OC
40461 c1(O[C@@H](C(=O)OC)C)c(c(C)ccc1)C
40462 c1(O[C@@H](C(=O)O)C)c(c(C)ccc1)C
40464 O(c1c(cc(cc1)C)C)[C@@H](C(=O)OC)C
40466 O(c1c(cc(cc1)C)C)[C@@H](C(=O)O)C
40472 C([C@H](Oc1cc(C)c(cc1)C)C)(=O)O
41899 C1(=C(NC(=O)N[C@@H]1c1ccccc1)C)C(=O)OCC
41900 C1(=C(NC(=O)N[C@@H]1c1ccccc1)C)C(=O)OCC
41905 C1(=C(N(C(=O)N[C@@H]1c1ccccc1)C)C)C(=O)OCC
41906 C1(=C(N(C(=O)N[C@@H]1c1ccccc1)C)C)C(=O)OCC
41911 C1(=C(NC(=O)N([C@@H]1c1ccccc1)C)C)C(=O)OCC
41912 C1(=C(NC(=O)N([C@@H]1c1ccccc1)C)C)C(=O)OCC
41917 C1(=C(N(C(=O)N([C@@H]1c1ccccc1)C)C)C)C(=O)OCC
41918 C1(=C(N(C(=O)N([C@@H]1c1ccccc1)C)C)C)C(=O)OCC
41923 N1(C(NC(C)=C([C@H]1c1ccccc1)C(=O)OCC)=O)C=O
41924 N1(C(NC(C)=C([C@H]1c1ccccc1)C(=O)OCC)=O)C=O
41929 N1(C(N(C(C)=C([C@H]1c1ccccc1)C(=O)OCC)C)=O)C=O

41930 N1(C(N(C(C)=C([C@H]1c1ccccc1)C(=O)OCC)C)=O)C=O

41935 N1(C(NC(C)=C(C1c1ccccc1)C(=O)OCC)=O)C(=O)C

41941 C1(=C(NC(=S)N[C@@H]1c1ccccc1)C)C(=O)OCC

41942 C1(=C(NC(=S)N[C@@H]1c1ccccc1)C)C(=O)OCC

41947 C1(=C(N(C(=S)N[C@@H]1c1ccccc1)C)C)C(=O)OCC

41948 C1(=C(N(C(=S)N[C@@H]1c1ccccc1)C)C)C(=O)OCC

41953 N1(C(NC(C)=C([C@H]1c1ccccc1)C(=O)OCC)=S)C(=O)C

41959 C1(=C(NC(=O)N[C@@H]1c1ccccc1)C)C(=O)OC

42013 C1(=C(NC(=O)N[C@@H]1c1ccccc1)C)C(=O)N

42339 C(N[C@H](C(=O)O)C)(=O)c1ccccc1

42341 C(N[C@@H](C(=O)O)CC(C)C)(=O)c1ccccc1

42347 N(C(=O)c1ccccc1)[C@@H](C(=O)O)C(C)C

42444 C([C@H](c1ccccc1)O)(=O)O

43515 C(N1[C@@H](CCC1)C)(c1c2c(cccc2)ccc1)=O

43517 C(N1C[C@@H](C)CC1)(c1c2c(cccc2)ccc1)=O

43521 C(N1[C@H](C)CCCC1)(c1c2c(cccc2)ccc1)=O

43522 C(N1[C@H](CC)CCCC1)(c1c2c(cccc2)ccc1)=O

43524 C(N1C[C@H](C)CCC1)(c1c2c(cccc2)ccc1)=O

44997 n1c[nH]c(c1)C[C@@H](C(=O)O)N

48059 C(N[C@H](C(=O)O)C)(=O)OCc1ccccc1

48071 C(N[C@H](C(=O)O)C(C)C)(=O)OCc1ccccc1

48090 C(N[C@@H](C(=O)O)[C@H](CC)C)(=O)OCc1ccccc1

48095 C(N[C@@H](C(=O)O)[C@H](CC)C)(=O)OCc1ccccc1

48509 C(=O)(c1ccccc1)[C@@H](N)C

48885 C([C@H](c1ccccc1)O)(=O)OC

49043 N1(C([C@@](C2=CCCCC2)(C)C(NC1=O)=O)=O)C

49045 N1(C([C@](c2ccccc2)(CC)C(NC1=O)=O)=O)C

52324 c12c(OC[C@@H](O)CNCCCC)cccc1cccc2

52326 c12c(OC[C@@H](O)CNCCC)cccc1cccc2

57323 C([C@H](C(=O)C)CCC)(N1CCN(c2ncccn2)CC1)=O

57325 C([C@H](C(=O)C)CCCC)(N1CCN(c2ncccn2)CC1)=O

57327 C([C@H](C(=O)C)CCCCC)(N1CCN(c2ncccn2)CC1)=O

57329 C([C@H](C(=O)C)CCCCCC)(N1CCN(c2ncccn2)CC1)=O

57331 C([C@H](C(=O)C)CCCCCCC)(N1CCN(c2ncccn2)CC1)=O

57775 N(=C(/c1ccccc1)c1ccccc1)\[C@H](C(=O)OCC)C

57776 N(=C(/c1ccccc1)c1ccccc1)\[C@H](C(=O)OCC)C(C)C

57787 N(=C(/c1ccccc1)c1ccccc1)\[C@H](C(=O)NCCCC)CC(C)C

57788 N(\[C@H](C(N(CC)CC)=O)CC(C)C)=C(\c1ccccc1)c1ccccc1

57790 N(=C(/c1ccccc1)c1ccccc1)\[C@H](C(=O)NCCCC)C(C)C

57791 N(\[C@H](C(N(CC)CC)=O)C(C)C)=C(/c1ccccc1)c1ccccc1

57792 N(=C(/c1ccccc1)c1ccccc1)\[C@H](C(=O)OC)C

57793 N(=C(/c1ccccc1)c1ccccc1)\[C@H](C(=O)OC)C(C)C

58834 C(=C\c1ccccc1)\[C@H](c1ccccc1)O

58927 C(N[C@H](C(=O)O)Cc1ccccc1)(OC(C)(C)C)=O
58930 C(N[C@H](C(=O)O)C(C)C)(=O)OCc1ccccc1
58931 C(N[C@H](C(=O)O)CC(C)C)(=O)OCc1ccccc1
58933 C(N[C@H](C(=O)O)c1ccccc1)(=O)OCc1ccccc1
59588 C([C@@H](Oc1ccc(CC)cc1)C)(=O)OCC
59590 c1(O[C@H](C(=O)OCC)C)c(c(C)ccc1)C
59591 O(c1c(cc(cc1)C)C)[C@H](C(=O)OCC)C
59602 C([C@@H](Oc1ccc(cc1)Cl)C)(=O)OCC
59618 C([C@@H](Oc1ccccc1)C)(=O)OCC
61170 C(N[C@@H](C(=O)NC)Cc1ccccc1)(OC(C)(C)C)=O
61172 C([C@@H](NC(OC(C)(C)C)=O)Cc1ccccc1)(N(C)C)=O
61174 C(N[C@H](C(=O)NCC)Cc1ccccc1)(OC(C)(C)C)=O
61176 C([C@@H](NC(OC(C)(C)C)=O)Cc1ccccc1)(N(CC)CC)=O
61178 C(N[C@H](C(NC(C)C)=O)Cc1ccccc1)(OC(C)(C)C)=O
61180 C([C@@H](NC(OC(C)(C)C)=O)Cc1ccccc1)(N(C(C)C)C(C)C)=O
64228 N1=C(c2c(cc3c(c2)OCO3)C[C@H](N1)C)c1ccc(N)cc1
64252 N=1N(C(=O)NC)[C@@H](Cc2c(C1c1ccc(N)cc1)cc1c(OCO1)c2)C
66799 C([C@H](Cc1ccccc1)O)(=O)O
67293 C(O[C@H](c1ccccc1)CO)(=O)c1ccccc1
67346 c1([C@@](c2ccccc2)(O)C)ccc(cc1)Br
67546 C(C(=O)OC)[C@@H](c1ccccc1)O
69233 C([C@@H](Cc1cc(O)c(cc1)O)N)(=O)O
69328 c1(c[nH]c2c1cccc2)C[C@@H](C(=O)O)N
70299 c12cc(OC[C@@H](O)CNCCC)ccc1cccc2
70302 c12cc(OC[C@@H](O)CNCCCC)ccc1cccc2
70311 O(C[C@@H](O)CNCCC)c1ccccc1
70314 O(C[C@@H](O)CNCCCC)c1ccccc1
70318 N(C(C)(C)C)C[C@@H](COc1ccccc1)O
71392 C=12[C@H](NC(NC1CCCC2=O)=S)c1c(Cl)cccc1
71395 C=12[C@H](NC(NC1CCCC2=O)=S)c1c(Br)cccc1
71398 C=12[C@H](NC(NC1CCCC2=O)=S)c1c(C)cccc1
71404 C=12[C@H](NC(NC1CCCC2=O)=S)c1c(OCC)cccc1
72053 N(C[C@H](COc1ccc(C=O)cc1)O)C(C)C
72075 O(c1c(CCC)cccc1)C[C@@H](CNC(C)C)O
72081 c1(c(OC[C@@H](CNC(C)C)O)cccc1)C=O
76324 C1([C@@H](Cc2ccccc2)CCCC1)=O
78051 C(C(C)(C)C)(O[C@H](CCc1ccccc1)C)=O
78057 C(C(C)(C)C)(O[C@H](\C=C\c1ccccc1)C)=O
78058 C(O[C@H](\C=C\c1ccc(cc1)C)C)(=O)C
78059 [N+](c1ccc(\C=C\[C@@H](OC(=O)C)C)cc1)([O−])=O
78063 C(N[C@H](C(=O)O)CCC)(=O)OCc1ccccc1
79426 C([C@H](c1ccccc1)O)(=O)O
80260 c1(oc2ccccc2c1)[C@@H](n1cncc1)c1ccccc1

80281 N1(C(OC(C)(C)C)=O)[C@H](C(=O)OC1)Cc1ccccc1
81748 c1(c([S@](Cc2ccccc2)=O)cccc1)C(=O)N
83141 n12c(=NC(CC)=C([C@H]1c1c(C)cccc1)C(=O)OC)sc(\c2=O)=C\c1ccccc1
83144 n12c(=NC(CC)=C([C@H]1c1cc(C)ccc1)C(=O)OC)sc(\c2=O)=C\c1ccccc1
83146 n12c(=NC(CC)=C([C@H]1c1cc(C)ccc1)C(=O)OC)sc(\c2=O)=C\c1ccc(cc1)Cl
83149 n12c(=NC(CC)=C([C@H]1c1cc(OC)ccc1)C(=O)OC)sc(\c2=O)=C\c1ccccc1
83150 n12c(=NC(CC)=C([C@H]1c1cc(OC)ccc1)C(=O)OC)sc(\c2=O)=C\c1ccc(cc1)Cl
83647 C([C@@H](c1ccccc1)N)(=O)O
83711 N1(C([C@](c2ccccc2)(CC)C(NC1=S)=O)=O)C
84685 c1(oc2ccccc2c1)[C@@H](n1nnnc1)c1ccccc1
84689 c1(oc2ccccc2c1)[C@@H](n1nnnc1)c1ccc(cc1)Cl
85512 C([C@@H](Cc1ccccc1)N)(=O)O
85528 C([C@@H](Cc1ccc(O)cc1)N)(=O)O
85608 C([C@@H](Cc1ccc(cc1)F)N)(=O)O
85624 C([C@@H](Cc1ccc(cc1)Cl)N)(=O)O
85640 C([C@H](Cc1ccc(cc1)Br)N)(=O)O
86406 C([C@@](c1ccccc1)(O)C)(=O)O
88157 n12c(=NC(C)=C([C@H]2c2ccccc2)C(=O)OC)sc(\c1=O)=C\c1ccccc1
88158 n12c(=NC(C)=C([C@H]2c2ccccc2)C(=O)OC)sc(\c1=O)=C\c1ccc(C)cc1
88159 n12c(=NC(C)=C([C@H]2c2ccccc2)C(=O)OC)sc(\c1=O)=C\c1ccc(OC)cc1
88160 n12c(=NC(C)=C([C@H]2c2ccccc2)C(=O)OC)sc(\c1=O)=C\c1ccc(cc1)Cl
88162 n12c(=NC(C)=C([C@H]1c1c([N+]([O–])=O)cccc1)C(=O)OC)sc(\c2=O)=C\c1ccc(C)cc1
88164 n12c(=NC(C)=C([C@H]1c1c([N+]([O–])=O)cccc1)C(=O)OC)sc(\c2=O)=C\c1ccc(OC)cc1
88167 n12c(=NC(C)=C([C@H]1c1cc([N+]([O–])=O)ccc1)C(=O)OC)sc(\c2=O)=C\c1ccccc1
88168 n12c(=NC(C)=C([C@H]1c1cc([N+]([O–])=O)ccc1)C(=O)OC)sc(\c2=O)=C\c1ccc(C)cc1
88169 n12c(=NC(C)=C([C@H]1c1cc([N+]([O–])=O)ccc1)C(=O)OC)sc(\c2=O)=C\c1ccc(OC)cc1
88170 n12c(=NC(C)=C([C@H]1c1cc([N+]([O–])=O)ccc1)C(=O)OC)sc(\c2=O)=C\c1ccc(cc1)Cl
88179 n12c(=NC(C)=C([C@H]1c1cc(Cl)ccc1)C(=O)OC)sc(\c2=O)=C\c1ccccc1
88180 n12c(=NC(C)=C([C@H]1c1cc(Cl)ccc1)C(=O)OC)sc(\c2=O)=C\c1ccc(C)cc1
88181 n12c(=NC(C)=C([C@H]1c1cc(Cl)ccc1)C(=O)OC)sc(\c2=O)=C\c1ccc(OC)cc1
88182 n12c(=NC(C)=C([C@H]1c1cc(Cl)ccc1)C(=O)OC)sc(\c2=O)=C\c1ccc(cc1)Cl
88183 n12c(=NC(C)=C(C1c1c(OC)cccc1)C(=O)OC)sc(\c2=O)=C\c1ccccc1
88184 n12c(=NC(C)=C(C1c1c(OC)cccc1)C(=O)OC)sc(\c2=O)=C\c1ccc(C)cc1
88188 n12c(=NC(C)=C([C@H]1c1cc(OC)ccc1)C(=O)OC)sc(\c2=O)=C\c1ccccc1
88192 n12c(=NC(C)=C([C@H]1c1cc(OC)ccc1)C(=O)OC)sc(\c2=O)=C\c1ccc(cc1)Cl
88193 n12c(=NC(C)=C([C@H]1c1c(C)cccc1)C(=O)OC)sc(\c2=O)=C\c1ccccc1
88194 n12c(=NC(C)=C([C@H]1c1c(C)cccc1)C(=O)OC)sc(\c2=O)=C\c1ccc(C)cc1
88197 n12c(=NC(C)=C([C@H]1c1cc(C)ccc1)C(=O)OC)sc(\c2=O)=C\c1ccccc1
88198 n12c(=NC(C)=C([C@H]1c1cc(C)ccc1)C(=O)OC)sc(\c2=O)=C\c1ccc(C)cc1
88199 n12c(=NC(C)=C([C@H]1c1cc(C)ccc1)C(=O)OC)sc(\c2=O)=C\c1ccc(OC)cc1
88200 n12c(=NC(C)=C([C@H]1c1cc(C)ccc1)C(=O)OC)sc(\c2=O)=C\c1ccc(cc1)Cl
88863 C([S@](=O)c1ccccc1)C(=O)O
89680 C1(N(c2ccccc2[C@@]1(c1ccccc1)C)C)=O

90245 C(N[C@H](C(=O)OC)CCC)(=O)OCc1ccccc1
92149 c1(c(cc(cc1)Cl)Cl)[C@H](Cn1cncc1)OCC=C
93217 c12n(\C=C3\C[C@@H]3CO)cnc1c(Cl)nc(n2)N
93218 c12n(\C=C3\C[C@@H]3CO)cnc1c(N)nc(n2)N
93220 c12n(\C=C3\C[C@H]3CO)cnc1c(OC)nc(n2)N
95834 C([C@H](Cc1ccccc1)N)(=O)OC
95836 C([C@H](Cc1ccc(Cl)cc1)N)(=O)OC
95838 C([C@H](Cc1ccc(Cl)cc1)N)(=O)OCC
95840 C([C@@H](Cc1ccc(cc1)O)N)(=O)OC
96670 C=1([C@@H](C(C(=O)OC)=C(NC1C)C)c1c(c(Cl)ccc1)Cl)C(OCOC(=O)CCC)=O
97616 C([S@](=O)c1ccccc1)C(=O)C
97706 C([S@](=O)c1ccccc1)C(=O)OCC
98605 C([C@@H](c1ccccc1)N)(=O)N
98697 C([C@@H](Cc1c(cc(cc1)O)C)N)(=O)O
99891 C(N[C@H](C(=O)O)C)(c1ccc(Br)cc1)=O
99893 C(N[C@H](C(=O)O)C)(c1cc(Cl)ccc1)=O
99894 C(N[C@H](C(=O)O)C)(c1ccc(Cl)cc1)=O
99896 C(N[C@H](C(=O)O)C)(c1ccc(F)cc1)=O
99902 N(C(c1ccc(Br)cc1)=O)[C@@H](C(=O)O)C(C)C
99904 N(C(c1cc(Cl)ccc1)=O)[C@@H](C(=O)O)C(C)C
99919 C(N[C@@H](C(=O)O)CC(C)C)(c1ccc(cc1)Br)=O
100088 C(N[C@H](C(=O)O)CC)(c1cc(Cl)ccc1)=O
101714 C1([C@](c2ccccc2)(COC)CCCC1)=O
104139 C([C@@H](C[N+]([O–])=O)c1ccccc1)(C(=O)OCC)C(=O)OCC
104143 C([C@H](c1sccc1)C[N+]([O–])=O)(C(=O)OCC)C(=O)OCC
104857 c1([C@H](C(=O)O)N)c(C)cccc1
104868 C([C@@H](c1c(C)cccc1)N)(=O)N
105119 c1([C@H](c2ccccc2)O)ccc(OC)cc1
105466 c1([C@H](NC(=O)OCc2ccccc2)C(=O)O)sccc1
105468 c1([C@H](NC(=O)OCc2ccccc2)C(=O)O)occc1
105755 C([C@@H](Cc1ccccc1)N)(=O)OCC
106221 N1(C(N[C@](C1=O)(c1ccccc1)CC)=O)C
106386 [N+](c1ccc(NC([C@H](NC(=O)C)Cc2ccccc2)=O)cc1)([O–])=O
106389 [N+](c1ccc(OC([C@H](NC(=O)C)Cc2ccccc2)=O)cc1)([O–])=O
106392 C([C@H](NC(=O)C)Cc1ccccc1)(Oc1cc2ccccc2cc1)=O
106437 C(N[C@@H](C(=O)OC)CO)(=O)OCc1ccccc1
106615 C([C@@H](Cc1ccc(cc1)O)N)(=O)OCC
106617 C([C@H](Cc1ccc(cc1)O)N)(=O)OCCC
106619 C([C@@H](Cc1ccc(cc1)O)N)(=O)OCCCC
106631 C([C@@H](Cc1ccc(Cl)cc1)N)(=O)OCCC
106633 C([C@@H](Cc1ccc(Cl)cc1)N)(=O)OCCCC
107356 N1(N=C(c2ccc(cc2)O)C[C@H]1c1c(OC)cccc1)C(=O)C
107364 N1(N=C(c2ccc(cc2)O)C[C@H]1c1c(OC)cccc1)C(=O)C

107369 N1(N=C(c2c(cc(cc2)O)O)C[C@H]1c1c(OC)cccc1)C(=O)C
107377 N1(N=C(c2c(cc(cc2)O)O)C[C@H]1c1c(OC)cccc1)C(=O)C
107382 N1(N=C(c2ccc(cc2)O)C[C@H]1c1ccc(OC)cc1)C(=O)C
107390 N1(N=C(c2ccc(cc2)O)C[C@H]1c1ccc(OC)cc1)C(=O)C
107394 N1(N=C(c2c(cc(cc2)O)O)C[C@H]1c1ccc(cc1)OC)C(=O)C
107406 N1(N=C(c2ccc(cc2)O)C[C@H]1c1c(cc(cc1)OC)OC)C(=O)C
107418 N1(N=C(c2c(cc(cc2)O)O)C[C@H]1c1c(cc(cc1)OC)OC)C(=O)C
107426 N1(N=C(c2c(cc(cc2)O)O)C[C@H]1c1c(cc(cc1)OC)OC)C(=O)C
107430 N1(N=C(c2ccc(cc2)O)C[C@H]1c1ccc(C)cc1)C(=O)C
107438 N1(N=C(c2ccc(cc2)O)C[C@H]1c1ccc(C)cc1)C(=O)C
107442 N1(N=C(c2c(cc(cc2)O)O)C[C@H]1c1ccc(cc1)C)C(=O)C
107450 N1(N=C(c2c(cc(cc2)O)O)C[C@H]1c1ccc(cc1)C)C(=O)C
107896 c1(c(cc(cc1C)C)C)[C@H](c1ccccc1)O
107898 c1([C@@H](OC(=O)C)c2ccccc2)c(cc(cc1C)C)C
107899 c1(c(cc(cc1C)C)C)[C@H](c1ccc(C)cc1)O
107901 c1([C@@H](OC(=O)C)c2ccc(C)cc2)c(cc(cc1C)C)C
107902 c1(c(cc(cc1C)C)C)[C@H](c1ccc(OC)cc1)O
107904 c1([C@@H](OC(=O)C)c2ccc(OC)cc2)c(cc(cc1C)C)C
107905 [N+](c1ccc([C@@H](c2c(cc(C)cc2C)C)O)cc1)([O–])=O
107907 [N+](c1ccc([C@@H](c2c(cc(C)cc2C)C)OC(=O)C)cc1)([O–])=O
108066 C(\[C@](c1ccccc1)(O)C)=C\c1ccccc1
110306 C([C@@H](C(=O)OCC)CCCCCC)(N1CCN(c2ncccn2)CC1)=O
110308 C([C@@H](C(=O)OCC)CCCCC)(N1CCN(c2ncccn2)CC1)=O
110310 C([C@@H](C(=O)OCC)CCC)(N1CCN(c2ncccn2)CC1)=O
110322 C([C@@H](C(=O)OCC)CCCC)(N1CCN(c2ncccn2)CC1)=O
110340 C([C@@H](C(=O)OCC)CCCCCCC)(N1CCN(c2ncccn2)CC1)=O
111538 C(#Cc1ccccc1)[C@@H](c1ccccc1)O
111546 C(#Cc1ccccc1)[C@H](c1cc(OC)ccc1)O
111547 C(#Cc1ccccc1)[C@@H](c1ccc(cc1)OC)O
111548 C(#Cc1ccccc1)[C@H](c1ccc(cc1)F)O
111549 C(#Cc1ccccc1)[C@H](c1cc(C)ccc1)O
111551 C(#Cc1ccccc1)[C@@H](c1ccc(cc1)Br)O
111552 C(#Cc1ccccc1)[C@@H](c1ccc(cc1)C)O
111553 C(#Cc1ccccc1)[C@H](c1c2c(cccc2)ccc1)O
111714 C([C@@H](Cc1ccccc1)C)(=O)OC
111822 N1(C(c2c3c(cccc3)ccc2)=O)[C@@H](CC(C1)(C)C)CC
111823 N1(C(c2c3c(cccc3)ccc2)=O)[C@@H](CC(C1)(C)C)CCC
111824 N1(C(c2c3c(cccc3)ccc2)=O)CC(CC[C@H]1CC)(C)C
112965 N1(C(c2c3c(cccc3)ccc2)=O)[C@@H](CC(C1)(C)C)C
112968 C(N1[C@@H](CCC1)CCC)(c1c2c(cccc2)ccc1)=O
112969 N1(C(c2c3c(cccc3)ccc2)=O)CC(C)(C)CC[C@H]1C
112970 C(N1[C@H](CCC)CCCC1)(c1c2c(cccc2)ccc1)=O
113038 N([C@H](c1occc1)C#N)C(c1ccccc1)c1ccccc1

113169 C(#Cc1ccccc1)[C@](c1ccc(cc1)F)(O)C
113170 C(#Cc1ccccc1)[C@](c1ccc(cc1)Cl)(O)C
113641 C(c1ccc(O[C@H](c2ccccc2)CCNC)cc1)(F)(F)F
113665 n1cn(CC[C@H](Oc2c(cc(cc2)Cl)Cl)c2ccccc2)cc1
113681 C(c1ccc(O[C@@H](CCn2cncc2)c2ccccc2)cc1)(F)(F)F
113705 n1cn(CC[C@H](Oc2ccc(Cl)cc2)c2ccccc2)cc1
113713 n1cn(CC[C@H](Oc2c(cc(cc2)Cl)Cl)c2ccc(cc2)Cl)cc1
114061 C([C@H](C[N+]([O–])=O)c1ccccc1)(C(=O)OC)C(=O)OC
114734 C([C@H](c1ccc(O)cc1)N)(=O)O
115000 C1(O[C@H](COC(=O)c2ccccc2)c2c1cccc2)=O
115395 C(N[C@@H](Cc1ccccc1)COCc1ccccc1)(OC(C)(C)C)=O
115396 c1([C@H](NC(OC(C)(C)C)=O)COCc2ccccc2)nccs1
115521 [N+](c1c([C@@H](CC(=O)C)O)cccc1)([O–])=O
115853 c1(c(cc(cc1)Cl)Cl)[C@H](Cn1cncc1)O
115858 c1(c(cc(cc1)Cl)Cl)[C@H](Cn1cncc1)O
115979 N1(C([C@](c2ccccc2)(CC)C(NC1=O)=O)=O)C
116056 C(N[C@H](C(=O)O)CCC)(=O)OCc1ccccc1
116281 C([C@H](C[N+]([O–])=O)c1ccc(F)cc1)(C(=O)OC)C(=O)OC
116286 C([C@H](C[N+]([O–])=O)c1ccc(OC)cc1)(C(=O)OC)C(=O)OC
116292 C([C@@H](c1sccc1)C[N+]([O–])=O)(C(=O)OC)C(=O)OC
117044 C(N[C@H](C(=O)O)CC(C)C)(=O)OC
117157 N1(N=C(c2ccc(cc2)F)C[C@H]1c1ccc(cc1)Cl)C(=S)N
117174 N1(N=C(c2ccc(cc2)F)C[C@H]1c1ccc(cc1)C)C(=S)N
117191 N1(N=C(c2ccc(cc2)F)C[C@H]1c1occc1)C(=S)N
117918 C1(c2c(cccc2)CC[C@@H]1ONc1ccccc1)=O
117952 C(#Cc1ccccc1)[C@@](c1cc(OC)ccc1)(O)C
118245 C(#Cc1ccccc1)[C@@H](c1ccc(cc1)Cl)O
118705 N1c2c(CC[C@H]1c1ccccc1)cccc2
119568 C([C@H](Cc1ccc(cc1)F)O)(=O)O
119569 C([C@H](Cc1ccc(cc1)Cl)O)(=O)O
119570 C([C@H](Cc1ccc(cc1)Br)O)(=O)O
119571 C([C@@H](Cc1ccc(O)cc1)O)(=O)O
121326 C(C(OC(C)C)=O)(C(OC(C)C)=O)[C@@H](C[N+]([O–])=O)c1ccccc1
121348 C([C@@H](C[N+]([O–])=O)c1ccc(F)cc1)(C(=O)OCC)C(=O)OCC
123961 C([C@H](C[N+]([O–])=O)c1ccc(Cl)cc1)(C(=O)OC)C(=O)OC
123962 C([C@H](C[N+]([O–])=O)c1ccc(Br)cc1)(C(=O)OC)C(=O)OC
124149 C(N[C@H](C(=O)O)CCC)(=S)NCCC
124150 C(N[C@H](C(=O)O)CCC)(=S)NCCCC
124159 C(NC(C(=O)O)CCCC)(=S)NCCC
124160 C(N[C@H](C(=O)O)CCCC)(=S)NCCCC
124197 C(NC(C(=O)O)CCc1ccccc1)(=S)NC
124198 C(NC(C(=O)O)CCc1ccccc1)(=S)NCC
124199 C(NC(C(=O)O)CCc1ccccc1)(=S)NCCC

124200 C(NC(C(=O)O)CCc1ccccc1)(=S)NCCCC
124227 c1(c[nH]c2c1cccc2)C[C@H](NC(=S)NC)C(=O)O
124228 c1(c[nH]c2c1cccc2)C[C@H](NC(=S)NCC)C(=O)O
124229 c1(c[nH]c2c1cccc2)C[C@H](NC(=S)NCCC)C(=O)O
124230 c1(c[nH]c2c1cccc2)C[C@H](NC(=S)NCCCC)C(=O)O
124278 N1(C(=S)NCCC)[C@@H](C(=O)O)CCCC1
124279 N1(C(=S)NCCCC)[C@@H](C(=O)O)CCCC1
124297 C(N[C@H](C(=O)O)CCC(=O)O)(=S)NCCC
124298 C(N[C@H](C(=O)O)CCC(=O)O)(=S)NCCCC
125998 c1([C@H](CC(=O)c2ccccc2)O)c2c(cccc2)ccc1
126051 C(C(OC(C)(C)C)=O)(C(OC(C)(C)C)=O)[C@H](C[N+]([O–])=O)c1ccccc1
126055 C([C@H](C[N+]([O–])=O)c1ccc(C)cc1)(C(=O)OCC)C(=O)OCC
126056 C([C@@H](C[N+]([O–])=O)c1ccc(OC)cc1)(C(=O)OCC)C(=O)OCC
126057 C([C@H](C[N+]([O–])=O)c1ccc(Br)cc1)(C(=O)OCC)C(=O)OCC
126058 C([C@@H](c1c(Cl)cccc1)C[N+]([O–])=O)(C(=O)OCC)C(=O)OCC
126062 C([C@@H](c1occc1)C[N+]([O–])=O)(C(=O)OCC)C(=O)OCC
126248 C(#Cc1ccccc1)[C@](c1ccccc1)(O)C
126252 C(#Cc1ccccc1)[C@](c1cc(Cl)ccc1)(O)C
128036 C([C@H](CC(=O)C)c1ccccc1)(C(=O)OC)C(=O)OC
128037 C([C@H](CC(=O)C)c1ccccc1)(C(=O)OCC)C(=O)OCC
128038 C(C(OC(C)C)=O)(C(OC(C)C)=O)[C@H](CC(=O)C)c1ccccc1
128043 C([C@@H](c1c(C(F)(F)F)cccc1)CC(=O)C)(C(=O)OCC)C(=O)OCC
128044 C([C@@H](c1occc1)CC(=O)C)(C(=O)OCC)C(=O)OCC
128661 c1(c[nH]c2c1cccc2)C[C@@H](C(=O)OC)N
128667 c1(c[nH]c2c1cccc2)C[C@H](C(=O)OCC)N
128685 c1(c[nH]c2c1cccc2)C[C@H](N)CO
128806 C([C@H](c1ccccc1)O)(=O)OC
128915 C(N[C@H](C(=O)O)C(C)C)(=O)N
128921 C(N[C@H](C(=O)O)CO)(=O)N
129823 N1(N[C@H](c2cc3OCOc3cc2CC1=O)c1ccc(cc1)N)C(=O)NC
129829 N1(N[C@H](c2cc3OCOc3cc2CC1=O)c1ccc(cc1)N)C(=O)NCC
129835 N1(N[C@H](c2cc3OCOc3cc2CC1=O)c1ccc(cc1)N)C(=O)NCCC
130406 C(N[C@H](C(=O)O)CCC)(Nc1cc(cc(c1)Cl)Cl)=S
130463 C(N[C@H](C(=O)O)CO)(Nc1c(c(Cl)ccc1)Cl)=S
130464 C(N[C@H](C(=O)O)CO)(Nc1cc(cc(c1)Cl)Cl)=S
130484 C(N[C@H](C(=O)O)Cc1ccccc1)(Nc1c(cc(cc1)F)F)=S
130487 C(N[C@H](C(=O)O)Cc1ccccc1)(Nc1c(cc(cc1)F)F)=O
130522 c1(c[nH]c2c1cccc2)C[C@H](NC(Nc1c(cc(cc1)F)F)=S)C(=O)O
130525 c1(c[nH]c2c1cccc2)C[C@H](NC(Nc1c(cc(cc1)F)F)=O)C(=O)O
130531 c1(c2c(ccc(c2)C)[nH]c1)C[C@H](NC(Nc1c(cc(cc1)F)F)=S)C(=O)O
130533 c1(c2c(ccc(c2)C)[nH]c1)C[C@H](NC(Nc1c(cc(cc1)F)F)=O)C(=O)O
130538 C(N[C@H](C(=O)O)Cc1ccc(O)cc1)(Nc1c(cc(cc1)F)F)=S
130541 C(N[C@H](C(=O)O)Cc1ccc(O)cc1)(Nc1c(cc(cc1)F)F)=O

130547 C(N[C@H](C(=O)O)Cc1cc(O)ccc1)(Nc1c(cc(cc1)F)F)=S
130549 C(N[C@H](C(=O)O)Cc1cc(O)ccc1)(Nc1c(cc(cc1)F)F)=O
130553 C(N[C@H](CC(=O)O)c1ccccc1)(Nc1c(cc(cc1)F)F)=S
130555 C(NC(CC(=O)O)c1ccccc1)(Nc1c(cc(cc1)F)F)=O
130561 C(Nc1c(cc(cc1)F)F)(N[C@H](C(=O)O)CC)=S
130590 C(N[C@@H](C(=O)O)CC=C)(Nc1cc(cc(c1)Cl)Cl)=S
130592 C(N1[C@H](C(=O)O)CCC1)(Nc1c(cc(cc1)F)F)=S
130593 C(N1[C@H](C(=O)O)CCC1)(Nc1c(c(Cl)ccc1)Cl)=S
130594 C(N1[C@H](C(=O)O)CCC1)(Nc1cc(Cl)cc(c1)Cl)=S
130596 C(N1C[C@@H](C(=O)O)CCC1)(Nc1c(cc(cc1)F)F)=S
130598 C(N1[C@@H](C(=O)O)CCCC1)(Nc1c(cc(cc1)F)F)=S
130613 C(Nc1c(c(Cl)ccc1)Cl)(N[C@@H](CC(=O)O)C(=O)O)=S
130614 C(N[C@@H](CC(=O)O)C(=O)O)(Nc1cc(cc(c1)Cl)Cl)=S
135510 C([C@@H](NC(=O)C)CC(C)C)(=O)O
136160 C([C@H](Oc1ccc(C)cc1)CC)(=O)O
136218 C(#Cc1ccccc1)[C@](c1ccc(cc1)Br)(O)C
136221 C(#Cc1ccccc1)[C@@](c1cc(C)ccc1)(O)C
136224 C(#Cc1ccccc1)[C@@](c1ccc(cc1)C)(O)C
136758 N1(C([C@@](c2ccccc2)(C)C(NC1=O)=O)=O)C
136773 N1(C([C@@](c2ccccc2)(CCC)C(NC1=O)=O)=O)C
136779 N1(C([C@@](c2ccccc2)(CCC)C(NC1=O)=O)=O)C
136786 N1(C([C@@](C2=CCCCC2)(CC)C(NC1=O)=O)=O)C
136787 N1(C([C@@](c2ccccc2)(CCCC)C(NC1=O)=O)=O)C
136793 N1(C([C@@](c2ccccc2)(CCCC)C(NC1=O)=O)=O)C
136807 N1(C([C@@](C2=CCCCC2)(C)C(NC1=O)=O)=O)CC
136814 N1(C([C@](C2=CCCCC2)(C)C(NC1=O)=O)=O)CCC
136938 N=1C(C(Nc2c(C1c1ccccc1)cc(Cl)cc2)=O)(Cc1ccccc1)CO
136942 N1=C(c2c(ccc(c2)Cl)NC([C@H]1CO)=O)c1ccccc1
139143 C(#Cc1ccccc1)[C@@H](c1cc2ccccc2cc1)O
139147 C(#Cc1ccccc1)[C@@](c1ccc(cc1)OC)(O)C
139706 C12=C(NC(=O)N[C@@H]1c1ccccc1)CC(CC2=O)(C)C
139714 C12=C(NC(=O)N[C@@H]1c1c(Cl)cccc1)CC(CC2=O)(C)C
139722 C12=C(NC(=O)N[C@@H]1c1cc(Cl)ccc1)CC(CC2=O)(C)C
139730 C12=C(NC(=O)N[C@@H]1c1ccc(Cl)cc1)CC(CC2=O)(C)C
139738 C12=C(NC(=O)N[C@@H]1c1c(Br)cccc1)CC(CC2=O)(C)C
139746 C12=C(NC(=O)N[C@@H]1c1cc(Br)ccc1)CC(CC2=O)(C)C
139754 C12=C(NC(=O)N[C@@H]1c1ccc(Br)cc1)CC(CC2=O)(C)C
139762 C12=C(NC(=O)N[C@@H]1c1c(C)cccc1)CC(CC2=O)(C)C
139770 C12=C(NC(=O)N[C@@H]1c1cc(C)ccc1)CC(CC2=O)(C)C
139778 C12=C(NC(=O)N[C@@H]1c1ccc(C)cc1)CC(CC2=O)(C)C
139788 C12=C(NC(=O)N[C@@H]1c1c(OC)cccc1)CC(CC2=O)(C)C
139796 C12=C(NC(=O)N[C@@H]1c1cc(OC)ccc1)CC(CC2=O)(C)C
139810 C12=C(N(C(=O)N[C@@H]1c1ccccc1)C)CC(CC2=O)(C)C

139812 C12=C(N(C(=O)N[C@@H]1c1c(Cl)cccc1)C)CC(CC2=O)(C)C
139814 C12=C(N(C(=O)N[C@@H]1c1cc(Cl)ccc1)C)CC(CC2=O)(C)C
139816 C12=C(N(C(=O)N[C@@H]1c1ccc(Cl)cc1)C)CC(CC2=O)(C)C
139818 C12=C(N(C(=O)N[C@@H]1c1c(Br)cccc1)C)CC(CC2=O)(C)C
139820 C12=C(N(C(=O)N[C@@H]1c1cc(Br)ccc1)C)CC(CC2=O)(C)C
139822 C12=C(N(C(=O)N[C@@H]1c1ccc(Br)cc1)C)CC(CC2=O)(C)C
139824 C12=C(N(C(=O)N[C@@H]1c1c(C)cccc1)C)CC(CC2=O)(C)C
139826 C12=C(N(C(=O)N[C@@H]1c1cc(C)ccc1)C)CC(CC2=O)(C)C
139828 C12=C(N(C(=O)N[C@@H]1c1ccc(C)cc1)C)CC(CC2=O)(C)C
139830 C12=C(N(C(=O)N[C@@H]1c1c(OC)cccc1)C)CC(CC2=O)(C)C
139832 C12=C(N(C(=O)N[C@@H]1c1cc(OC)ccc1)C)CC(CC2=O)(C)C
141244 c1([C@H](CC(=O)C)O)c(Br)cccc1
141368 C([C@@H](Cc1ccccc1)C)(=O)O
142694 N([C@](C(OC(C)(C)C)=O)(C#N)c1ccccc1)(C(=O)OC)NC(=O)OC
142697 N([C@](C(OC(C)(C)C)=O)(C#N)c1ccc(OC)cc1)(C(=O)OC)NC(=O)OC
142698 N([C@](C(OC(C)(C)C)=O)(C#N)c1ccc(Cl)cc1)(C(=O)OC)NC(=O)OC
142699 N([C@](C(OC(C)(C)C)=O)(C#N)c1ccc(C)cc1)(C(=O)OC)NC(=O)OC
142955 C(N[C@@H](C(OC(C)C)=O)Cc1ccccc1)(OC(C)(C)C)=O
143387 C1([C@@](Cc2ccccc2)(O)CCCC1)=O
143517 C(#Cc1ccccc1)[C@@H](c1cc(Cl)ccc1)O
143703 C1(=C(N(C=C[C@H]1c1ccc([N+]([O–])=O)cc1)c1ccc(OC)cc1)C)C(=O)OCC
143704 C1(=C(N(C=C[C@H]1c1ccc([N+]([O–])=O)cc1)c1cc(OC)ccc1)C)C(=O)OCC
143710 C1(=C(N(C=C[C@H]1c1ccc([N+]([O–])=O)cc1)c1ccc(OC)cc1)C)C(=O)OC
143713 C1(=C(N(C=C[C@H]1c1ccc([N+]([O–])=O)cc1)c1cc(OC)ccc1)C)C(=O)OC
143719 C1(=C(N(C=C[C@H]1c1c([N+]([O–])=O)cccc1)c1ccc(cc1)OC)C)C(=O)OCC
143720 C1(=C(N(C=C[C@H]1c1c([N+]([O–])=O)cccc1)c1cc(OC)ccc1)C)C(=O)OCC
143721 C1(=C(N(C=C[C@H]1c1c([N+]([O–])=O)cccc1)c1ccc(cc1)OC)C)C(=O)OC
143722 C1(=C(N(C=C[C@H]1c1c([N+]([O–])=O)cccc1)c1cc(OC)ccc1)C)C(=O)OC
145713 [N+](c1c([C@H](CC(=O)CC)O)cccc1)([O–])=O
146171 P(C[C@@H]1C(=O)CCC1)(=O)(OCC)OCC
146173 P(C[C@@H]1C(=O)COC1)(=O)(OCC)OCC
147344 C([C@H](NC(=O)CN)CO)(=O)O
147350 C([C@H](NC(=O)CN)C(C)C)(=O)O
148538 C(Nc1c(OCCCCCCC)cccc1)(O[C@@H](CN1CCN(C)CC1)COC)=O
148568 C(Nc1ccc(OCCC)cc1)(O[C@@H](CN1CCN(C)CC1)COC)=O
148574 C(Nc1ccc(OCCCC)cc1)(O[C@@H](CN1CCN(C)CC1)COC)=O
151693 C(C(=O)c1ccccc1)[C@@H](c1cc2ccccc2cc1)O
152426 N1C(c2ccccc2N[C@@H]1c1ccccc1)=O
152820 C(N[C@H](C(=O)OC)c1ccccc1)(=O)OCc1ccccc1
158126 C([C@@H](c1ccc(Cl)cc1)CC(=O)C)(C(=O)OCC)C(=O)OCC
158127 C([C@@H](c1ccc(Br)cc1)CC(=O)C)(C(=O)OCC)C(=O)OCC
158131 C([C@@H](c1ccc(C)cc1)CC(=O)C)(C(=O)OCC)C(=O)OCC
158134 C([C@@H](c1ccc(OC)cc1)CC(=O)C)(C(=O)OCC)C(=O)OCC

158137 C([C@@H](c1sccc1)CC(=O)C)(C(=O)OCC)C(=O)OCC
158258 c1([C@@H]2CC(=O)CC2)ccc(cc1)OC
162728 C([C@@H](C[N+]([O−])=O)c1ccccc1)(C(=O)OCC)(C(=O)OCC)F
164148 N(C(c1ccccc1)c1ccccc1)[C@@H](C#N)c1ccc(OC)cc1
164151 N([C@@H](c1sccc1)C#N)C(c1ccccc1)c1ccccc1
164295 C1(=C(NC=2CC(C)(C)CC(C2[C@H]1c1ccccc1)=O)C)C(=O)OCC
164296 C1(=C(NC=2CC(C)(C)CC(C2[C@H]1c1ccc(cc1)Br)=O)C)C(=O)OCC
164304 C1(=C(NC=2CC(C)(C)CC(C2[C@H]1c1ccc(C#N)cc1)=O)C)C(=O)OCC
164386 c1([C@@H]2CC(=O)CC2)cc(Br)ccc1
164387 c1([C@H]2CC(=O)CC2)ccc(cc1)F
164494 C1([C@](c2ccccc2N1)(c1ccc(cc1)C)C)=O
164495 C1([C@](c2ccccc2N1)(c1ccc(cc1)C)CC)=O
164732 C(C(OC(C)(C)C)=O)(C(OC(C)(C)C)=O)[C@@H](c1cc(OC)c(cc1)OC)C[N+]([O−])=O
164736 C([C@@H](c1occc1)C[N+]([O−])=O)(C(OC(C)(C)C)=O)C(OC(C)(C)C)=O
165840 C([C@H](c1c(Cl)cccc1)C[N+]([O−])=O)(C(=O)OC)C(=O)OC
166222 C([C@@H](C[N+]([O−])=O)c1ccc(Cl)cc1)(C(=O)OCC)C(=O)OCC
166223 C([C@H](c1c(C(F)(F)F)cccc1)C[N+]([O−])=O)(C(=O)OCC)C(=O)OCC
166224 C([C@H](c1c(cc(cc1)Cl)Cl)C[N+]([O−])=O)(C(=O)OCC)C(=O)OCC
168178 c1(n(c2ccc(C([C@@H](N3CCN(c4cc(C(F)(F)F)ccc4)CC3)C)=O)cc2o1)C)=O
168190 c1(n(c2ccc(C([C@@H](N3CCN(CC3)c3ccc(cc3)F)C)=O)cc2o1)C)=O
168195 c1([nH]c2ccc(C([C@@H](N3CCN(c4cc(C(F)(F)F)ccc4)CC3)C)=O)cc2o1)=O
168206 c1([nH]c2ccc(C([C@@H](N3CCN(CC3)c3ccc(cc3)F)C)=O)cc2o1)=O
168470 C(N[C@@H](C(=O)O)CC(C)C)(OC(C)(C)C)=O
168530 C(N[C@H](C(=O)OCC)CC(C)C)(OC(C)(C)C)=O
168788 N1(c2c(OC)cccc2)CCN(CC1)C[C@@H](COc1c2c(cccc2)ccc1)O
168797 N1(c2ccc(cc2)O)CCN(CC1)C[C@@H](COc1c2c(cccc2)ccc1)O
168805 N1(c2ccc(cc2)OC)CCN(CC1)C[C@@H](COc1c2c(cccc2)ccc1)O
168813 N1(c2ccc(cc2)OCC)CCN(CC1)C[C@@H](COc1c2c(cccc2)ccc1)O
168829 N1(c2ccc(cc2)Cl)CCN(CC1)C[C@@H](COc1c2c(cccc2)ccc1)O
168845 N1(c2cc(OC)ccc2)CCN(C[C@@H](COc2c3c(cccc3)ccc2)O)CC1
168853 N1(c2cc(OCC)ccc2)CCN(C[C@@H](COc2c3c(cccc3)ccc2)O)CC1
168861 N1(c2cc(Cl)ccc2)CCN(C[C@@H](COc2c3c(cccc3)ccc2)O)CC1
169507 C1(S(=O)(=O)c2ccccc2)(S(=O)(=O)c2ccccc2)CC(=C)[C@H](C1)C(OC)(C)C
169508 C1(S(=O)(=O)c2ccccc2)(S(=O)(=O)c2ccccc2)C\C(=C\c2ccccc2)[C@H](C1)C(OC)(C)C
171775 c12c([nH]cc2)cccc1OC[C@H](CNC(C)C)O
176561 n1c(Nc2cc(OC(C)C)[nH]n2)c(Cl)cnc1N[C@H](c1ncc(cn1)F)C
180115 C(c1cc(C(F)(F)F)cc(NNC([C@H](N2CCN(CC2)C)c2c(F)cccc2)=O)c1)(F)(F)F
180120 c1([C@H](C(NNc2cc(C(F)(F)F)cc(C(F)(F)F)c2)=O)N2CCN(C)CC2)c(c(F)ccc1)F
180122 c1([C@H](C(NNc2cc(C(F)(F)F)cc(C(F)(F)F)c2)=O)N2CCN(CC2)C)c(F)cccc1F
181223 C(C(OC(C)(C)C)=O)(c1n(ccc1)CC)C(C(=O)OCC)C(=O)OCC
181229 C([C@H](c1c(cc[nH]1)C)C(C(=O)OCC)C(=O)OCC)(OC(C)(C)C)=O
181297 C([C@@H](c1cc(Cl)c(cc1)Cl)C[N+]([O−])=O)(C(=O)OCC)C(=O)OCC
181530 C(C(=O)OCC)[C@@H](c1ccccc1)O

183380 c12nc(n3c(ncc3)c3ccccc3)ncc1N(C(=O)[C@@]1(N2CCCC1)CC)C
183381 c12nc(n3c(ncc3)c3cc(F)ccc3)ncc1N(C(=O)[C@@]1(N2CCCC1)CC)C
183384 c12nc(n3cncc3)ncc1N(C(=O)[C@@]1(N2CCCC1)CC)C
188645 c1([C@H](OC(=O)C)Cn2cncc2)c(cc(cc1)Cl)Cl
189350 n1c(Nc2n[nH]c(c2)C)c(Cl)cnc1N[C@H](c1ncc(cn1)F)C
197212 C(O[C@H](C#N)c1ccccc1)(=O)c1ccccc1
197558 c1(C([C@H](OCc2cc3ccccc3cc2)Cc2ccccc2)=O)n(ccn1)C
197564 c1(C([C@H](OCc2cc3ccccc3cc2)Cc2ccc(C(C)(C)C)cc2)=O)n(ccn1)C
197567 C([C@@H](OCc1cc2ccccc2cc1)Cc1ccccc1)(=O)OC
197568 C([C@@H](OCc1cc2ccccc2cc1)Cc1ccc(C(C)(C)C)cc1)(=O)OC
198803 c1([C@](c2ccccc2)(O)C)c(C)cccc1
198809 c1([C@@](c2ccccc2)(O)C)c(OC)cccc1
198811 c1([C@@](c2ccccc2)(O)C)ccc(cc1)OC
199352 [N+](c1c([C@@H](CC(=O)CO)O)cccc1)([O–])=O
200523 C(N[C@@H](C(C#N)C#N)c1c(C)cccc1)(OC(C)(C)C)=O
200524 C(N[C@@H](C(C#N)C#N)c1ccc(cc1)C)(OC(C)(C)C)=O
200685 c1([C@@]2(C(Nc3c2cccc3)=O)Cc2ccccc2)c(oc(c1)c1ccccc1)c1ccccc1
200686 c1([C@@]2(C(Nc3c2cccc3)=O)Cc2ccccc2)c(oc(c1)c1ccc(cc1)F)c1ccc(F)cc1
200687 c1([C@@]2(C(Nc3c2cccc3)=O)Cc2ccccc2)c(oc(c1)c1ccc(cc1)Cl)c1ccc(Cl)cc1
200688 c1([C@@]2(C(Nc3c2cccc3)=O)Cc2ccccc2)c(oc(c1)c1ccc(cc1)Br)c1ccc(Br)cc1
200689 c1([C@@]2(C(Nc3c2cccc3)=O)Cc2ccccc2)c(oc(c1)c1cc(Cl)ccc1)c1cc(Cl)ccc1
200691 c1([C@@]2(C(Nc3c2cccc3)=O)Cc2ccccc2)c(oc(c1)c1cc(OC)ccc1)c1cc(OC)ccc1
200692 c1([C@@]2(C(Nc3c2cccc3)=O)Cc2ccccc2)c(oc(c1)c1cc2ccccc2cc1)c1cc2ccccc2cc1
200694 c1([C@@]2(C(Nc3c2cccc3)=O)Cc2cc(F)ccc2)c(oc(c1)c1ccccc1)c1ccccc1
200695 c1([C@@]2(C(Nc3c2cccc3)=O)Cc2c(F)cccc2)c(oc(c1)c1ccccc1)c1ccccc1
200696 c1([C@@]2(C(Nc3c2cccc3)=O)Cc2cc(C)ccc2)c(oc(c1)c1ccccc1)c1ccccc1
200697 c1([C@@]2(C(Nc3c2cccc3)=O)Cc2c(OC)cccc2)c(oc(c1)c1ccccc1)c1ccccc1
200698 c1([C@@]2(C(Nc3c2cccc3)=O)Cc2ccc(cc2)OC)c(oc(c1)c1ccccc1)c1ccccc1
200705 c1([C@@]2(C(Nc3c2cccc3)=O)Cc2ccccc2)c([nH]c(c1)c1ccccc1)c1ccccc1
200706 c1([C@@]2(C(Nc3c2cccc3)=O)Cc2ccccc2)c([nH]c(c1)c1ccc(cc1)F)c1ccc(F)cc1
200707 c1([C@@]2(C(Nc3c2cccc3)=O)Cc2ccccc2)c([nH]c(c1)c1ccc(cc1)Cl)c1ccc(Cl)cc1
200708 c1([C@@]2(C(Nc3c2cccc3)=O)Cc2ccccc2)c([nH]c(c1)c1ccc(cc1)Br)c1ccc(Br)cc1
200709 c1([C@@]2(C(Nc3c2cccc3)=O)Cc2ccccc2)c([nH]c(c1)c1cc(Cl)ccc1)c1cc(Cl)ccc1
200710 c1([C@@]2(C(Nc3c2cccc3)=O)Cc2ccccc2)c([nH]c(c1)c1cc(OC)ccc1)c1cc(OC)ccc1
200711 c1([C@@]2(C(Nc3c2cccc3)=O)Cc2ccccc2)c([nH]c(c1)c1cc2ccccc2cc1)c1cc2ccccc2cc1
200712 c1([C@@]2(C(Nc3c2cccc3)=O)Cc2cc(F)ccc2)c([nH]c(c1)c1ccccc1)c1ccccc1
200714 c1([C@@]2(C(Nc3c2cccc3)=O)Cc2cc(C)ccc2)c([nH]c(c1)c1ccccc1)c1ccccc1
200715 c1([C@@]2(C(Nc3c2cccc3)=O)Cc2c(OC)cccc2)c([nH]c(c1)c1ccccc1)c1ccccc1
200716 c1([C@@]2(C(Nc3c2cccc3)=O)Cc2ccc(cc2)OC)c([nH]c(c1)c1ccccc1)c1ccccc1
200718 c1([C@@]2(C(Nc3c2cccc3)=O)Cc2c3c(cccc3)ccc2)c([nH]c(c1)c1ccccc1)c1ccccc1
200720 c1([C@@]2(C(Nc3c2cccc3)=O)C)c([nH]c(c1)c1ccccc1)c1ccccc1
200721 c1([C@@]2(C(Nc3c2cccc3)=O)CCCC)c([nH]c(c1)c1ccccc1)c1ccccc1
200723 c1([C@@]2(C(Nc3c2cccc3)=O)Cc2c(F)cccc2)c([nH]c(c1)c1ccccc1)c1ccccc1

200749 C(N[C@@H](C(=O)OCC)Cc1ccccc1)(OC(C)(C)C)=O
200900 c12c(c3ccccc3[nH]1)CC[N@@]([C@H]2c1ccc(Br)cc1)Cc1c2c(cccc2)ccc1
200913 c12c(c3ccccc3[nH]1)CC[N@@]([C@H]2c1ccccc1)Cc1c2c(cccc2)ccc1
200914 c12c(c3ccccc3[nH]1)CC[N@@]([C@H]2c1ccc(OC)cc1)Cc1c2c(cccc2)ccc1
200966 C([C@@H](CC(=O)OC)c1ccccc1)(C(=O)OC)C(=O)OC
200977 C([C@@H](CC(=O)OC)c1ccccc1)(C(=O)OCC)C(=O)OCC
201588 N1([C@H](c2ccccc2CC1)Cc1ccccc1)C(=O)OC
202955 C1([C@@](c2ccccc2)(O)CCCC1)=O
204377 C=1([C@]2(C(Nc3c2cccc3OC)=O)C(OC1C)=O)[Si](C(C)C)(C(C)C)C(C)C
204378 C=1([C@]2(C(Nc3c2cc(cc3)Br)=O)C(OC1C)=O)[Si](C(C)C)(C(C)C)C(C)C
205423 C1([C@](c2ccccc2)(CO)CCCC1)=O
206419 C([C@H]1[N@@](NC(=O)c2ccccc2)CCc2ccccc12)#Cc1ccccc1
206421 C([C@H]1[N@@](NC(=O)c2ccccc2)CCc2cc(C)ccc12)#Cc1ccccc1
206424 C([C@H]1[N@@](NC(=O)c2ccccc2)CCc2cc(Br)ccc12)#Cc1ccccc1
206426 C([C@H]1[N@@](NC(=O)c2ccccc2)CCc2cc(OC)ccc12)#Cc1ccccc1
206428 C([C@H]1[N@@](NC(=O)c2ccccc2)CCc2ccccc12)#Cc1c(C)cccc1
206429 C([C@H]1[N@@](NC(=O)c2ccccc2)CCc2ccccc12)#Cc1cc(C)ccc1
206430 C([C@H]1[N@@](NC(=O)c2ccccc2)CCc2ccccc12)#Cc1ccc(C)cc1
206432 C([C@H]1[N@@](NC(=O)c2ccccc2)CCc2ccccc12)#Cc1ccc(OC)cc1
206433 C([C@H]1[N@@](NC(=O)c2ccccc2)CCc2ccccc12)#CC1=CCCCC1
206435 C([C@H]1[N@@](NC(=O)c2ccccc2)CCc2ccccc12)#CC1CC1
206436 C([C@H]1[N@@](NC(=O)c2ccccc2)CCc2ccccc12)#CC1CCCC1
206438 C([C@]1([N@@](NC(=O)c2ccccc2)CCc2c1cccc2)C)#Cc1ccccc1
206439 C([C@]1([N@@](NC(=O)c2ccccc2)CCc2c1cccc2)C)#Cc1ccc(C)cc1
206441 C([C@]1([N@@](NC(=O)c2ccccc2)CCc2c1cccc2)C)#Cc1ccc(OC)cc1
206442 C([C@]1([N@@](NC(=O)c2ccccc2)CCc2c1cccc2)C)#CC1=CCCCC1
206443 C([C@]1([N@@](NC(=O)c2ccccc2)CCc2c1cccc2)C)#CC1CC1
206444 C([C@]1([N@@](NC(=O)c2ccccc2)CCc2c1cccc2)C)#CC1CCCC1
206707 c1([C@H]2CC(=O)CC2)ccc(cc1)Br
206709 c1([C@H]2CC(=O)CC2)ccc(C(=O)C)cc1
208360 C(\[C@@](C(OC(C)(C)C)=O)(C#N)c1ccccc1)(=C\C(=O)OC)C(=O)OC
208361 C(\[C@@](C(OC(C)(C)C)=O)(C#N)c1ccc(OC)cc1)(=C\C(=O)OC)C(=O)OC
208362 C(\[C@@](C(OC(C)(C)C)=O)(C#N)c1ccc(C)cc1)(=C\C(=O)OC)C(=O)OC
208363 C(\[C@@](C(OC(C)(C)C)=O)(C#N)c1ccc(Cl)cc1)(=C\C(=O)OC)C(=O)OC
209489
C(C(Oc1ccccc1)=O)(C(Oc1ccccc1)=O)[C@@H](c1cc(C(C)(C)C)c(c(c1)C(C)(C)C)O)c1ccc([N+]([O–])=O)cc1
209491 C(C(Oc1ccccc1)=O)(C(Oc1ccccc1)=O)[C@@H](c1cc(c(O)c(c1)C(C)(C)C)C(C)(C)C)c1cc(Br)ccc1
209494
C(C(Oc1ccccc1)=O)(C(Oc1ccccc1)=O)[C@@H](c1cc(c(O)c(c1)C(C)(C)C)C(C)(C)C)c1cc(OC)c(cc1)OC
209495
C([C@@H](c1cc(c(O)c(c1)C(C)(C)C)C(C)(C)C)c1c2c(cccc2)ccc1)(C(Oc1ccccc1)=O)C(Oc1ccccc1)=O
209498 C(C(Oc1ccccc1)=O)(C(Oc1ccccc1)=O)[C@@H](c1cc(C(C)(C)C)c(c(c1)C(C)(C)C)O)C(C)(C)C

209806 O1c2c(CC[C@H]1c1ccccc1)cccc2
210534 c1(NC[C@@H](c2ccccc2)OC)c(cc(F)cc1F)F
210535 c1(c(c(F)c(c(c1F)F)F)F)NC[C@@H](c1ccccc1)OC
210536 c1(NC[C@@H](c2ccccc2)O)c(cc(F)cc1F)F
210688 c12[C@@H](NCCc2cc(c(c1)OC)OC)c1ccccc1
210695 c12[C@@H](c3c(C)cccc3)NCCc2cc(c(c1)OC)OC
210696 c12[C@@H](c3c(OC)cccc3)NCCc2cc(c(c1)OC)OC
210698 c12[C@@H](c3c(cc(cc3)OC)OC)NCCc2cc(c(c1)OC)OC
210701 c12[C@@H](c3c(Cl)cccc3)NCCc2cc(c(c1)OC)OC
210702 c12[C@@H](c3c(Br)cccc3)NCCc2cc(c(c1)OC)OC
210706 c1([C@@H]2c3c(cccc3)CCN2)c(Cl)cccc1
210709 c12[C@@H](c3c(Cl)cccc3)NCCc1cc(cc2)OC
210710 c12[C@@H](c3c(C)cccc3)NCCc1ccc(c2)OC
210711 c12[C@@H](c3c(OC)cccc3)NCCc1ccc(c2)OC
210712 c12[C@@H](c3c(Cl)cccc3)NCCc1ccc(c2)OC
210713 c12[C@@H](c3c(Br)cccc3)NCCc1ccc(c2)OC
211297 N1([C@@H](Cc2c(C1)cccc2)c1c(C)cccc1)C(=O)C
211972 C1(N(c2ccccc2[C@@]1(c1ccccc1)CC)C)=O
212400 C1([C@](c2cc3ccccc3cc2)(\C=C/C(=O)OCC)c2c(cccc2)N1)=O
212411 C1([C@@](C#CC(=O)OCC)(c2cc3ccccc3cc2)c2c(cccc2)N1)=O
216325 [nH]1c2c(c(OC[C@H](CNC(C)C)O)ccc2)c2c1cccc2
216463 c12c(c3c(cc(OC)cc3)oc1ccc(C(N[C@H](CC(C)C)CO)=O)c2)=O
216479 c12c(c3c(cc(OC)cc3)oc1ccc(C(N[C@H](C(C)C)CO)=O)c2)=O
216493 c12c(c3ccc(cc3oc1ccc(C(N[C@@H](CO)C)=O)c2)OC)=O
216502 c12c(c3ccc(cc3oc1ccc(C(NC[C@H](O)C)=O)c2)OC)=O

2. **Statistics for Each Cluster**

CSP CLUSTER ZSCORE NPAIR MEANDIFF STDEVDIFF
2966 1 4.43 2 0.64 0.04
2966 2 4.27 1 0.77 0.00
2966 3 4.21 1 0.76 0.00
2966 4 4.17 1 0.75 0.00
23735 1 11.52 12 0.43 0.23
23735 10 6.56 1 0.86 0.00
23735 11 6.54 1 0.86 0.00
23735 12 6.49 1 0.86 0.00
23735 13 6.39 1 0.84 0.00
23735 14 6.29 1 0.83 0.00
23735 15 6.21 1 0.82 0.00
23735 16 6.16 1 0.81 0.00
23735 17 6.16 1 0.81 0.00
23735 18 6.04 1 0.80 0.00
23735 19 6.03 1 0.80 0.00

23735 2 7.75 10 0.31 0.13
23735 20 5.80 1 0.76 0.00
23735 21 5.71 1 0.75 0.00
23735 22 5.71 1 0.75 0.00
23735 23 5.71 1 0.75 0.00
23735 24 5.70 1 0.75 0.00
23735 25 5.68 1 0.75 0.00
23735 26 5.62 2 0.51 0.15
23735 27 5.62 2 0.49 0.24
23735 28 5.61 2 0.49 0.27
23735 29 5.57 2 0.49 0.19
23735 3 7.20 1 0.95 0.00
23735 30 5.56 1 0.73 0.00
23735 31 5.56 1 0.73 0.00
23735 32 5.56 1 0.73 0.00
23735 33 5.55 1 0.73 0.00
23735 34 5.47 1 0.72 0.00
23735 35 5.47 2 0.48 0.08
23735 36 5.46 1 0.72 0.00
23735 37 5.45 1 0.72 0.00
23735 38 5.41 1 0.71 0.00
23735 39 5.34 1 0.70 0.00
23735 4 7.04 1 0.93 0.00
23735 40 5.33 1 0.70 0.00
23735 41 5.32 1 0.70 0.00
23735 42 5.19 1 0.68 0.00
23735 43 5.17 1 0.68 0.00
23735 44 5.14 2 0.46 0.07
23735 45 5.10 5 0.29 0.24
23735 46 5.08 2 0.45 0.24
23735 47 5.05 2 0.45 0.19
23735 48 5.05 1 0.67 0.00
23735 49 5.03 1 0.66 0.00
23735 5 6.93 6 0.35 0.03
23735 50 5.02 1 0.66 0.00
23735 51 5.00 2 0.45 0.43
23735 52 4.95 1 0.65 0.00
23735 53 4.92 1 0.65 0.00
23735 54 4.87 1 0.64 0.00
23735 55 4.86 1 0.64 0.00
23735 56 4.86 2 0.42 0.39
23735 57 4.85 1 0.64 0.00
23735 58 4.84 9 0.21 0.09

23735 59 4.83 2 0.43 0.35
23735 6 6.84 1 0.90 0.00
23735 60 4.82 4 0.31 0.30
23735 61 4.80 4 0.31 0.01
23735 62 4.79 5 0.27 0.11
23735 63 4.79 1 0.63 0.00
23735 64 4.79 2 0.43 0.35
23735 65 4.78 4 0.31 0.16
23735 7 6.81 1 0.90 0.00
23735 8 6.77 1 0.89 0.00
23735 9 6.60 1 0.87 0.00
45167 1 7.84 2 0.76 0.07
45167 10 6.26 1 0.78 0.00
45167 11 6.18 1 0.77 0.00
45167 12 5.84 1 0.72 0.00
45167 13 5.74 1 0.71 0.00
45167 14 5.71 1 0.71 0.00
45167 15 5.70 1 0.71 0.00
45167 16 5.69 1 0.71 0.00
45167 17 5.61 1 0.70 0.00
45167 18 5.60 1 0.69 0.00
45167 19 5.60 1 0.69 0.00
45167 2 7.43 1 0.92 0.00
45167 20 5.52 1 0.68 0.00
45167 21 5.46 1 0.68 0.00
45167 22 5.28 1 0.65 0.00
45167 23 5.18 1 0.64 0.00
45167 24 5.01 1 0.62 0.00
45167 25 4.82 1 0.60 0.00
45167 26 4.76 1 0.59 0.00
45167 27 4.71 1 0.58 0.00
45167 28 4.57 2 0.44 0.29
45167 29 4.47 1 0.55 0.00
45167 3 6.93 1 0.86 0.00
45167 30 4.43 1 0.55 0.00
45167 4 6.64 1 0.82 0.00
45167 5 6.64 1 0.82 0.00
45167 6 6.55 1 0.81 0.00
45167 7 6.37 1 0.79 0.00
45167 8 6.37 1 0.79 0.00
45167 9 6.31 1 0.78 0.00
45172 1 5.23 1 0.59 0.00
45172 10 4.36 1 0.49 0.00

45172 11 4.23 1 0.48 0.00
45172 2 4.90 1 0.55 0.00
45172 3 4.79 1 0.54 0.00
45172 4 4.78 1 0.54 0.00
45172 5 4.71 1 0.53 0.00
45172 6 4.52 1 0.51 0.00
45172 7 4.46 1 0.50 0.00
45172 8 4.43 1 0.50 0.00
45172 9 4.38 1 0.49 0.00
45173 1 8.36 2 0.90 0.01
45173 10 6.67 1 1.01 0.00
45173 11 6.65 2 0.71 0.00
45173 12 6.45 1 0.98 0.00
45173 13 6.45 3 0.54 0.16
45173 14 6.30 1 0.96 0.00
45173 15 6.17 3 0.51 0.09
45173 16 5.86 2 0.62 0.06
45173 17 5.85 2 0.62 0.06
45173 18 5.82 2 0.62 0.06
45173 19 5.76 3 0.48 0.13
45173 2 8.29 12 0.35 0.20
45173 20 5.72 2 0.61 0.29
45173 21 5.70 2 0.61 0.32
45173 22 5.65 2 0.60 0.04
45173 23 5.61 2 0.60 0.26
45173 24 5.50 1 0.83 0.00
45173 25 5.49 2 0.58 0.46
45173 26 5.46 1 0.83 0.00
45173 27 5.43 2 0.57 0.01
45173 28 5.43 2 0.58 0.06
45173 29 5.24 3 0.43 0.13
45173 3 8.05 2 0.86 0.08
45173 30 5.23 1 0.79 0.00
45173 31 5.18 2 0.55 0.08
45173 32 5.17 1 0.78 0.00
45173 33 5.11 2 0.55 0.01
45173 34 5.11 1 0.77 0.00
45173 35 5.10 2 0.54 0.03
45173 36 5.10 2 0.55 0.11
45173 37 5.07 2 0.54 0.31
45173 38 5.06 2 0.55 0.11
45173 39 5.06 1 0.77 0.00
45173 4 7.33 10 0.34 0.14

45173 40 5.05 1 0.76 0.00
45173 41 5.04 3 0.42 0.24
45173 42 5.01 1 0.76 0.00
45173 43 4.96 4 0.37 0.12
45173 44 4.95 3 0.41 0.08
45173 45 4.87 1 0.74 0.00
45173 46 4.86 1 0.74 0.00
45173 47 4.82 2 0.52 0.01
45173 48 4.80 1 0.73 0.00
45173 5 7.27 4 0.54 0.08
45173 6 7.00 4 0.52 0.09
45173 7 6.86 4 0.51 0.05
45173 8 6.85 1 1.04 0.00
45173 9 6.71 3 0.55 0.40
90201 1 5.31 3 0.82 0.07
90201 2 4.25 8 0.42 0.29
90220 1 5.09 1 0.80 0.00
90220 2 4.12 1 0.64 0.00
90220 3 3.76 1 0.59 0.00
90220 4 3.76 1 0.59 0.00
90246 1 7.91 5 0.47 0.10
90246 10 5.26 1 0.70 0.00
90246 11 5.20 1 0.69 0.00
90246 12 4.93 1 0.66 0.00
90246 13 4.89 1 0.65 0.00
90246 14 4.83 1 0.64 0.00
90246 15 4.80 1 0.64 0.00
90246 16 4.59 2 0.45 0.00
90246 17 4.55 1 0.60 0.00
90246 18 4.49 1 0.60 0.00
90246 19 4.45 2 0.43 0.07
90246 2 6.86 1 0.91 0.00
90246 20 4.45 1 0.59 0.00
90246 3 6.09 1 0.81 0.00
90246 4 5.82 1 0.78 0.00
90246 5 5.76 1 0.77 0.00
90246 6 5.73 2 0.56 0.02
90246 7 5.49 1 0.73 0.00
90246 8 5.35 1 0.71 0.00
90246 9 5.27 1 0.70 0.00
90292 1 5.47 9 0.27 0.21
90292 2 4.99 1 0.72 0.00
90292 3 4.97 1 0.72 0.00

90292 4 4.41 3 0.38 0.01
90292 5 4.24 1 0.61 0.00
90589 1 9.94 6 0.57 0.12
90589 10 4.58 2 0.51 0.17
90589 11 4.46 1 0.66 0.00
90589 12 4.43 4 0.33 0.03
90589 13 4.31 2 0.47 0.02
90589 14 4.26 2 0.47 0.05
90589 2 7.22 2 0.81 0.03
90589 3 5.87 1 0.87 0.00
90589 4 5.87 1 0.87 0.00
90589 5 5.56 1 0.83 0.00
90589 6 5.52 1 0.82 0.00
90589 7 5.45 1 0.81 0.00
90589 8 5.14 1 0.76 0.00
90589 9 4.86 1 0.72 0.00
90704 1 7.55 1 0.68 0.00
90704 10 4.84 1 0.43 0.00
90704 11 4.68 1 0.42 0.00
90704 12 4.52 2 0.34 0.34
90704 13 4.30 2 0.33 0.36
90704 14 4.22 1 0.38 0.00
90704 15 4.13 4 0.20 0.25
90704 16 4.10 2 0.31 0.26
90704 2 7.24 1 0.65 0.00
90704 3 7.12 1 0.64 0.00
90704 4 6.83 1 0.61 0.00
90704 5 6.65 1 0.60 0.00
90704 6 5.93 1 0.53 0.00
90704 7 5.85 1 0.52 0.00
90704 8 5.17 1 0.46 0.00
90704 9 4.99 1 0.45 0.00
90879 1 6.57 4 0.70 0.06
90879 10 4.62 4 0.49 0.33
90879 11 4.60 1 0.96 0.00
90879 12 4.60 1 0.96 0.00
90879 13 4.57 1 0.95 0.00
90879 14 4.49 2 0.72 0.20
90879 15 4.48 2 0.72 0.01
90879 16 4.40 1 0.91 0.00
90879 17 4.31 1 0.89 0.00
90879 18 4.30 1 0.89 0.00
90879 19 4.30 1 0.89 0.00

90879 2 5.64 3 0.69 0.20
90879 20 4.24 1 0.88 0.00
90879 3 5.29 2 0.85 0.02
90879 4 4.84 6 0.41 0.30
90879 5 4.76 2 0.77 0.17
90879 6 4.73 2 0.76 0.05
90879 7 4.71 1 0.98 0.00
90879 8 4.68 1 0.97 0.00
90879 9 4.67 1 0.97 0.00
91119 1 4.87 1 1.03 0.00
91119 2 4.42 1 0.93 0.00
91119 3 3.99 1 0.84 0.00
91119 4 3.99 1 0.84 0.00
91119 5 3.99 1 0.84 0.00
91119 6 3.80 1 0.80 0.00
91423 1 5.77 1 0.79 0.00
91423 10 4.26 1 0.58 0.00
91423 11 4.24 1 0.58 0.00
91423 2 5.75 1 0.78 0.00
91423 3 5.34 1 0.73 0.00
91423 4 5.12 1 0.70 0.00
91423 5 4.89 1 0.67 0.00
91423 6 4.79 1 0.65 0.00
91423 7 4.77 1 0.65 0.00
91423 8 4.39 1 0.60 0.00
91423 9 4.37 1 0.59 0.00

3. **Compound Membership in Each Cluster and the Citation for Each Compound**

CSP_NO CLUSTER MOLECULE COMPOUND_NAME CITATION
2966 1 1 7444 Shinbo, T.; Yamaguchi, T.; Nishimura, K.; Sugiura, M.; J. Chromatogr., 405, 145–153, 1987.
2966 1 2 104857 Wang, M.X.; Lin, S.J.; J. Org. Chem., 67, 6542–6545, 2002.
2966 1 3 98605 Youshko, M.I.; van Langen, L.M.; Sheldon, R.A.; Svedas, V.K.; Tetrahedron: Asymmetry, 15, 1933–1936, 2004.
2966 1 4 104868 Wang, M.X.; Lin, S.J.; J. Org. Chem., 67, 6542–6545, 2002.
2966 2 1 48509 Aboul–Enein, H. Y.; Serignese, V.; Biomed. Chromatogr., 11, 7–10, 1997.
2966 2 2 7928 Daicel Chem. Ind., Ltd.; Daicel Application Guide, 1989.
2966 3 1 37727 Döbler, C.; Kreuzfeld, H.–J.; Michalik, M.; Krause, H.W.; Tetrahedron: Asymmetry, 7, 117–125, 1996.
2966 3 2 44997 Houng, J. Y.; Wu, M. L.; Chen, S. T.; Chirality, 8, 418–422, 1996.
2966 4 1 7444 Shinbo, T.; Yamaguchi, T.; Nishimura, K.; Sugiura, M.; J. Chromatogr., 405, 145–153, 1987.
2966 4 2 7979 Daicel Chem. Ind., Ltd.; Daicel Application Guide, 1989.

23735 1 1 200705 Liao, Y.H.; Liu, X.L.; Wu, Z.J.; Du, X.L.; Zhang, X.M.; Yuan, W.C.; Chem. Eur. J., 18, 6679–6687, 2012.
23735 1 2 200685 Liao, Y.H.; Liu, X.L.; Wu, Z.J.; Du, X.L.; Zhang, X.M.; Yuan, W.C.; Chem. Eur. J., 18, 6679–6687, 2012.
23735 1 3 200712 Liao, Y.H.; Liu, X.L.; Wu, Z.J.; Du, X.L.; Zhang, X.M.; Yuan, W.C.; Chem. Eur. J., 18, 6679–6687, 2012.
23735 1 4 200694 Liao, Y.H.; Liu, X.L.; Wu, Z.J.; Du, X.L.; Zhang, X.M.; Yuan, W.C.; Chem. Eur. J., 18, 6679–6687, 2012.
23735 1 5 200723 Liao, Y.H.; Liu, X.L.; Wu, Z.J.; Du, X.L.; Zhang, X.M.; Yuan, W.C.; Chem. Eur. J., 18, 6679–6687, 2012.
23735 1 6 200695 Liao, Y.H.; Liu, X.L.; Wu, Z.J.; Du, X.L.; Zhang, X.M.; Yuan, W.C.; Chem. Eur. J., 18, 6679–6687, 2012.
23735 1 7 200714 Liao, Y.H.; Liu, X.L.; Wu, Z.J.; Du, X.L.; Zhang, X.M.; Yuan, W.C.; Chem. Eur. J., 18, 6679–6687, 2012.
23735 1 8 200696 Liao, Y.H.; Liu, X.L.; Wu, Z.J.; Du, X.L.; Zhang, X.M.; Yuan, W.C.; Chem. Eur. J., 18, 6679–6687, 2012.
23735 1 9 200716 Liao, Y.H.; Liu, X.L.; Wu, Z.J.; Du, X.L.; Zhang, X.M.; Yuan, W.C.; Chem. Eur. J., 18, 6679–6687, 2012.
23735 1 10 200698 Liao, Y.H.; Liu, X.L.; Wu, Z.J.; Du, X.L.; Zhang, X.M.; Yuan, W.C.; Chem. Eur. J., 18, 6679–6687, 2012.
23735 1 11 200715 Liao, Y.H.; Liu, X.L.; Wu, Z.J.; Du, X.L.; Zhang, X.M.; Yuan, W.C.; Chem. Eur. J., 18, 6679–6687, 2012.
23735 1 12 200697 Liao, Y.H.; Liu, X.L.; Wu, Z.J.; Du, X.L.; Zhang, X.M.; Yuan, W.C.; Chem. Eur. J., 18, 6679–6687, 2012.
23735 1 13 200708 Liao, Y.H.; Liu, X.L.; Wu, Z.J.; Du, X.L.; Zhang, X.M.; Yuan, W.C.; Chem. Eur. J., 18, 6679–6687, 2012.
23735 1 14 200688 Liao, Y.H.; Liu, X.L.; Wu, Z.J.; Du, X.L.; Zhang, X.M.; Yuan, W.C.; Chem. Eur. J., 18, 6679–6687, 2012.
23735 1 15 200706 Liao, Y.H.; Liu, X.L.; Wu, Z.J.; Du, X.L.; Zhang, X.M.; Yuan, W.C.; Chem. Eur. J., 18, 6679–6687, 2012.
23735 1 16 200686 Liao, Y.H.; Liu, X.L.; Wu, Z.J.; Du, X.L.; Zhang, X.M.; Yuan, W.C.; Chem. Eur. J., 18, 6679–6687, 2012.
23735 1 17 200707 Liao, Y.H.; Liu, X.L.; Wu, Z.J.; Du, X.L.; Zhang, X.M.; Yuan, W.C.; Chem. Eur. J., 18, 6679–6687, 2012.
23735 1 18 200687 Liao, Y.H.; Liu, X.L.; Wu, Z.J.; Du, X.L.; Zhang, X.M.; Yuan, W.C.; Chem. Eur. J., 18, 6679–6687, 2012.
23735 1 19 200709 Liao, Y.H.; Liu, X.L.; Wu, Z.J.; Du, X.L.; Zhang, X.M.; Yuan, W.C.; Chem. Eur. J., 18, 6679–6687, 2012.
23735 1 20 200689 Liao, Y.H.; Liu, X.L.; Wu, Z.J.; Du, X.L.; Zhang, X.M.; Yuan, W.C.; Chem. Eur. J., 18, 6679–6687, 2012.
23735 1 21 200711 Liao, Y.H.; Liu, X.L.; Wu, Z.J.; Du, X.L.; Zhang, X.M.; Yuan, W.C.; Chem. Eur. J., 18, 6679–6687, 2012.

23735 1 22 200692 Liao, Y.H.; Liu, X.L.; Wu, Z.J.; Du, X.L.; Zhang, X.M.; Yuan, W.C.; Chem. Eur. J., 18, 6679–6687, 2012.
23735 1 23 200710 Liao, Y.H.; Liu, X.L.; Wu, Z.J.; Du, X.L.; Zhang, X.M.; Yuan, W.C.; Chem. Eur. J., 18, 6679–6687, 2012.
23735 1 24 200691 Liao, Y.H.; Liu, X.L.; Wu, Z.J.; Du, X.L.; Zhang, X.M.; Yuan, W.C.; Chem. Eur. J., 18, 6679–6687, 2012.
23735 2 1 104139 Okino, T.; Hoashi, Y.; Takemoto, Y.; J. Am. Chem. Soc., 125, 12672–12673, 2003.
23735 2 2 128037 Betancort, J.M.; Sakthivel, K.; Thayumanavan, R.; Tanaka, F.; Barbas III, C.F.; Synthesis, 1509–1521, 2004.
23735 2 3 126057 Evans, D.A.; Seidel, D.; J. Am. Chem. Soc., 127, 9958–9959, 2005.
23735 2 4 158127 Wascholowski, V.; Knudsen, K.R.; Mitchell, C.E.T.; Ley, S.V.; Chem. Eur. J., 14, 6155–6165, 2008.
23735 2 5 166222 Almasi, D.; Alonso, D.A.; Gomez–Bengoa, E.; Najera, C.; J. Org. Chem. , 74, 6163–6168, 2009.
23735 2 6 158126 Wascholowski, V.; Knudsen, K.R.; Mitchell, C.E.T.; Ley, S.V.; Chem. Eur. J., 14, 6155–6165, 2008.
23735 2 7 126055 Evans, D.A.; Seidel, D.; J. Am. Chem. Soc., 127, 9958–9959, 2005.
23735 2 8 158131 Wascholowski, V.; Knudsen, K.R.; Mitchell, C.E.T.; Ley, S.V.; Chem. Eur. J., 14, 6155–6165, 2008.
23735 2 9 104143 Okino, T.; Hoashi, Y.; Takemoto, Y.; J. Am. Chem. Soc., 125, 12672–12673, 2003.
23735 2 10 158137 Wascholowski, V.; Knudsen, K.R.; Mitchell, C.E.T.; Ley, S.V.; Chem. Eur. J., 14, 6155–6165, 2008.
23735 2 11 126062 Evans, D.A.; Seidel, D.; J. Am. Chem. Soc., 127, 9958–9959, 2005.
23735 2 12 128044 Betancort, J.M.; Sakthivel, K.; Thayumanavan, R.; Tanaka, F.; Barbas III, C.F.; Synthesis, 1509–1521, 2004.
23735 2 13 126056 Evans, D.A.; Seidel, D.; J. Am. Chem. Soc., 127, 9958–9959, 2005.
23735 2 14 158134 Wascholowski, V.; Knudsen, K.R.; Mitchell, C.E.T.; Ley, S.V.; Chem. Eur. J., 14, 6155–6165, 2008.
23735 2 15 166223 Almasi, D.; Alonso, D.A.; Gomez–Bengoa, E.; Najera, C.; J. Org. Chem. , 74, 6163–6168, 2009.
23735 2 16 128043 Betancort, J.M.; Sakthivel, K.; Thayumanavan, R.; Tanaka, F.; Barbas III, C.F.; Synthesis, 1509–1521, 2004.
23735 2 17 121326 Okino, T.; Hoashi, Y.; Furukawa, T.; Xu, X.; Takemoto, Y.; J. Am. Chem. Soc., 127, 119–125, 2005.
23735 2 18 128038 Betancort, J.M.; Sakthivel, K.; Thayumanavan, R.; Tanaka, F.; Barbas III, C.F.; Synthesis, 1509–1521, 2004.
23735 2 19 114061 Annamalai, V.; Dimauro, E.F.; Carroll, P.J.; Kozlowski, M.C.; J. Org. Chem., 68, 1973–1981, 2003.
23735 2 20 128036 Betancort, J.M.; Sakthivel, K.; Thayumanavan, R.; Tanaka, F.; Barbas III, C.F.; Synthesis, 1509–1521, 2004.

23735 3 1 49045 Chankvetadze, B.; Chankvetadze, L.; Sidamonidze, S.; Yashima, E.; Okamoto, Y.; J. Pharm. Biomed. Anal., 13, 695–699, 1995.

23735 3 2 136779 Ghanem, A.; Al–Humaidi, E.; Chirality, 19, 477–484, 2007.

23735 4 1 49043 Chankvetadze, B.; Chankvetadze, L.; Sidamonidze, S.; Yashima, E.; Okamoto, Y.; J. Pharm. Biomed. Anal., 13, 695–699, 1995.

23735 4 2 136814 Ghanem, A.; Al–Humaidi, E.; Chirality, 19, 477–484, 2007.

23735 5 1 206442 Hashimoto, T.; Omote, M.; Maruoka, K.; Angew. Chem., Int. Ed., 50, 8952–8955, 2011.

23735 5 2 206433 Hashimoto, T.; Omote, M.; Maruoka, K.; Angew. Chem., Int. Ed., 50, 8952–8955, 2011.

23735 5 3 206444 Hashimoto, T.; Omote, M.; Maruoka, K.; Angew. Chem., Int. Ed., 50, 8952–8955, 2011.

23735 5 4 206436 Hashimoto, T.; Omote, M.; Maruoka, K.; Angew. Chem., Int. Ed., 50, 8952–8955, 2011.

23735 5 5 206438 Hashimoto, T.; Omote, M.; Maruoka, K.; Angew. Chem., Int. Ed., 50, 8952–8955, 2011.

23735 5 6 206419 Hashimoto, T.; Omote, M.; Maruoka, K.; Angew. Chem., Int. Ed., 50, 8952–8955, 2011.

23735 5 7 206443 Hashimoto, T.; Omote, M.; Maruoka, K.; Angew. Chem., Int. Ed., 50, 8952–8955, 2011.

23735 5 8 206435 Hashimoto, T.; Omote, M.; Maruoka, K.; Angew. Chem., Int. Ed., 50, 8952–8955, 2011.

23735 5 9 206439 Hashimoto, T.; Omote, M.; Maruoka, K.; Angew. Chem., Int. Ed., 50, 8952–8955, 2011.

23735 5 10 206430 Hashimoto, T.; Omote, M.; Maruoka, K.; Angew. Chem., Int. Ed., 50, 8952–8955, 2011.

23735 5 11 206441 Hashimoto, T.; Omote, M.; Maruoka, K.; Angew. Chem., Int. Ed., 50, 8952–8955, 2011.

23735 5 12 206432 Hashimoto, T.; Omote, M.; Maruoka, K.; Angew. Chem., Int. Ed., 50, 8952–8955, 2011.

23735 6 1 126051 Evans, D.A.; Seidel, D.; J. Am. Chem. Soc., 127, 9958–9959, 2005.

23735 6 2 164736 Zhu, Q.; Huang, H.; Shi, D.; Shen, Z.; Xia, C.; Org. Lett. , 11, 4536–4539, 2009.

23735 7 1 202955 Streuff, J.; Feurer, M.; Bichovski, P.; Frey, G.; Gellrich, U.; Angew. Chem., Int. Ed., 51, 8661–8664, 2012.

23735 7 2 205423 Ishikawa, S.; Hamada, T.; Manabe, K.; Kobayashi, S.; J. Am. Chem. Soc, 126, 12236–12237, 2004.

23735 8 1 49043 Chankvetadze, B.; Chankvetadze, L.; Sidamonidze, S.; Yashima, E.; Okamoto, Y.; J. Pharm. Biomed. Anal., 13, 695–699, 1995.

23735 8 2 136807 Ghanem, A.; Al–Humaidi, E.; Chirality, 19, 477–484, 2007.

23735 9 1 49045 Chankvetadze, B.; Chankvetadze, L.; Sidamonidze, S.; Yashima, E.; Okamoto, Y.; J. Pharm. Biomed. Anal., 13, 695–699, 1995.

23735 9 2 136793 Ghanem, A.; Al–Humaidi, E.; Chirality, 19, 477–484, 2007.

23735 10 1 106389 Ye, Y.K.; Stringham, R.W.; Wirth, M.J.; J. Chromatogr., A, 1057, 75–82, 2004.
23735 10 2 106392 Ye, Y.K.; Stringham, R.W.; Wirth, M.J.; J. Chromatogr., A, 1057, 75–82, 2004.
23735 11 1 106389 Ye, Y.K.; Stringham, R.W.; Wirth, M.J.; J. Chromatogr., A, 1057, 75–82, 2004.
23735 11 2 106386 Ye, Y.K.; Stringham, R.W.; Wirth, M.J.; J. Chromatogr., A, 1057, 75–82, 2004.
23735 12 1 201588 Lu, S.M.; Wang, Y.Q.; Han, X.W.; Zhou, Y.G.; Angew. Chem., Int. Ed., 45, 2260–2263, 2006.
23735 12 2 211297 Iimuro, A.; Yamaji, K.; Kandula, S.; Nagano, T.; Kita, Y.; Mashima, K.; Angew. Chem., Int. Ed., 52, 2046–2050, 2013.
23735 13 1 216479 Fernandes, C. ; Brandao, P. ; Santos, A. ; Tiritan, M. E. ; Afonso, C. ; Cass, Q. B. ; Pinto, M. M.; J. Chromatogr., A, 1269, 143–153, 2012.
23735 13 2 216502 Fernandes, C. ; Brandao, P. ; Santos, A. ; Tiritan, M. E. ; Afonso, C. ; Cass, Q. B. ; Pinto, M. M.; J. Chromatogr., A, 1269, 143–153, 2012.
23735 14 1 166224 Almasi, D.; Alonso, D.A.; Gomez–Bengoa, E.; Najera, C.; J. Org. Chem. , 74, 6163–6168, 2009.
23735 14 2 181297 McGarraugh, P.G.; Brenner, S.E.; Tetrahedron, 65, 449–455, 2009.
23735 15 1 216463 Fernandes, C. ; Brandao, P. ; Santos, A. ; Tiritan, M. E. ; Afonso, C. ; Cass, Q. B. ; Pinto, M. M.; J. Chromatogr., A, 1269, 143–153, 2012.
23735 15 2 216502 Fernandes, C. ; Brandao, P. ; Santos, A. ; Tiritan, M. E. ; Afonso, C. ; Cass, Q. B. ; Pinto, M. M.; J. Chromatogr., A, 1269, 143–153, 2012.
23735 16 1 49045 Chankvetadze, B.; Chankvetadze, L.; Sidamonidze, S.; Yashima, E.; Okamoto, Y.; J. Pharm. Biomed. Anal., 13, 695–699, 1995.
23735 16 2 106221 Li, T.; US Patent Application 2004226889, 2004.
23735 17 1 202955 Streuff, J.; Feurer, M.; Bichovski, P.; Frey, G.; Gellrich, U.; Angew. Chem., Int. Ed., 51, 8661–8664, 2012.
23735 17 2 101714 Nakamura, H.; Ishihara, K.; Yamamoto, H.; J. Org. Chem., 67, 5124–5137, 2002.
23735 18 1 49043 Chankvetadze, B.; Chankvetadze, L.; Sidamonidze, S.; Yashima, E.; Okamoto, Y.; J. Pharm. Biomed. Anal., 13, 695–699, 1995.
23735 18 2 136786 Ghanem, A.; Al–Humaidi, E.; Chirality, 19, 477–484, 2007.
23735 19 1 115853 CHIRAL TECHNOLOGIES EUROPE; Application Guide Software, 2006.
23735 19 2 92149 Chankvetadze, B.; Kartozia, I.; Yamamoto, C.; Okamoto, Y.; J. Pharm. Biomed. Anal., 27, 467–478, 2002.
23735 20 1 49045 Chankvetadze, B.; Chankvetadze, L.; Sidamonidze, S.; Yashima, E.; Okamoto, Y.; J. Pharm. Biomed. Anal., 13, 695–699, 1995.
23735 20 2 136758 Ghanem, A.; Al–Humaidi, E.; Chirality, 19, 477–484, 2007.
23735 21 1 206426 Hashimoto, T.; Omote, M.; Maruoka, K.; Angew. Chem., Int. Ed., 50, 8952–8955, 2011.
23735 21 2 206430 Hashimoto, T.; Omote, M.; Maruoka, K.; Angew. Chem., Int. Ed., 50, 8952–8955, 2011.

23735 22 1 166223 Almasi, D.; Alonso, D.A.; Gomez–Bengoa, E.; Najera, C.; J. Org. Chem. , 74, 6163–6168, 2009.

23735 22 2 162728 Kwon, B.K.; Kim, S.M.; Kim, D.Y.; J. Fluorine Chem., 130, 759–761, 2009.

23735 23 1 206426 Hashimoto, T.; Omote, M.; Maruoka, K.; Angew. Chem., Int. Ed., 50, 8952–8955, 2011.

23735 23 2 206429 Hashimoto, T.; Omote, M.; Maruoka, K.; Angew. Chem., Int. Ed., 50, 8952–8955, 2011.

23735 24 1 93217 Qiu, Y.L.; Geiser, F.; Kira, T.; Gullen, E.; Cheng, Y.C.; Ptak, R.G.; Breitenbach, J.M.; Zemlicka, J.; Antiviral Chem. Chemother., 11, 191–202, 2000.

23735 24 2 93218 Qiu, Y.L.; Geiser, F.; Kira, T.; Gullen, E.; Cheng, Y.C.; Ptak, R.G.; Breitenbach, J.M.; Zemlicka, J.; Antiviral Chem. Chemother., 11, 191–202, 2000.

23735 25 1 88863 Cass, Q.B.; Batigalhia, F.; HPLC'2002 Montreal, 2002.

23735 25 2 97616 Cass, Q.B.; Batigalhia, F.; J. Chromatogr., A, 987, 445–452, 2003.

23735 26 1 48059 Kim, B. H.; Lee, W.; Ok, J. H.; International Symposium on Chiral Discrimination (9th), Nagoya, 1997.

23735 26 2 4564 Okamoto, Y.; Kaida, Y.; Aburatani, R.; Hatada, K.; J. Chromatogr., 477, 367–376, 1989.

23735 26 3 48071 Kim, B. H.; Lee, W.; Ok, J. H.; International Symposium on Chiral Discrimination (9th), Nagoya, 1997.

23735 26 4 4676 Okamoto, Y.; Kaida, Y.; Aburatani, R.; Hatada, K.; J. Chromatogr., 477, 367–376, 1989.

23735 27 1 200914 Huang, D.; Xu, F.X.; Lin, X.F.; Wang, Y.G.; Chem. Eur. J., 18, 3148–3152, 2012.

23735 27 2 200913 Huang, D.; Xu, F.X.; Lin, X.F.; Wang, Y.G.; Chem. Eur. J., 18, 3148–3152, 2012.

23735 27 3 206426 Hashimoto, T.; Omote, M.; Maruoka, K.; Angew. Chem., Int. Ed., 50, 8952–8955, 2011.

23735 27 4 206419 Hashimoto, T.; Omote, M.; Maruoka, K.; Angew. Chem., Int. Ed., 50, 8952–8955, 2011.

23735 28 1 200914 Huang, D.; Xu, F.X.; Lin, X.F.; Wang, Y.G.; Chem. Eur. J., 18, 3148–3152, 2012.

23735 28 2 200900 Huang, D.; Xu, F.X.; Lin, X.F.; Wang, Y.G.; Chem. Eur. J., 18, 3148–3152, 2012.

23735 28 3 206426 Hashimoto, T.; Omote, M.; Maruoka, K.; Angew. Chem., Int. Ed., 50, 8952–8955, 2011.

23735 28 4 206424 Hashimoto, T.; Omote, M.; Maruoka, K.; Angew. Chem., Int. Ed., 50, 8952–8955, 2011.

23735 29 1 84689 Aboul–Enein, H.Y.; Ali, I.; Gubitz, G.; Simons, C.; Nicholls, P.J.; Chirality, 12, 727–733, 2000.

23735 29 2 26214 Pepper, C.; Smith, H.J.; Barrell, K.J.; Nicholls, P.J.; Hewlins, M.J.E.; Chirality, 6, 400–404, 1994.

23735 29 3 84685 Aboul–Enein, H.Y.; Ali, I.; Gubitz, G.; Simons, C.; Nicholls, P.J.; Chirality, 12, 727–733, 2000.

23735 29 4 80260 Botta, M.; Corelli, F.; Gasparrini, F.; Messina, F.; Mugnaini, C.; J. Org. Chem., 65, 4736–4739, 2000.

23735 30 1 93220 Qiu, Y.L.; Geiser, F.; Kira, T.; Gullen, E.; Cheng, Y.C.; Ptak, R.G.; Breitenbach, J.M.; Zemlicka, J.; Antiviral Chem. Chemother., 11, 191–202, 2000.

23735 30 2 93218 Qiu, Y.L.; Geiser, F.; Kira, T.; Gullen, E.; Cheng, Y.C.; Ptak, R.G.; Breitenbach, J.M.; Zemlicka, J.; Antiviral Chem. Chemother., 11, 191–202, 2000.

23735 31 1 200712 Liao, Y.H.; Liu, X.L.; Wu, Z.J.; Du, X.L.; Zhang, X.M.; Yuan, W.C.; Chem. Eur. J., 18, 6679–6687, 2012.

23735 31 2 200718 Liao, Y.H.; Liu, X.L.; Wu, Z.J.; Du, X.L.; Zhang, X.M.; Yuan, W.C.; Chem. Eur. J., 18, 6679–6687, 2012.

23735 32 1 169508 Flugge, S.; Universität Dortmund, Thesis, 2009.

23735 32 2 169507 Flugge, S.; Universität Dortmund, Thesis, 2009.

23735 33 1 206426 Hashimoto, T.; Omote, M.; Maruoka, K.; Angew. Chem., Int. Ed., 50, 8952–8955, 2011.

23735 33 2 206432 Hashimoto, T.; Omote, M.; Maruoka, K.; Angew. Chem., Int. Ed., 50, 8952–8955, 2011.

23735 34 1 146173 Cullen, S.C.; Rovis, T.; Org. Lett., 10, 3141–3144, 2008.

23735 34 2 146171 Cullen, S.C.; Rovis, T.; Org. Lett., 10, 3141–3144, 2008.

23735 35 1 95840 Ye, Y.K.; Lord, B.; Stringham, R.W.; J. Chromatogr., A, 945, 139–146, 2002.

23735 35 2 106617 Ye, Y.K.; Lynam, K.G.; Stringham, R.W.; J. Chromatogr., A, 1041, 211–217, 2004.

23735 35 3 95836 Ye, Y.K.; Lord, B.; Stringham, R.W.; J. Chromatogr., A, 945, 139–146, 2002.

23735 35 4 106631 Ye, Y.K.; Lynam, K.G.; Stringham, R.W.; J. Chromatogr., A, 1041, 211–217, 2004.

23735 36 1 126056 Evans, D.A.; Seidel, D.; J. Am. Chem. Soc., 127, 9958–9959, 2005.

23735 36 2 126062 Evans, D.A.; Seidel, D.; J. Am. Chem. Soc., 127, 9958–9959, 2005.

23735 37 1 206426 Hashimoto, T.; Omote, M.; Maruoka, K.; Angew. Chem., Int. Ed., 50, 8952–8955, 2011.

23735 37 2 206421 Hashimoto, T.; Omote, M.; Maruoka, K.; Angew. Chem., Int. Ed., 50, 8952–8955, 2011.

23735 38 1 126058 Evans, D.A.; Seidel, D.; J. Am. Chem. Soc., 127, 9958–9959, 2005.

23735 38 2 162728 Kwon, B.K.; Kim, S.M.; Kim, D.Y.; J. Fluorine Chem., 130, 759–761, 2009.

23735 39 1 126056 Evans, D.A.; Seidel, D.; J. Am. Chem. Soc., 127, 9958–9959, 2005.

23735 39 2 162728 Kwon, B.K.; Kim, S.M.; Kim, D.Y.; J. Fluorine Chem., 130, 759–761, 2009.

23735 40 1 189350 Ioannidis, S.; Lamb, M.L.; Wang, T.; Almeida, L.; Block, M.H.; Davies, A.M.; Peng, B.; Su, M.; Zhang, H.–J.; Hoffmann, E.; Rivard, C.; Green, I.; Howard, T.; Pollard, H.; Read, J.; Alimzhanov, M.; Beb|ernitz, G.; Bell, K.; Ye, M.; Huszar, D.; Zinda, M.; J. Med. Chem, 54, 262–276, 2011.

23735 40 2 176561 Feng, X.; Guan, H.; Ioannidis, S.; Peng, B.; Su, M.; Wang, T.; Zhang, H.–J.; Kan, Y.; Wang, B.; ; US Patent Application 20100160325, 2010.

23735 41 1 200712 Liao, Y.H.; Liu, X.L.; Wu, Z.J.; Du, X.L.; Zhang, X.M.; Yuan, W.C.; Chem. Eur. J., 18, 6679–6687, 2012.

23735 41 2 200721 Liao, Y.H.; Liu, X.L.; Wu, Z.J.; Du, X.L.; Zhang, X.M.; Yuan, W.C.; Chem. Eur. J., 18, 6679–6687, 2012.

23735 42 1 210711 Wu, Z.; Perez, M.; Scalone, M.; Ayad, T.; Ratovelomanana–Vidal, V.; Angew. Chem., Int. Ed., 52, 4925–4928, 2013.

23735 42 2 210698 Wu, Z.; Perez, M.; Scalone, M.; Ayad, T.; Ratovelomanana–Vidal, V.; Angew. Chem., Int. Ed., 52, 4925–4928, 2013.

23735 43 1 81748 Chankvetadze, B.; Yamamoto, C.; Okamoto, Y.; Chem. Lett., 1176–1177, 2000.

23735 43 2 26955 Matlin, S.A.; Tiritan, M.E.; Crawford, A.J.; Cass, Q.B.; Boyd, D.R.; Chirality, 6, 135–140, 1994.

23735 44 1 95840 Ye, Y.K.; Lord, B.; Stringham, R.W.; J. Chromatogr., A, 945, 139–146, 2002.

23735 44 2 106619 Ye, Y.K.; Lynam, K.G.; Stringham, R.W.; J. Chromatogr., A, 1041, 211–217, 2004.

23735 44 3 95836 Ye, Y.K.; Lord, B.; Stringham, R.W.; J. Chromatogr., A, 945, 139–146, 2002.

23735 44 4 106633 Ye, Y.K.; Lynam, K.G.; Stringham, R.W.; J. Chromatogr., A, 1041, 211–217, 2004.

23735 45 1 210712 Wu, Z.; Perez, M.; Scalone, M.; Ayad, T.; Ratovelomanana–Vidal, V.; Angew. Chem., Int. Ed., 52, 4925–4928, 2013.

23735 45 2 210701 Wu, Z.; Perez, M.; Scalone, M.; Ayad, T.; Ratovelomanana–Vidal, V.; Angew. Chem., Int. Ed., 52, 4925–4928, 2013.

23735 45 3 210710 Wu, Z.; Perez, M.; Scalone, M.; Ayad, T.; Ratovelomanana–Vidal, V.; Angew. Chem., Int. Ed., 52, 4925–4928, 2013.

23735 45 4 210695 Wu, Z.; Perez, M.; Scalone, M.; Ayad, T.; Ratovelomanana–Vidal, V.; Angew. Chem., Int. Ed., 52, 4925–4928, 2013.

23735 45 5 210713 Wu, Z.; Perez, M.; Scalone, M.; Ayad, T.; Ratovelomanana–Vidal, V.; Angew. Chem., Int. Ed., 52, 4925–4928, 2013.

23735 45 6 210702 Wu, Z.; Perez, M.; Scalone, M.; Ayad, T.; Ratovelomanana–Vidal, V.; Angew. Chem., Int. Ed., 52, 4925–4928, 2013.

23735 45 7 210711 Wu, Z.; Perez, M.; Scalone, M.; Ayad, T.; Ratovelomanana–Vidal, V.; Angew. Chem., Int. Ed., 52, 4925–4928, 2013.

23735 45 8 210696 Wu, Z.; Perez, M.; Scalone, M.; Ayad, T.; Ratovelomanana–Vidal, V.; Angew. Chem., Int. Ed., 52, 4925–4928, 2013.

23735 45 9 210706 Wu, Z.; Perez, M.; Scalone, M.; Ayad, T.; Ratovelomanana–Vidal, V.; Angew. Chem., Int. Ed., 52, 4925–4928, 2013.

23735 45 10 210709 Wu, Z.; Perez, M.; Scalone, M.; Ayad, T.; Ratovelomanana–Vidal, V.; Angew. Chem., Int. Ed., 52, 4925–4928, 2013.

23735 46 1 95840 Ye, Y.K.; Lord, B.; Stringham, R.W.; J. Chromatogr., A, 945, 139–146, 2002.

23735 46 2 85528 Ye, Y.K.; Stringham, R.; J. Chromatogr., A, 927, 53–60, 2001.

23735 46 3 95836 Ye, Y.K.; Lord, B.; Stringham, R.W.; J. Chromatogr., A, 945, 139–146, 2002.

23735 46 4 85624 Ye, Y.K.; Stringham, R.; J. Chromatogr., A, 927, 53–60, 2001.

23735 47 1 197558 Andrus, M.B.; Christiansen, M.A.; Hicken, E.J.; Gainer, M.J.; Bedke, D.K.; Harper, K.C.; Mikkelson, S.R.; Dodson, D.S.; Harris, D.T.; Org. Lett., 9, 4865–4868, 2007.

23735 47 2 197567 Andrus, M.B.; Christiansen, M.A.; Hicken, E.J.; Gainer, M.J.; Bedke, D.K.; Harper, K.C.; Mikkelson, S.R.; Dodson, D.S.; Harris, D.T.; Org. Lett., 9, 4865–4868, 2007.

23735 47 3 197564 Andrus, M.B.; Christiansen, M.A.; Hicken, E.J.; Gainer, M.J.; Bedke, D.K.; Harper, K.C.; Mikkelson, S.R.; Dodson, D.S.; Harris, D.T.; Org. Lett., 9, 4865–4868, 2007.

23735 47 4 197568 Andrus, M.B.; Christiansen, M.A.; Hicken, E.J.; Gainer, M.J.; Bedke, D.K.; Harper, K.C.; Mikkelson, S.R.; Dodson, D.S.; Harris, D.T.; Org. Lett., 9, 4865–4868, 2007.

23735 48 1 115000 Lipka, E.; Vaccher, M.P.; Vaccher, C.; Len, C.; Bioorg. Med. Chem. Lett., 15, 501–504, 2005.

23735 48 2 117918 Momiyama, N.; Yamamoto, H.; J. Am. Chem. Soc., 125, 6038–6039, 2003.

23735 49 1 206426 Hashimoto, T.; Omote, M.; Maruoka, K.; Angew. Chem., Int. Ed., 50, 8952–8955, 2011.

23735 49 2 206428 Hashimoto, T.; Omote, M.; Maruoka, K.; Angew. Chem., Int. Ed., 50, 8952–8955, 2011.

23735 50 1 216493 Fernandes, C. ; Brandao, P. ; Santos, A. ; Tiritan, M. E. ; Afonso, C. ; Cass, Q. B. ; Pinto, M. M.; J. Chromatogr., A, 1269, 143–153, 2012.

23735 50 2 216502 Fernandes, C. ; Brandao, P. ; Santos, A. ; Tiritan, M. E. ; Afonso, C. ; Cass, Q. B. ; Pinto, M. M.; J. Chromatogr., A, 1269, 143–153, 2012.

23735 51 1 143387 Minato, D.; Arimoto, H.; Nagasue, Y.; Demizu, Y.; Onomura, O.; Tetrahedron, 64, 6675–6683, 2008.

23735 51 2 76324 Alphand, V.; Furstoss, R.; Pedragosa–Moreau, S.; Roberts, S.M.; Willetts, A.J.; J. Chem. Soc., Perkin Trans. 1, 15, 1867–1872, 1996.

23735 51 3 202955 Streuff, J.; Feurer, M.; Bichovski, P.; Frey, G.; Gellrich, U.; Angew. Chem., Int. Ed., 51, 8661–8664, 2012.

23735 51 4 1782 Okamoto, Y.; Aburatani, R.; Fukumoto, T.; Hatada, K.; Chem. Lett., 1857–1860, 1987.

23735 52 1 200524 Lu, Z.J.; Xie, C.; Zhou, W.; Duan, Z.Q.; Han, J.L.; Pan, Y.; Chin. J. Chem., 30, 2333–2337, 2012.

23735 52 2 200523 Lu, Z.J.; Xie, C.; Zhou, W.; Duan, Z.Q.; Han, J.L.; Pan, Y.; Chin. J. Chem., 30, 2333–2337, 2012.

23735 53 1 210710 Wu, Z.; Perez, M.; Scalone, M.; Ayad, T.; Ratovelomanana–Vidal, V.; Angew. Chem., Int. Ed., 52, 4925–4928, 2013.

23735 53 2 210688 Wu, Z.; Perez, M.; Scalone, M.; Ayad, T.; Ratovelomanana–Vidal, V.; Angew. Chem., Int. Ed., 52, 4925–4928, 2013.

23735 54 1 48071 Kim, B. H.; Lee, W.; Ok, J. H.; International Symposium on Chiral Discrimination (9th), Nagoya, 1997.

23735 54 2 78063 Tachibana, K.; Ohnishi, A.; J. Chromatogr., A, 906, 127–154, 2001.

23735 55 1 200712 Liao, Y.H.; Liu, X.L.; Wu, Z.J.; Du, X.L.; Zhang, X.M.; Yuan, W.C.; Chem. Eur. J., 18, 6679–6687, 2012.

23735 55 2 200720 Liao, Y.H.; Liu, X.L.; Wu, Z.J.; Du, X.L.; Zhang, X.M.; Yuan, W.C.; Chem. Eur. J., 18, 6679–6687, 2012.

23735 56 1 48095 Kim, B. H.; Lee, W.; Ok, J. H.; International Symposium on Chiral Discrimination (9th), Nagoya, 1997.

23735 56 2 4678 Okamoto, Y.; Kaida, Y.; Aburatani, R.; Hatada, K.; J. Chromatogr., 477, 367–376, 1989.

23735 56 3 78063 Tachibana, K.; Ohnishi, A.; J. Chromatogr., A, 906, 127–154, 2001.

23735 56 4 4682 Okamoto, Y.; Kaida, Y.; Aburatani, R.; Hatada, K.; J. Chromatogr., 477, 367–376, 1989.

23735 57 1 88863 Cass, Q.B.; Batigalhia, F.; HPLC'2002 Montreal, 2002.

23735 57 2 97706 Cass, Q.B.; Batigalhia, F.; J. Chromatogr., A, 987, 445–452, 2003.

23735 58 1 104139 Okino, T.; Hoashi, Y.; Takemoto, Y.; J. Am. Chem. Soc., 125, 12672–12673, 2003.

23735 58 2 114061 Annamalai, V.; Dimauro, E.F.; Carroll, P.J.; Kozlowski, M.C.; J. Org. Chem., 68, 1973–1981, 2003.

23735 58 3 166222 Almasi, D.; Alonso, D.A.; Gomez–Bengoa, E.; Najera, C.; J. Org. Chem. , 74, 6163–6168, 2009.

23735 58 4 123961 Terada, M.; Ube, H.; Yaguchi, Y.; J. Am. Chem. Soc., 128, 1454–1455, 2006.

23735 58 5 121348 Okino, T.; Hoashi, Y.; Furukawa, T.; Xu, X.; Takemoto, Y.; J. Am. Chem. Soc., 127, 119–125, 2005.

23735 58 6 116281 Deng, L.; Li, H.; US Patent Application 20060014956, 2006.

23735 58 7 126057 Evans, D.A.; Seidel, D.; J. Am. Chem. Soc., 127, 9958–9959, 2005.

23735 58 8 123962 Terada, M.; Ube, H.; Yaguchi, Y.; J. Am. Chem. Soc., 128, 1454–1455, 2006.

23735 58 9 104143 Okino, T.; Hoashi, Y.; Takemoto, Y.; J. Am. Chem. Soc., 125, 12672–12673, 2003.

23735 58 10 116292 Deng, L.; Li, H.; US Patent Application 20060014956, 2006.

23735 58 11 126058 Evans, D.A.; Seidel, D.; J. Am. Chem. Soc., 127, 9958–9959, 2005.

23735 58 12 165840 Jiang, X.; Zhang, Y.; Liu, X.; Zhang, G.; Lai, L.; Wu, L.; Zhang, J.; Wang, R.; J. Org. Chem. , 74, 5562–5567, 2009.

23735 58 13 126056 Evans, D.A.; Seidel, D.; J. Am. Chem. Soc., 127, 9958–9959, 2005.

23735 58 14 116286 Deng, L.; Li, H.; US Patent Application 20060014956, 2006.

23735 58 15 128037 Betancort, J.M.; Sakthivel, K.; Thayumanavan, R.; Tanaka, F.; Barbas III, C.F.; Synthesis, 1509–1521, 2004.

23735 58 16 128036 Betancort, J.M.; Sakthivel, K.; Thayumanavan, R.; Tanaka, F.; Barbas III, C.F.; Synthesis, 1509–1521, 2004.

23735 58 17 200977 Brandau, S.; Landa, A.; Franzen, J.; Marigo, M.; Jorgensen, K.A.; Angew. Chem., Int. Ed., 45, 4305–4309, 2006.

23735 58 18 200966 Rueping, M.; Sunden, H.; Sugiono, E.; Chem. Eur. J., 18, 3649–3653, 2012.

23735 59 1 78063 Tachibana, K.; Ohnishi, A.; J. Chromatogr., A, 906, 127–154, 2001.

23735 59 2 4674 Okamoto, Y.; Kaida, Y.; Aburatani, R.; Hatada, K.; J. Chromatogr., 477, 367–376, 1989.

23735 59 3 48095 Kim, B. H.; Lee, W.; Ok, J. H.; International Symposium on Chiral Discrimination (9th), Nagoya, 1997.

23735 59 4 4676 Okamoto, Y.; Kaida, Y.; Aburatani, R.; Hatada, K.; J. Chromatogr., 477, 367–376, 1989.

23735 60 1 95834 Ye, Y.K.; Lord, B.; Stringham, R.W.; J. Chromatogr., A, 945, 139–146, 2002.

23735 60 2 105755 Escoubet, S.; Gastaldi, S.; Vanthuyne, N.; CHIRBASE Project, 2003.

23735 60 3 95836 Ye, Y.K.; Lord, B.; Stringham, R.W.; J. Chromatogr., A, 945, 139–146, 2002.

23735 60 4 95838 Ye, Y.K.; Lord, B.; Stringham, R.W.; J. Chromatogr., A, 945, 139–146, 2002.

23735 60 5 95840 Ye, Y.K.; Lord, B.; Stringham, R.W.; J. Chromatogr., A, 945, 139–146, 2002.

23735 60 6 106615 Ye, Y.K.; Lynam, K.G.; Stringham, R.W.; J. Chromatogr., A, 1041, 211–217, 2004.

23735 60 7 67546 Denmark, S.E.; Winter, S.B.D.; Synlett, 1087–1089, 1997.

23735 60 8 181530 Yuan, W.–C.; Cun, L.–F.; Mi, A.–Q.; Jiang, Y.–Z.; Gong, L.–Z.; Tetrahedron, 65, 4130–4141, 2009.

23735 61 1 143703 Jiang, J.; Yu, J.; Sun, X–X.; Rao, Q–Q.; Gong, L–Z.; Angew. Chem., Int. Ed., 47, 2458–2462, 2008.

23735 61 2 143719 Jiang, J.; Yu, J.; Sun, X–X.; Rao, Q–Q.; Gong, L–Z.; Angew. Chem., Int. Ed., 47, 2458–2462, 2008.

23735 61 3 143710 Jiang, J.; Yu, J.; Sun, X–X.; Rao, Q–Q.; Gong, L–Z.; Angew. Chem., Int. Ed., 47, 2458–2462, 2008.

23735 61 4 143721 Jiang, J.; Yu, J.; Sun, X–X.; Rao, Q–Q.; Gong, L–Z.; Angew. Chem., Int. Ed., 47, 2458–2462, 2008.

23735 61 5 143704 Jiang, J.; Yu, J.; Sun, X–X.; Rao, Q–Q.; Gong, L–Z.; Angew. Chem., Int. Ed., 47, 2458–2462, 2008.

23735 61 6 143720 Jiang, J.; Yu, J.; Sun, X–X.; Rao, Q–Q.; Gong, L–Z.; Angew. Chem., Int. Ed., 47, 2458–2462, 2008.

23735 61 7 143713 Jiang, J.; Yu, J.; Sun, X–X.; Rao, Q–Q.; Gong, L–Z.; Angew. Chem., Int. Ed., 47, 2458–2462, 2008.

23735 61 8 143722 Jiang, J.; Yu, J.; Sun, X–X.; Rao, Q–Q.; Gong, L–Z.; Angew. Chem., Int. Ed., 47, 2458–2462, 2008.

23735 62 1 85608 Ye, Y.K.; Stringham, R.; J. Chromatogr., A, 927, 53–60, 2001.

23735 62 2 119568 Glueck, S.M.; Pirker, M.; Nestl, B.M.; Ueberbacher, B.T.; Larissegger–Schnell, B.; Csar, K.; Hauer, B.; Stuermer, R.; Kroutil, W.; Faber, K.; J. Org. Chem., 70, 4028–4032, 2005.

23735 62 3 85640 Ye, Y.K.; Stringham, R.; J. Chromatogr., A, 927, 53–60, 2001.

23735 62 4 119570 Glueck, S.M.; Pirker, M.; Nestl, B.M.; Ueberbacher, B.T.; Larissegger–Schnell, B.; Csar, K.; Hauer, B.; Stuermer, R.; Kroutil, W.; Faber, K.; J. Org. Chem., 70, 4028–4032, 2005.

23735 62 5 85528 Ye, Y.K.; Stringham, R.; J. Chromatogr., A, 927, 53–60, 2001.

23735 62 6 119571 Glueck, S.M.; Pirker, M.; Nestl, B.M.; Ueberbacher, B.T.; Larissegger–Schnell, B.; Csar, K.; Hauer, B.; Stuermer, R.; Kroutil, W.; Faber, K.; J. Org. Chem., 70, 4028–4032, 2005.

23735 62 7 85624 Ye, Y.K.; Stringham, R.; J. Chromatogr., A, 927, 53–60, 2001.

23735 62 8 119569 Glueck, S.M.; Pirker, M.; Nestl, B.M.; Ueberbacher, B.T.; Larissegger–Schnell, B.; Csar, K.; Hauer, B.; Stuermer, R.; Kroutil, W.; Faber, K.; J. Org. Chem., 70, 4028–4032, 2005.

23735 62 9 85512 Ye, Y.K.; Stringham, R.; J. Chromatogr., A, 927, 53–60, 2001.

23735 62 10 66799 Booth, T.D.; Azzaoui, K.; Wainer, I.W.; Anal. Chem., 69, 3879–3883, 1997.

23735 63 1 126051 Evans, D.A.; Seidel, D.; J. Am. Chem. Soc., 127, 9958–9959, 2005.

23735 63 2 164732 Zhu, Q.; Huang, H.; Shi, D.; Shen, Z.; Xia, C.; Org. Lett. , 11, 4536–4539, 2009.
23735 64 1 4676 Okamoto, Y.; Kaida, Y.; Aburatani, R.; Hatada, K.; J. Chromatogr., 477, 367–376, 1989.
23735 64 2 4674 Okamoto, Y.; Kaida, Y.; Aburatani, R.; Hatada, K.; J. Chromatogr., 477, 367–376, 1989.
23735 64 3 48095 Kim, B. H.; Lee, W.; Ok, J. H.; International Symposium on Chiral Discrimination (9th), Nagoya, 1997.
23735 64 4 78063 Tachibana, K.; Ohnishi, A.; J. Chromatogr., A, 906, 127–154, 2001.
23735 65 1 142694 Hasegawa, Y.; Watanabe, M.; Gridnev, I.D.; Takao Ikariya, T.; J. Am. Chem. Soc., 130, 2158–2159, 2008.
23735 65 2 208360 Hasegawa, Y.; Gridnev, I.D.; Ikariya, T.; Angew. Chem., Int. Ed., 49, 8157–8160, 2010.
23735 65 3 142698 Hasegawa, Y.; Watanabe, M.; Gridnev, I.D.; Takao Ikariya, T.; J. Am. Chem. Soc., 130, 2158–2159, 2008.
23735 65 4 208363 Hasegawa, Y.; Gridnev, I.D.; Ikariya, T.; Angew. Chem., Int. Ed., 49, 8157–8160, 2010.
23735 65 5 142699 Hasegawa, Y.; Watanabe, M.; Gridnev, I.D.; Takao Ikariya, T.; J. Am. Chem. Soc., 130, 2158–2159, 2008.
23735 65 6 208362 Hasegawa, Y.; Gridnev, I.D.; Ikariya, T.; Angew. Chem., Int. Ed., 49, 8157–8160, 2010.
23735 65 7 142697 Hasegawa, Y.; Watanabe, M.; Gridnev, I.D.; Takao Ikariya, T.; J. Am. Chem. Soc., 130, 2158–2159, 2008.
23735 65 8 208361 Hasegawa, Y.; Gridnev, I.D.; Ikariya, T.; Angew. Chem., Int. Ed., 49, 8157–8160, 2010.
45167 1 1 61178 Blackwell, J.A.; Stringham, R.W.; Xiang, D.; Waltermire, R.E.; J. Chromatogr., A, 852, 383–394, 1999.
45167 1 2 142955 Navarre, L.; Martinez, R.; Genet, J–P.; Darses, S.; J. Am. Chem. Soc., 130, 6159–6169, 2008.
45167 1 3 61174 Blackwell, J.A.; Stringham, R.W.; Xiang, D.; Waltermire, R.E.; J. Chromatogr., A, 852, 383–394, 1999.
45167 1 4 200749 Huang, L.; Zhang, Y.; Staples, R.J.; Huang, R.H.; Wulff, W.D.; Chem. Eur. J., 18, 5302–5313, 2012.
45167 2 1 61176 Blackwell, J.A.; Stringham, R.W.; Xiang, D.; Waltermire, R.E.; J. Chromatogr., A, 852, 383–394, 1999.
45167 2 2 58927 Kim, B–H.; Lee, W.; Bull. Korean Chem. Soc., 19, 289–290, 1998.
45167 3 1 61180 Blackwell, J.A.; Stringham, R.W.; Xiang, D.; Waltermire, R.E.; J. Chromatogr., A, 852, 383–394, 1999.
45167 3 2 142955 Navarre, L.; Martinez, R.; Genet, J–P.; Darses, S.; J. Am. Chem. Soc., 130, 6159–6169, 2008.
45167 4 1 61176 Blackwell, J.A.; Stringham, R.W.; Xiang, D.; Waltermire, R.E.; J. Chromatogr., A, 852, 383–394, 1999.

45167 4 2 200749 Huang, L.; Zhang, Y.; Staples, R.J.; Huang, R.H.; Wulff, W.D.; Chem. Eur. J., 18, 5302–5313, 2012.
45167 5 1 61178 Blackwell, J.A.; Stringham, R.W.; Xiang, D.; Waltermire, R.E.; J. Chromatogr., A, 852, 383–394, 1999.
45167 5 2 58927 Kim, B–H.; Lee, W.; Bull. Korean Chem. Soc., 19, 289–290, 1998.
45167 6 1 206707 Hoffman, T.J.; Carreira, E.M.; Angew. Chem., Int. Ed., 50, 10670–10674, 2011.
45167 6 2 164386 Gendrineau, T.; Genet, J.–P.; Darses, S.; Org. Lett. , 11, 3486–3489, 2009.
45167 7 1 61174 Blackwell, J.A.; Stringham, R.W.; Xiang, D.; Waltermire, R.E.; J. Chromatogr., A, 852, 383–394, 1999.
45167 7 2 80281 Kawabata, T.; Suzuki, H.; Nagae, Y.; Fuji, K.; Angew. Chem., Int. Ed., 39, 2155–2157, 2000.
45167 8 1 206707 Hoffman, T.J.; Carreira, E.M.; Angew. Chem., Int. Ed., 50, 10670–10674, 2011.
45167 8 2 164387 Gendrineau, T.; Genet, J.–P.; Darses, S.; Org. Lett. , 11, 3486–3489, 2009.
45167 9 1 206707 Hoffman, T.J.; Carreira, E.M.; Angew. Chem., Int. Ed., 50, 10670–10674, 2011.
45167 9 2 158258 Feng, C–G.; Wang, Z–Q.; Tian, P.; Xu, M–H.; Lin, G–Q.; Chem. Asian J., 3, 1511–1516, 2008.
45167 10 1 61174 Blackwell, J.A.; Stringham, R.W.; Xiang, D.; Waltermire, R.E.; J. Chromatogr., A, 852, 383–394, 1999.
45167 10 2 58927 Kim, B–H.; Lee, W.; Bull. Korean Chem. Soc., 19, 289–290, 1998.
45167 11 1 115521 Guo, H.M.; Cun, L.F.; Gong, L.Z.; Mi, A.Q.; Jiang, Y.Z.; Chem. Commun. (Cambridge), 1450–1452, 2005.
45167 11 2 145713 Chen, J–R.; An, X–L.; Zhu, X–Y.; Wang, X–F.; Xiao, W–J.; J. Org. Chem., 73, 6006–6009, 2008.
45167 12 1 58931 Kim, B–H.; Lee, W.; Bull. Korean Chem. Soc., 19, 289–290, 1998.
45167 12 2 90245 Sibi, M.P.; Asano, Y.; Sausker, J.B.; Angew. Chem., Int. Ed., 40, 1293–1296, 2001.
45167 13 1 115521 Guo, H.M.; Cun, L.F.; Gong, L.Z.; Mi, A.Q.; Jiang, Y.Z.; Chem. Commun. (Cambridge), 1450–1452, 2005.
45167 13 2 199352 Chen, X.H.; Luo, S.W.; Tang, Z.; Cun, L.F.; Mi, A.Q.; Jiang, Y.Z.; Gong, L.Z.; Chem. Eur. J., 13, 689–701, 2007.
45167 14 1 61170 Blackwell, J.A.; Stringham, R.W.; Xiang, D.; Waltermire, R.E.; J. Chromatogr., A, 852, 383–394, 1999.
45167 14 2 80281 Kawabata, T.; Suzuki, H.; Nagae, Y.; Fuji, K.; Angew. Chem., Int. Ed., 39, 2155–2157, 2000.
45167 15 1 58931 Kim, B–H.; Lee, W.; Bull. Korean Chem. Soc., 19, 289–290, 1998.
45167 15 2 116056 CHIRAL TECHNOLOGIES EUROPE; Application Guide Software, 2006.
45167 16 1 180122 Bernasconi, G.; Bromidge, S.M.; Carpenter, A.J.; D'Adamo, L.; Di Fabio, R.; Guery, S.; Pavone, F.; Pozzan, A.; Rinaldi, M.; Sabbatini, F.M.; St–Denis, Y.; ; US Patent Application 20100286152, 2010.

45167 16 2 180115 Bernasconi, G.; Bromidge, S.M.; Carpenter, A.J.; D'Adamo, L.; Di Fabio, R.; Guery, S.; Pavone, F.; Pozzan, A.; Rinaldi, M.; Sabbatini, F.M.; St–Denis, Y.; ; US Patent Application 20100286152, 2010.

45167 17 1 58930 Kim, B–H.; Lee, W.; Bull. Korean Chem. Soc., 19, 289–290, 1998.

45167 17 2 116056 CHIRAL TECHNOLOGIES EUROPE; Application Guide Software, 2006.

45167 18 1 118705 Lu, S.M.; Han, X.W.; Zhou, Y.G.; Adv. Synth. Catal., 346, 909–912, 2004.

45167 18 2 209806 Zhao, D.B.; Beiring, B.; Glorius, F.; Angew. Chem., Int. Ed., 52, 8454–8458, 2013.

45167 19 1 61170 Blackwell, J.A.; Stringham, R.W.; Xiang, D.; Waltermire, R.E.; J. Chromatogr., A, 852, 383–394, 1999.

45167 19 2 58927 Kim, B–H.; Lee, W.; Bull. Korean Chem. Soc., 19, 289–290, 1998.

45167 20 1 115521 Guo, H.M.; Cun, L.F.; Gong, L.Z.; Mi, A.Q.; Jiang, Y.Z.; Chem. Commun. (Cambridge), 1450–1452, 2005.

45167 20 2 141244 Zhou, Y.; Shan, Z.; Tetrahedron: Asymmetry, 17, 1671–1677, 2006.

45167 21 1 206707 Hoffman, T.J.; Carreira, E.M.; Angew. Chem., Int. Ed., 50, 10670–10674, 2011.

45167 21 2 206709 Hoffman, T.J.; Carreira, E.M.; Angew. Chem., Int. Ed., 50, 10670–10674, 2011.

45167 22 1 118705 Lu, S.M.; Han, X.W.; Zhou, Y.G.; Adv. Synth. Catal., 346, 909–912, 2004.

45167 22 2 152426 Chinigo, G.M.; Paige, M.; Grindrod, S.; Hamel, E.; Dakshanamurthy, S.; Chruszcz, M.; Minor, W.; Brown, M.L.; J. Med. Chem., 51, 4620–4631, 2008.

45167 23 1 181229 Yamazaki, S.; Kashima, S.; Kuriyama, T.; Iwata, Y.; Morimoto, T.; Kakiuchi, K.; Tetrahedron: Asymmetry, 20, 1224–1234, 2009.

45167 23 2 181223 Yamazaki, S.; Kashima, S.; Kuriyama, T.; Iwata, Y.; Morimoto, T.; Kakiuchi, K.; Tetrahedron: Asymmetry, 20, 1224–1234, 2009.

45167 24 1 180122 Bernasconi, G.; Bromidge, S.M.; Carpenter, A.J.; D'Adamo, L.; Di Fabio, R.; Guery, S.; Pavone, F.; Pozzan, A.; Rinaldi, M.; Sabbatini, F.M.; St–Denis, Y.; ; US Patent Application 20100286152, 2010.

45167 24 2 180120 Bernasconi, G.; Bromidge, S.M.; Carpenter, A.J.; D'Adamo, L.; Di Fabio, R.; Guery, S.; Pavone, F.; Pozzan, A.; Rinaldi, M.; Sabbatini, F.M.; St–Denis, Y.; ; US Patent Application 20100286152, 2010.

45167 25 1 61172 Blackwell, J.A.; Stringham, R.W.; Xiang, D.; Waltermire, R.E.; J. Chromatogr., A, 852, 383–394, 1999.

45167 25 2 80281 Kawabata, T.; Suzuki, H.; Nagae, Y.; Fuji, K.; Angew. Chem., Int. Ed., 39, 2155–2157, 2000.

45167 26 1 58933 Kim, B–H.; Lee, W.; Bull. Korean Chem. Soc., 19, 289–290, 1998.

45167 26 2 152820 Hatano, M.; Maki, T.; Moriyama, K;. Manabu Arinobe, M.; Ishihara, K.; J. Am. Chem. Soc., 130, 16858–16860, 2008.

45167 27 1 61172 Blackwell, J.A.; Stringham, R.W.; Xiang, D.; Waltermire, R.E.; J. Chromatogr., A, 852, 383–394, 1999.

45167 27 2 58927 Kim, B–H.; Lee, W.; Bull. Korean Chem. Soc., 19, 289–290, 1998.

45167 28 1 168178 Lipka, E.; Vaccher, C.; Bonte, J.P.; Chirality, 21, 769–776, 2009.

45167 28 2 168190 Lipka, E.; Vaccher, C.; Bonte, J.P.; Chirality, 21, 769–776, 2009.

45167 28 3 168195 Lipka, E.; Vaccher, C.; Bonte, J.P.; Chirality, 21, 769–776, 2009.

45167 28 4 168206 Lipka, E.; Vaccher, C.; Bonte, J.P.; Chirality, 21, 769–776, 2009.

45167 29 1 164148 Hatano, M.; Hattori, Y.; Furuya, Y.; Ishihara, K.; Org. Lett. , 11, 2321–2324, 2009.

45167 29 2 164151 Hatano, M.; Hattori, Y.; Furuya, Y.; Ishihara, K.; Org. Lett. , 11, 2321–2324, 2009.

45167 30 1 164148 Hatano, M.; Hattori, Y.; Furuya, Y.; Ishihara, K.; Org. Lett. , 11, 2321–2324, 2009.

45167 30 2 113038 Jiao, Z.; Feng, X.; Liu, B.; Chen, F.; Zhang, G.; Jiang, Y.; Eur. J. Org. Chem., 3818–3826, 2003.

45172 1 1 115979 CHIRAL TECHNOLOGIES EUROPE; Application Guide Software, 2006.

45172 1 2 136787 Ghanem, A.; Al–Humaidi, E.; Chirality, 19, 477–484, 2007.

45172 2 1 209498 Chu, W.D.; Zhang, L.F.; Bao, X.; Zhao, X.H.; Zeng, C.; Du, J.Y.; Zhang, G.B.; Wang, F.X.; Ma, X.Y.; Fan, C.A.; Angew. Chem., Int. Ed., 52, 9229–9233, 2013.

45172 2 2 209494 Chu, W.D.; Zhang, L.F.; Bao, X.; Zhao, X.H.; Zeng, C.; Du, J.Y.; Zhang, G.B.; Wang, F.X.; Ma, X.Y.; Fan, C.A.; Angew. Chem., Int. Ed., 52, 9229–9233, 2013.

45172 3 1 183384 Galemmo, R.A.; Artis, D.R.; Ye, X.M.; Aubele, D.; Truong, A.; Bowers, S.; Hom, R.K.; Zhu, Y.–L.; Neitz, R.J.; Sealy, J.; Adler, M.; Beroza, P.; Anderson, J.P.; US Patent Application 20110207716, 201|1.

45172 3 2 183380 Galemmo, R.A.; Artis, D.R.; Ye, X.M.; Aubele, D.; Truong, A.; Bowers, S.; Hom, R.K.; Zhu, Y.–L.; Neitz, R.J.; Sealy, J.; Adler, M.; Beroza, P.; Anderson, J.P.; US Patent Application 20110207716, 201|1.

45172 4 1 183384 Galemmo, R.A.; Artis, D.R.; Ye, X.M.; Aubele, D.; Truong, A.; Bowers, S.; Hom, R.K.; Zhu, Y.–L.; Neitz, R.J.; Sealy, J.; Adler, M.; Beroza, P.; Anderson, J.P.; US Patent Application 20110207716, 201|1.

45172 4 2 183381 Galemmo, R.A.; Artis, D.R.; Ye, X.M.; Aubele, D.; Truong, A.; Bowers, S.; Hom, R.K.; Zhu, Y.–L.; Neitz, R.J.; Sealy, J.; Adler, M.; Beroza, P.; Anderson, J.P.; US Patent Application 20110207716, 201|1.

45172 5 1 115858 CHIRAL TECHNOLOGIES EUROPE; Application Guide Software, 2006.

45172 5 2 188645 Mangas–Sanchez, J.; Busto, E.; Gotor–Fernandez, V.; Malpartida, F.; Gotor, V.; J. Org. Chem, 76, 2115–2122, 2011.

45172 6 1 115979 CHIRAL TECHNOLOGIES EUROPE; Application Guide Software, 2006.

45172 6 2 136773 Ghanem, A.; Al–Humaidi, E.; Chirality, 19, 477–484, 2007.

45172 7 1 168530 Jin, J.Y.; Bae, S.K.; Lee, W.; Chirality, 21, 871–877, 2009.

45172 7 2 168470 Jin, J.Y.; Bae, S.K.; Lee, W.; Chirality, 21, 871–877, 2009.

45172 8 1 209498 Chu, W.D.; Zhang, L.F.; Bao, X.; Zhao, X.H.; Zeng, C.; Du, J.Y.; Zhang, G.B.; Wang, F.X.; Ma, X.Y.; Fan, C.A.; Angew. Chem., Int. Ed., 52, 9229–9233, 2013.

45172 8 2 209491 Chu, W.D.; Zhang, L.F.; Bao, X.; Zhao, X.H.; Zeng, C.; Du, J.Y.; Zhang, G.B.; Wang, F.X.; Ma, X.Y.; Fan, C.A.; Angew. Chem., Int. Ed., 52, 9229–9233, 2013.

45172 9 1 209498 Chu, W.D.; Zhang, L.F.; Bao, X.; Zhao, X.H.; Zeng, C.; Du, J.Y.; Zhang, G.B.; Wang, F.X.; Ma, X.Y.; Fan, C.A.; Angew. Chem., Int. Ed., 52, 9229–9233, 2013.

45172 9 2 209495 Chu, W.D.; Zhang, L.F.; Bao, X.; Zhao, X.H.; Zeng, C.; Du, J.Y.; Zhang, G.B.; Wang, F.X.; Ma, X.Y.; Fan, C.A.; Angew. Chem., Int. Ed., 52, 9229–9233, 2013.

45172 10 1 209498 Chu, W.D.; Zhang, L.F.; Bao, X.; Zhao, X.H.; Zeng, C.; Du, J.Y.; Zhang, G.B.; Wang, F.X.; Ma, X.Y.; Fan, C.A.; Angew. Chem., Int. Ed., 52, 9229–9233, 2013.
45172 10 2 209489 Chu, W.D.; Zhang, L.F.; Bao, X.; Zhao, X.H.; Zeng, C.; Du, J.Y.; Zhang, G.B.; Wang, F.X.; Ma, X.Y.; Fan, C.A.; Angew. Chem., Int. Ed., 52, 9229–9233, 2013.
45172 11 1 204378 Cao, T.; Deitch, J.; Linton, E.C.; Kozlowski, M.C.; Angew. Chem., Int. Ed., 51, 2448–2451, 2012.
45172 11 2 204377 Cao, T.; Deitch, J.; Linton, E.C.; Kozlowski, M.C.; Angew. Chem., Int. Ed., 51, 2448–2451, 2012.
45173 1 1 88193 Sarac, S.; Tozkoparan, B.; Ertan, M.; ; FABAD J. Pharm. Sci., 25, 145–151, 2000.
45173 1 2 88167 Sarac, S.; Tozkoparan, B.; Ertan, M.; ; FABAD J. Pharm. Sci., 25, 145–151, 2000.
45173 1 3 88194 Sarac, S.; Tozkoparan, B.; Ertan, M.; ; FABAD J. Pharm. Sci., 25, 145–151, 2000.
45173 1 4 88168 Sarac, S.; Tozkoparan, B.; Ertan, M.; ; FABAD J. Pharm. Sci., 25, 145–151, 2000.
45173 2 1 139706 Yarim, M.; Sarac, S.; Chromatographia, 56, 307–312, 2002.
45173 2 2 139810 Yarim, M.; Sarac, S.; Chromatographia, 56, 307–312, 2002.
45173 2 3 139754 Yarim, M.; Sarac, S.; Chromatographia, 56, 307–312, 2002.
45173 2 4 139822 Yarim, M.; Sarac, S.; Chromatographia, 56, 307–312, 2002.
45173 2 5 139778 Yarim, M.; Sarac, S.; Chromatographia, 56, 307–312, 2002.
45173 2 6 139828 Yarim, M.; Sarac, S.; Chromatographia, 56, 307–312, 2002.
45173 2 7 139730 Yarim, M.; Sarac, S.; Chromatographia, 56, 307–312, 2002.
45173 2 8 139816 Yarim, M.; Sarac, S.; Chromatographia, 56, 307–312, 2002.
45173 2 9 139714 Yarim, M.; Sarac, S.; Chromatographia, 56, 307–312, 2002.
45173 2 10 139812 Yarim, M.; Sarac, S.; Chromatographia, 56, 307–312, 2002.
45173 2 11 139762 Yarim, M.; Sarac, S.; Chromatographia, 56, 307–312, 2002.
45173 2 12 139824 Yarim, M.; Sarac, S.; Chromatographia, 56, 307–312, 2002.
45173 2 13 139746 Yarim, M.; Sarac, S.; Chromatographia, 56, 307–312, 2002.
45173 2 14 139820 Yarim, M.; Sarac, S.; Chromatographia, 56, 307–312, 2002.
45173 2 15 139770 Yarim, M.; Sarac, S.; Chromatographia, 56, 307–312, 2002.
45173 2 16 139826 Yarim, M.; Sarac, S.; Chromatographia, 56, 307–312, 2002.
45173 2 17 139738 Yarim, M.; Sarac, S.; Chromatographia, 56, 307–312, 2002.
45173 2 18 139818 Yarim, M.; Sarac, S.; Chromatographia, 56, 307–312, 2002.
45173 2 19 139722 Yarim, M.; Sarac, S.; Chromatographia, 56, 307–312, 2002.
45173 2 20 139814 Yarim, M.; Sarac, S.; Chromatographia, 56, 307–312, 2002.
45173 2 21 139796 Yarim, M.; Sarac, S.; Chromatographia, 56, 307–312, 2002.
45173 2 22 139832 Yarim, M.; Sarac, S.; Chromatographia, 56, 307–312, 2002.
45173 2 23 139788 Yarim, M.; Sarac, S.; Chromatographia, 56, 307–312, 2002.
45173 2 24 139830 Yarim, M.; Sarac, S.; Chromatographia, 56, 307–312, 2002.
45173 3 1 88193 Sarac, S.; Tozkoparan, B.; Ertan, M.; ; FABAD J. Pharm. Sci., 25, 145–151, 2000.

45173 3 2 88179 Sarac, S.; Tozkoparan, B.; Ertan, M.; ; FABAD J. Pharm. Sci., 25, 145–151, 2000.
45173 3 3 88194 Sarac, S.; Tozkoparan, B.; Ertan, M.; ; FABAD J. Pharm. Sci., 25, 145–151, 2000.
45173 3 4 88180 Sarac, S.; Tozkoparan, B.; Ertan, M.; ; FABAD J. Pharm. Sci., 25, 145–151, 2000.
45173 4 1 111538 Liu, L.; Pu, L.; Tetrahedron, 60, 7427–7430, 2004.
45173 4 2 126248 Mao, J.; Wan, B.; Wu, F.; Lu, S.; J. Mol. Catal. A: Chem., 237, 126–131, 2005.
45173 4 3 111549 Liu, L.; Pu, L.; Tetrahedron, 60, 7427–7430, 2004.
45173 4 4 136221 Lu, G.; Li, X.; Li, Y.M.; Kwong, F.Y.; Chan, A.S.C.; Adv. Synth. Catal., 348, 1926–1933, 2006.
45173 4 5 143517 Xu, Z.; Mao, J.; Zhang, Y.; Org. Biomol. Chem., 6, 1288–1292, 2008.
45173 4 6 126252 Mao, J.; Wan, B.; Wu, F.; Lu, S.; J. Mol. Catal. A: Chem., 237, 126–131, 2005.
45173 4 7 111548 Liu, L.; Pu, L.; Tetrahedron, 60, 7427–7430, 2004.
45173 4 8 113169 Cozzi, G.; Angew. Chem., Int. Ed., 42, 2895–2898, 2003.
45173 4 9 118245 Watts, C.C.; Thoniyot, P.; Hirayama, L.C.; Romano, T.; Singaram, B.; Tetrahedron: Asymmetry, 16, 1829–1835, 2005.
45173 4 10 113170 Cozzi, G.; Angew. Chem., Int. Ed., 42, 2895–2898, 2003.
45173 4 11 111551 Liu, L.; Pu, L.; Tetrahedron, 60, 7427–7430, 2004.
45173 4 12 136218 Lu, G.; Li, X.; Li, Y.M.; Kwong, F.Y.; Chan, A.S.C.; Adv. Synth. Catal., 348, 1926–1933, 2006.
45173 4 13 111552 Liu, L.; Pu, L.; Tetrahedron, 60, 7427–7430, 2004.
45173 4 14 136224 Lu, G.; Li, X.; Li, Y.M.; Kwong, F.Y.; Chan, A.S.C.; Adv. Synth. Catal., 348, 1926–1933, 2006.
45173 4 15 58834 Gao, X.; Kagan, H.B.; Chirality, 10, 120–124, 1998.
45173 4 16 108066 de Vries, A.H.M.; Dissertation Rijksuniversiteit Groningen, 1996.
45173 4 17 111546 Liu, L.; Pu, L.; Tetrahedron, 60, 7427–7430, 2004.
45173 4 18 117952 Zhou, Y.; Wang, R.; Xu, Z.; Yan, W.; Liu, L.; Kang, Y.; Han, Z.; Org. Lett., 6, 4147–4149, 2004.
45173 4 19 111547 Liu, L.; Pu, L.; Tetrahedron, 60, 7427–7430, 2004.
45173 4 20 139147 Pathak, K.; Bhatt, A.P.; Abdi, S.H.P.; Kureshy, R.I.; Khan, N.U.H.; Ahmad, I.; Jasra, R.V.; Chirality, 19, 82–88, 2007.
45173 5 1 88157 Sarac, S.; Tozkoparan, B.; Ertan, M.; ; FABAD J. Pharm. Sci., 25, 145–151, 2000.
45173 5 2 88179 Sarac, S.; Tozkoparan, B.; Ertan, M.; ; FABAD J. Pharm. Sci., 25, 145–151, 2000.
45173 5 3 88158 Sarac, S.; Tozkoparan, B.; Ertan, M.; ; FABAD J. Pharm. Sci., 25, 145–151, 2000.
45173 5 4 88180 Sarac, S.; Tozkoparan, B.; Ertan, M.; ; FABAD J. Pharm. Sci., 25, 145–151, 2000.

45173 5 5 88159 Sarac, S.; Tozkoparan, B.; Ertan, M.; ; FABAD J. Pharm. Sci., 25, 145–151, 2000.
45173 5 6 88181 Sarac, S.; Tozkoparan, B.; Ertan, M.; ; FABAD J. Pharm. Sci., 25, 145–151, 2000.
45173 5 7 88160 Sarac, S.; Tozkoparan, B.; Ertan, M.; ; FABAD J. Pharm. Sci., 25, 145–151, 2000.
45173 5 8 88182 Sarac, S.; Tozkoparan, B.; Ertan, M.; ; FABAD J. Pharm. Sci., 25, 145–151, 2000.
45173 6 1 88157 Sarac, S.; Tozkoparan, B.; Ertan, M.; ; FABAD J. Pharm. Sci., 25, 145–151, 2000.
45173 6 2 88197 Sarac, S.; Tozkoparan, B.; Ertan, M.; ; FABAD J. Pharm. Sci., 25, 145–151, 2000.
45173 6 3 88160 Sarac, S.; Tozkoparan, B.; Ertan, M.; ; FABAD J. Pharm. Sci., 25, 145–151, 2000.
45173 6 4 88200 Sarac, S.; Tozkoparan, B.; Ertan, M.; ; FABAD J. Pharm. Sci., 25, 145–151, 2000.
45173 6 5 88159 Sarac, S.; Tozkoparan, B.; Ertan, M.; ; FABAD J. Pharm. Sci., 25, 145–151, 2000.
45173 6 6 88199 Sarac, S.; Tozkoparan, B.; Ertan, M.; ; FABAD J. Pharm. Sci., 25, 145–151, 2000.
45173 6 7 88158 Sarac, S.; Tozkoparan, B.; Ertan, M.; ; FABAD J. Pharm. Sci., 25, 145–151, 2000.
45173 6 8 88198 Sarac, S.; Tozkoparan, B.; Ertan, M.; ; FABAD J. Pharm. Sci., 25, 145–151, 2000.
45173 7 1 88157 Sarac, S.; Tozkoparan, B.; Ertan, M.; ; FABAD J. Pharm. Sci., 25, 145–151, 2000.
45173 7 2 88167 Sarac, S.; Tozkoparan, B.; Ertan, M.; ; FABAD J. Pharm. Sci., 25, 145–151, 2000.
45173 7 3 88158 Sarac, S.; Tozkoparan, B.; Ertan, M.; ; FABAD J. Pharm. Sci., 25, 145–151, 2000.
45173 7 4 88168 Sarac, S.; Tozkoparan, B.; Ertan, M.; ; FABAD J. Pharm. Sci., 25, 145–151, 2000.
45173 7 5 88160 Sarac, S.; Tozkoparan, B.; Ertan, M.; ; FABAD J. Pharm. Sci., 25, 145–151, 2000.
45173 7 6 88170 Sarac, S.; Tozkoparan, B.; Ertan, M.; ; FABAD J. Pharm. Sci., 25, 145–151, 2000.
45173 7 7 88159 Sarac, S.; Tozkoparan, B.; Ertan, M.; ; FABAD J. Pharm. Sci., 25, 145–151, 2000.
45173 7 8 88169 Sarac, S.; Tozkoparan, B.; Ertan, M.; ; FABAD J. Pharm. Sci., 25, 145–151, 2000.
45173 8 1 88194 Sarac, S.; Tozkoparan, B.; Ertan, M.; ; FABAD J. Pharm. Sci., 25, 145–151, 2000.

45173 8 2 88162 Sarac, S.; Tozkoparan, B.; Ertan, M.; ; FABAD J. Pharm. Sci., 25, 145–151, 2000.
45173 9 1 83141 Tozkoparan, B.; Yarim, M.; Sarac, S.; Ertan, M.; Kelicen, P.; Altinok, G.; Demirdamar, R.; Arch. Pharm. Pharm. Med. Chem., 333, 415–420, 2000.
45173 9 2 83144 Tozkoparan, B.; Yarim, M.; Sarac, S.; Ertan, M.; Kelicen, P.; Altinok, G.; Demirdamar, R.; Arch. Pharm. Pharm. Med. Chem., 333, 415–420, 2000.
45173 9 3 88193 Sarac, S.; Tozkoparan, B.; Ertan, M.; ; FABAD J. Pharm. Sci., 25, 145–151, 2000.
45173 9 4 88197 Sarac, S.; Tozkoparan, B.; Ertan, M.; ; FABAD J. Pharm. Sci., 25, 145–151, 2000.
45173 9 5 88194 Sarac, S.; Tozkoparan, B.; Ertan, M.; ; FABAD J. Pharm. Sci., 25, 145–151, 2000.
45173 9 6 88198 Sarac, S.; Tozkoparan, B.; Ertan, M.; ; FABAD J. Pharm. Sci., 25, 145–151, 2000.
45173 10 1 22016 Ader, U.; Muschalek, V.; Schneider, M.P.; Chirality, 5, 554–559, 1993.
45173 10 2 22017 Ader, U.; Muschalek, V.; Schneider, M.P.; Chirality, 5, 554–559, 1993.
45173 11 1 70314 Aboul–enein, H.Y.; Abou–basha, L.I.; Bakr, S.A.; Chirality, 8, 153–156, 1996.
45173 11 2 70302 Aboul–enein, H.Y.; Abou–basha, L.I.; Bakr, S.A.; Chirality, 8, 153–156, 1996.
45173 11 3 70311 Aboul–enein, H.Y.; Abou–basha, L.I.; Bakr, S.A.; Chirality, 8, 153–156, 1996.
45173 11 4 70299 Aboul–enein, H.Y.; Abou–basha, L.I.; Bakr, S.A.; Chirality, 8, 153–156, 1996.
45173 12 1 22016 Ader, U.; Muschalek, V.; Schneider, M.P.; Chirality, 5, 554–559, 1993.
45173 12 2 72081 Svensson, S.; Karlsson, A.; Gyllenhaal, O.; Vessman, J.; Chromatographia, 51, 283–293, 2000.
45173 13 1 12258 Wu, S–H.; Lai, S–Y.; Lin, S–L.; Chu, F–Y.; Wang, K–T.; Chirality, 3, 67–70, 1991.
45173 13 2 12253 Wu, S–H.; Lai, S–Y.; Lin, S–L.; Chu, F–Y.; Wang, K–T.; Chirality, 3, 67–70, 1991.
45173 13 3 59618 Azzolina, O.; Collina, S.; Ghislandi, V.; Farmaco, 48, 1401–1416, 1993.
45173 13 4 59602 Azzolina, O.; Collina, S.; Ghislandi, V.; Farmaco, 48, 1401–1416, 1993.
45173 13 5 12564 Ghislandi, V.; Azzolina, O.; Vercesi, D.; Mariotti, A.; International Symposium on Chiral Discrimination (2nd), Roma, 1991.
45173 13 6 16609 Lin, S.–L.; Chiou, A.–J.; Wu, S.–H.; Wang, K.–T.; Chirality, 3, 476–479, 1991.
45173 14 1 22016 Ader, U.; Muschalek, V.; Schneider, M.P.; Chirality, 5, 554–559, 1993.
45173 14 2 72053 Svensson, S.; Karlsson, A.; Gyllenhaal, O.; Vessman, J.; Chromatographia, 51, 283–293, 2000.
45173 15 1 40461 Azzolina, O.; Vercesi, D.; Collina, S.; Ghislandi, V.; Farmaco, 50(4), 221–226, 1995.

45173 15 2 40464 Azzolina, O.; Vercesi, D.; Collina, S.; Ghislandi, V.; Farmaco, 50(4), 221–226, 1995.
45173 15 3 40462 Azzolina, O.; Vercesi, D.; Collina, S.; Ghislandi, V.; Farmaco, 50(4), 221–226, 1995.
45173 15 4 40466 Azzolina, O.; Vercesi, D.; Collina, S.; Ghislandi, V.; Farmaco, 50(4), 221–226, 1995.
45173 15 5 59590 Azzolina, O.; Collina, S.; Ghislandi, V.; Farmaco, 48, 1401–1416, 1993.
45173 15 6 59591 Azzolina, O.; Collina, S.; Ghislandi, V.; Farmaco, 48, 1401–1416, 1993.
45173 16 1 70314 Aboul–enein, H.Y.; Abou–basha, L.I.; Bakr, S.A.; Chirality, 8, 153–156, 1996.
45173 16 2 52324 Bargmann–Leyder, N.; Tambuté, A.; Caude, M.; Chirality, 7, 311–325, 1995.
45173 16 3 70311 Aboul–enein, H.Y.; Abou–basha, L.I.; Bakr, S.A.; Chirality, 8, 153–156, 1996.
45173 16 4 52326 Bargmann–Leyder, N.; Tambuté, A.; Caude, M.; Chirality, 7, 311–325, 1995.
45173 17 1 88157 Sarac, S.; Tozkoparan, B.; Ertan, M.; ; FABAD J. Pharm. Sci., 25, 145–151, 2000.
45173 17 2 88188 Sarac, S.; Tozkoparan, B.; Ertan, M.; ; FABAD J. Pharm. Sci., 25, 145–151, 2000.
45173 17 3 88160 Sarac, S.; Tozkoparan, B.; Ertan, M.; ; FABAD J. Pharm. Sci., 25, 145–151, 2000.
45173 17 4 88192 Sarac, S.; Tozkoparan, B.; Ertan, M.; ; FABAD J. Pharm. Sci., 25, 145–151, 2000.
45173 18 1 88158 Sarac, S.; Tozkoparan, B.; Ertan, M.; ; FABAD J. Pharm. Sci., 25, 145–151, 2000.
45173 18 2 88162 Sarac, S.; Tozkoparan, B.; Ertan, M.; ; FABAD J. Pharm. Sci., 25, 145–151, 2000.
45173 18 3 88159 Sarac, S.; Tozkoparan, B.; Ertan, M.; ; FABAD J. Pharm. Sci., 25, 145–151, 2000.
45173 18 4 88164 Sarac, S.; Tozkoparan, B.; Ertan, M.; ; FABAD J. Pharm. Sci., 25, 145–151, 2000.
45173 19 1 12554 Ghislandi, V.; Azzolina, O.; Vercesi, D.; Mariotti, A.; International Symposium on Chiral Discrimination (2nd), Roma, 1991.
45173 19 2 40464 Azzolina, O.; Vercesi, D.; Collina, S.; Ghislandi, V.; Farmaco, 50(4), 221–226, 1995.
45173 19 3 12555 Ghislandi, V.; Azzolina, O.; Vercesi, D.; Mariotti, A.; International Symposium on Chiral Discrimination (2nd), Roma, 1991.
45173 19 4 59591 Azzolina, O.; Collina, S.; Ghislandi, V.; Farmaco, 48, 1401–1416, 1993.
45173 19 5 12553 Ghislandi, V.; Azzolina, O.; Vercesi, D.; Mariotti, A.; International Symposium on Chiral Discrimination (2nd), Roma, 1991.
45173 19 6 40466 Azzolina, O.; Vercesi, D.; Collina, S.; Ghislandi, V.; Farmaco, 50(4), 221–226, 1995.

45173 20 1 107356 Cirilli, R.; Ferretti, R.; Gallinella, B.; Turchetto, L.; Bolasco, A.; Secci, D.; Chimenti, P.; Pierini, M.; ... la torre, F.; Chirality, 16, 625–636, 2004.
45173 20 2 107382 Cirilli, R.; Ferretti, R.; Gallinella, B.; Turchetto, L.; Bolasco, A.; Secci, D.; Chimenti, P.; Pierini, M.; ... la torre, F.; Chirality, 16, 625–636, 2004.
45173 20 3 107369 Cirilli, R.; Ferretti, R.; Gallinella, B.; Turchetto, L.; Bolasco, A.; Secci, D.; Chimenti, P.; Pierini, M.; ... la torre, F.; Chirality, 16, 625–636, 2004.
45173 20 4 107394 Cirilli, R.; Ferretti, R.; Gallinella, B.; Turchetto, L.; Bolasco, A.; Secci, D.; Chimenti, P.; Pierini, M.; ... la torre, F.; Chirality, 16, 625–636, 2004.
45173 21 1 22016 Ader, U.; Muschalek, V.; Schneider, M.P.; Chirality, 5, 554–559, 1993.
45173 21 2 27472 Vandenbosch, C.; Massart, D. L.; Lindner, W.; J. Pharm. Biomed. Anal., 10, 895–908, 1992.
45173 21 3 73 Okamoto, Y.; Aburatani, R.; Hatano, K.; Hatada, K.; J. Liq. Chromatogr., 11, 2147–2163, 1988.
45173 21 4 22256 Herring, V.L.; Johnson, J.A.; J. Chromatogr. Biomed. Appl., 612, 215–221, 1993.
45173 22 1 12258 Wu, S–H.; Lai, S–Y.; Lin, S–L.; Chu, F–Y.; Wang, K–T.; Chirality, 3, 67–70, 1991.
45173 22 2 111714 Focken, T.; Raabe, G.; Bolm, C.; Tetrahedron: Asymmetry, 15, 1693–1706, 2004.
45173 22 3 2077 Okamoto, Y.; Aburatani, R.; Kaida, Y.; Hatada, K.; Chem. Lett., 1125–1128, 1988.
45173 22 4 141368 Ueberbacher, B.J.; Grieng, H.; Weber, H.; Tetrahedron: Asymmetry, 19, 838–846, 2008.
45173 23 1 107356 Cirilli, R.; Ferretti, R.; Gallinella, B.; Turchetto, L.; Bolasco, A.; Secci, D.; Chimenti, P.; Pierini, M.; ... la torre, F.; Chirality, 16, 625–636, 2004.
45173 23 2 107430 Cirilli, R.; Ferretti, R.; Gallinella, B.; Turchetto, L.; Bolasco, A.; Secci, D.; Chimenti, P.; Pierini, M.; ... la torre, F.; Chirality, 16, 625–636, 2004.
45173 23 3 107369 Cirilli, R.; Ferretti, R.; Gallinella, B.; Turchetto, L.; Bolasco, A.; Secci, D.; Chimenti, P.; Pierini, M.; ... la torre, F.; Chirality, 16, 625–636, 2004.
45173 23 4 107442 Cirilli, R.; Ferretti, R.; Gallinella, B.; Turchetto, L.; Bolasco, A.; Secci, D.; Chimenti, P.; Pierini, M.; ... la torre, F.; Chirality, 16, 625–636, 2004.
45173 24 1 74 Okamoto, Y.; Aburatani, R.; Hatano, K.; Hatada, K.; J. Liq. Chromatogr., 11, 2147–2163, 1988.
45173 24 2 72081 Svensson, S.; Karlsson, A.; Gyllenhaal, O.; Vessman, J.; Chromatographia, 51, 283–293, 2000.
45173 25 1 88194 Sarac, S.; Tozkoparan, B.; Ertan, M.; ; FABAD J. Pharm. Sci., 25, 145–151, 2000.
45173 25 2 83141 Tozkoparan, B.; Yarim, M.; Sarac, S.; Ertan, M.; Kelicen, P.; Altinok, G.; Demirdamar, R.; Arch. Pharm. Pharm. Med. Chem., 333, 415–420, 2000.
45173 25 3 88198 Sarac, S.; Tozkoparan, B.; Ertan, M.; ; FABAD J. Pharm. Sci., 25, 145–151, 2000.
45173 25 4 83144 Tozkoparan, B.; Yarim, M.; Sarac, S.; Ertan, M.; Kelicen, P.; Altinok, G.; Demirdamar, R.; Arch. Pharm. Pharm. Med. Chem., 333, 415–420, 2000.

45173 26 1 22016 Ader, U.; Muschalek, V.; Schneider, M.P.; Chirality, 5, 554–559, 1993.

45173 26 2 70318 Aboul–enein, H.Y.; Abou–basha, L.I.; Bakr, S.A.; Chirality, 8, 153–156, 1996.

45173 27 1 88157 Sarac, S.; Tozkoparan, B.; Ertan, M.; ; FABAD J. Pharm. Sci., 25, 145–151, 2000.

45173 27 2 83144 Tozkoparan, B.; Yarim, M.; Sarac, S.; Ertan, M.; Kelicen, P.; Altinok, G.; Demirdamar, R.; Arch. Pharm. Pharm. Med. Chem., 333, 415–420, 2000.

45173 27 3 88160 Sarac, S.; Tozkoparan, B.; Ertan, M.; ; FABAD J. Pharm. Sci., 25, 145–151, 2000.

45173 27 4 83146 Tozkoparan, B.; Yarim, M.; Sarac, S.; Ertan, M.; Kelicen, P.; Altinok, G.; Demirdamar, R.; Arch. Pharm. Pharm. Med. Chem., 333, 415–420, 2000.

45173 28 1 88183 Sarac, S.; Tozkoparan, B.; Ertan, M.; ; FABAD J. Pharm. Sci., 25, 145–151, 2000.

45173 28 2 88167 Sarac, S.; Tozkoparan, B.; Ertan, M.; ; FABAD J. Pharm. Sci., 25, 145–151, 2000.

45173 28 3 88184 Sarac, S.; Tozkoparan, B.; Ertan, M.; ; FABAD J. Pharm. Sci., 25, 145–151, 2000.

45173 28 4 88168 Sarac, S.; Tozkoparan, B.; Ertan, M.; ; FABAD J. Pharm. Sci., 25, 145–151, 2000.

45173 29 1 139143 Pathak, K.; Bhatt, A.P.; Abdi, S.H.P.; Kureshy, R.I.; Khan, N.U.H.; Ahmad, I.; Jasra, R.V.; Chirality, 19, 82–88, 2007.

45173 29 2 151693 Li, H.; Da, C–S.; Xiao, Y–H.; Li, X.; Su, Y–N.; J. Org. Chem., 73, 7398–7401, 2008.

45173 29 3 10688 Niwa, S.; Soai, K.; J. Chem. Soc., Perkin Trans. 1, 937–943, 1990.

45173 29 4 24136 Parmee, E.R.; Hong, Y.; Tempkin, O.; Masamune, S.; Tetrahedron Lett., 33, 1729–1732, 1992.

45173 29 5 111553 Liu, L.; Pu, L.; Tetrahedron, 60, 7427–7430, 2004.

45173 29 6 125998 Itsuno, S.; Arima, S.; Haraguchi, N.; Tetrahedron, 61, 12074–12080, 2005.

45173 30 1 197212 Lundgren, S.; Wingstrand, E.; Moberg, C.; Adv. Synth. Catal., 349, 364–372, 2007.

45173 30 2 67293 Kenny, J.A.; Palmer, M.J.; Smith, A.R.C.; Walsgrove, T.; Wills, M.; Synlett, 1615–1617, 1999.

45173 31 1 2091 Okamoto, Y.; Aburatani, R.; Kaida, Y.; Hatada, K.; Chem. Lett., 1125–1128, 1988.

45173 31 2 24647 Miyazawa, T.; Shindo, Y.; Yamada, T.; Kuwata, S.; Anal. Lett., 26, 457–473, 1993.

45173 31 3 48090 Kim, B. H.; Lee, W.; Ok, J. H.; International Symposium on Chiral Discrimination (9th), Nagoya, 1997.

45173 31 4 24648 Miyazawa, T.; Shindo, Y.; Yamada, T.; Kuwata, S.; Anal. Lett., 26, 457–473, 1993.

45173 32 1 107369 Cirilli, R.; Ferretti, R.; Gallinella, B.; Turchetto, L.; Bolasco, A.; Secci, D.; Chimenti, P.; Pierini, M.; ... la torre, F.; Chirality, 16, 625–636, 2004.

45173 32 2 107406 Cirilli, R.; Ferretti, R.; Gallinella, B.; Turchetto, L.; Bolasco, A.; Secci, D.; Chimenti, P.; Pierini, M.; ... la torre, F.; Chirality, 16, 625–636, 2004.
45173 33 1 88183 Sarac, S.; Tozkoparan, B.; Ertan, M.; ; FABAD J. Pharm. Sci., 25, 145–151, 2000.
45173 33 2 88179 Sarac, S.; Tozkoparan, B.; Ertan, M.; ; FABAD J. Pharm. Sci., 25, 145–151, 2000.
45173 33 3 88184 Sarac, S.; Tozkoparan, B.; Ertan, M.; ; FABAD J. Pharm. Sci., 25, 145–151, 2000.
45173 33 4 88180 Sarac, S.; Tozkoparan, B.; Ertan, M.; ; FABAD J. Pharm. Sci., 25, 145–151, 2000.
45173 34 1 74 Okamoto, Y.; Aburatani, R.; Hatano, K.; Hatada, K.; J. Liq. Chromatogr., 11, 2147–2163, 1988.
45173 34 2 11565 Aboul–Enein, H.Y.; Islam, M.R.; Anal. Lett., 23(6), 973–980, 1990.
45173 35 1 88157 Sarac, S.; Tozkoparan, B.; Ertan, M.; ; FABAD J. Pharm. Sci., 25, 145–151, 2000.
45173 35 2 83149 Tozkoparan, B.; Yarim, M.; Sarac, S.; Ertan, M.; Kelicen, P.; Altinok, G.; Demirdamar, R.; Arch. Pharm. Pharm. Med. Chem., 333, 415–420, 2000.
45173 35 3 88160 Sarac, S.; Tozkoparan, B.; Ertan, M.; ; FABAD J. Pharm. Sci., 25, 145–151, 2000.
45173 35 4 83150 Tozkoparan, B.; Yarim, M.; Sarac, S.; Ertan, M.; Kelicen, P.; Altinok, G.; Demirdamar, R.; Arch. Pharm. Pharm. Med. Chem., 333, 415–420, 2000.
45173 36 1 12258 Wu, S–H.; Lai, S–Y.; Lin, S–L.; Chu, F–Y.; Wang, K–T.; Chirality, 3, 67–70, 1991.
45173 36 2 40459 Azzolina, O.; Vercesi, D.; Collina, S.; Ghislandi, V.; Farmaco, 50(4), 221–226, 1995.
45173 36 3 59618 Azzolina, O.; Collina, S.; Ghislandi, V.; Farmaco, 48, 1401–1416, 1993.
45173 36 4 59588 Azzolina, O.; Collina, S.; Ghislandi, V.; Farmaco, 48, 1401–1416, 1993.
45173 37 1 107356 Cirilli, R.; Ferretti, R.; Gallinella, B.; Turchetto, L.; Bolasco, A.; Secci, D.; Chimenti, P.; Pierini, M.; ... la torre, F.; Chirality, 16, 625–636, 2004.
45173 37 2 107406 Cirilli, R.; Ferretti, R.; Gallinella, B.; Turchetto, L.; Bolasco, A.; Secci, D.; Chimenti, P.; Pierini, M.; ... la torre, F.; Chirality, 16, 625–636, 2004.
45173 37 3 107369 Cirilli, R.; Ferretti, R.; Gallinella, B.; Turchetto, L.; Bolasco, A.; Secci, D.; Chimenti, P.; Pierini, M.; ... la torre, F.; Chirality, 16, 625–636, 2004.
45173 37 4 107418 Cirilli, R.; Ferretti, R.; Gallinella, B.; Turchetto, L.; Bolasco, A.; Secci, D.; Chimenti, P.; Pierini, M.; ... la torre, F.; Chirality, 16, 625–636, 2004.
45173 38 1 164494 Duguet, N.; Slawin, A.M.Z.; Smith, A.D.; Org. Lett. , 11, 3858–3861, 2009.
45173 38 2 89680 Lee, S.; Hartwig, J.F.; J. Org. Chem., 66, 3402–3415, 2001.
45173 38 3 164495 Duguet, N.; Slawin, A.M.Z.; Smith, A.D.; Org. Lett. , 11, 3858–3861, 2009.
45173 38 4 211972 Katayev, D.; Jia, Y.X.; Sharma, A.K.; Banerjee, D.; Besnard, C.; Sunoj, R.B.; Kundig, E.P.; Chemistry–A European Journal, 19, 11916–11927, 2013.

45173 39 1 88160 Sarac, S.; Tozkoparan, B.; Ertan, M.; ; FABAD J. Pharm. Sci., 25, 145–151, 2000.
45173 39 2 88188 Sarac, S.; Tozkoparan, B.; Ertan, M.; ; FABAD J. Pharm. Sci., 25, 145–151, 2000.
45173 40 1 115396 Cooper, T.S.; Larigo, A.S.; Laurent, P.; Moody, C.J.; Takle, A.K.; Org. Biomol. Chem., 3, 1252–1262, 2005.
45173 40 2 115395 Cooper, T.S.; Larigo, A.S.; Laurent, P.; Moody, C.J.; Takle, A.K.; Org. Biomol. Chem., 3, 1252–1262, 2005.
45173 41 1 105466 Hang, J.; Li, H.; Deng, L.; Org. Lett., 4, 3321–3324, 2002.
45173 41 2 24710 Miyazawa, T.; Shindo, Y.; Yamada, T.; Kuwata, S.; Anal. Lett., 26, 457–473, 1993.
45173 41 3 105468 Hang, J.; Li, H.; Deng, L.; Org. Lett., 4, 3321–3324, 2002.
45173 41 4 24709 Miyazawa, T.; Shindo, Y.; Yamada, T.; Kuwata, S.; Anal. Lett., 26, 457–473, 1993.
45173 41 5 2089 Okamoto, Y.; Aburatani, R.; Kaida, Y.; Hatada, K.; Chem. Lett., 1125–1128, 1988.
45173 41 6 24708 Miyazawa, T.; Shindo, Y.; Yamada, T.; Kuwata, S.; Anal. Lett., 26, 457–473, 1993.
45173 42 1 71 Okamoto, Y.; Aburatani, R.; Hatano, K.; Hatada, K.; J. Liq. Chromatogr., 11, 2147–2163, 1988.
45173 42 2 72081 Svensson, S.; Karlsson, A.; Gyllenhaal, O.; Vessman, J.; Chromatographia, 51, 283–293, 2000.
45173 43 1 2077 Okamoto, Y.; Aburatani, R.; Kaida, Y.; Hatada, K.; Chem. Lett., 1125–1128, 1988.
45173 43 2 12562 Ghislandi, V.; Azzolina, O.; Vercesi, D.; Mariotti, A.; International Symposium on Chiral Discrimination (2nd), Roma, 1991.
45173 43 3 29317 Grieb, S.J.; Matlin, S.A.; Belenguer, A.M.; International Symposium on Chiral Discrimination (6th), St Louis, 1995.
45173 43 4 136160 Cheng, X.; Xie, J.H.; Li, S.; Zhou, Q.L.; Adv. Synth. Catal., 348, 1271–1276, 2006.
45173 43 5 12558 Ghislandi, V.; Azzolina, O.; Vercesi, D.; Mariotti, A.; International Symposium on Chiral Discrimination (2nd), Roma, 1991.
45173 43 6 40472 Azzolina, O.; Vercesi, D.; Collina, S.; Ghislandi, V.; Farmaco, 50(4), 221–226, 1995.
45173 43 7 12553 Ghislandi, V.; Azzolina, O.; Vercesi, D.; Mariotti, A.; International Symposium on Chiral Discrimination (2nd), Roma, 1991.
45173 43 8 40466 Azzolina, O.; Vercesi, D.; Collina, S.; Ghislandi, V.; Farmaco, 50(4), 221–226, 1995.
45173 44 1 4631 Okamoto, Y.; Kaida, Y.; Aburatani, R.; Hatada, K.; J. Chromatogr., 477, 367–376, 1989.
45173 44 2 4647 Okamoto, Y.; Kaida, Y.; Aburatani, R.; Hatada, K.; J. Chromatogr., 477, 367–376, 1989.

45173 44 3 24647 Miyazawa, T.; Shindo, Y.; Yamada, T.; Kuwata, S.; Anal. Lett., 26, 457–473, 1993.

45173 44 4 106437 Miyaza, T.; Shindo, Y.; Yamada, T.; Chromatographia, 60, 419–424, 2004.

45173 44 5 4632 Okamoto, Y.; Kaida, Y.; Aburatani, R.; Hatada, K.; J. Chromatogr., 477, 367–376, 1989.

45173 44 6 4648 Okamoto, Y.; Kaida, Y.; Aburatani, R.; Hatada, K.; J. Chromatogr., 477, 367–376, 1989.

45173 45 1 197212 Lundgren, S.; Wingstrand, E.; Moberg, C.; Adv. Synth. Catal., 349, 364–372, 2007.

45173 45 2 3751 Okamoto, Y.; Cao, Z.K.; Aburatani, R.; Hatada, K.; Bull. Chem. Soc. Jpn., 60, 3999–4003, 1987.

45173 46 1 88160 Sarac, S.; Tozkoparan, B.; Ertan, M.; ; FABAD J. Pharm. Sci., 25, 145–151, 2000.

45173 46 2 88167 Sarac, S.; Tozkoparan, B.; Ertan, M.; ; FABAD J. Pharm. Sci., 25, 145–151, 2000.

45173 47 1 88183 Sarac, S.; Tozkoparan, B.; Ertan, M.; ; FABAD J. Pharm. Sci., 25, 145–151, 2000.

45173 47 2 88197 Sarac, S.; Tozkoparan, B.; Ertan, M.; ; FABAD J. Pharm. Sci., 25, 145–151, 2000.

45173 47 3 88184 Sarac, S.; Tozkoparan, B.; Ertan, M.; ; FABAD J. Pharm. Sci., 25, 145–151, 2000.

45173 47 4 88198 Sarac, S.; Tozkoparan, B.; Ertan, M.; ; FABAD J. Pharm. Sci., 25, 145–151, 2000.

45173 48 1 22016 Ader, U.; Muschalek, V.; Schneider, M.P.; Chirality, 5, 554–559, 1993.

45173 48 2 72075 Svensson, S.; Karlsson, A.; Gyllenhaal, O.; Vessman, J.; Chromatographia, 51, 283–293, 2000.

90201 1 1 22008 Wolf, R.M.; Francotte, E., Hainmüller, J.; Chirality, 5, 538–544, 1993.

90201 1 2 159 Wolf, R.M.; Francotte, E.; Lohmann, D.; J. Chem. Soc., Perkin Trans. 2, 893–901, 1988.

90201 1 3 165 Wolf, R.M.; Francotte, E.; Lohmann, D.; J. Chem. Soc., Perkin Trans. 2, 893–901, 1988.

90201 1 4 161 Wolf, R.M.; Francotte, E.; Lohmann, D.; J. Chem. Soc., Perkin Trans. 2, 893–901, 1988.

90201 1 5 163 Wolf, R.M.; Francotte, E.; Lohmann, D.; J. Chem. Soc., Perkin Trans. 2, 893–901, 1988.

90201 1 6 164 Wolf, R.M.; Francotte, E.; Lohmann, D.; J. Chem. Soc., Perkin Trans. 2, 893–901, 1988.

90201 2 1 115 Francotte, E.; Ackermann, P.; Chirality and Biological Activity, Eds. Frank H., Holmstedt B. and Testa B., LISS A.R. INC, New York, 63–70, 1990.

90201 2 2 105 Francotte, E.; Ackermann, P.; Chirality and Biological Activity, Eds. Frank H., Holmstedt B. and Testa B., LISS A.R. INC, New York, 63–70, 1990.

90201 2 3 116 Francotte, E.; Ackermann, P.; Chirality and Biological Activity, Eds. Frank H., Holmstedt B. and Testa B., LISS A.R. INC, New York, 63–70, 1990.

90201 2 4 106 Francotte, E.; Ackermann, P.; Chirality and Biological Activity, Eds. Frank H., Holmstedt B. and Testa B., LISS A.R. INC, New York, 63–70, 1990.

90201 2 5 117 Francotte, E.; Ackermann, P.; Chirality and Biological Activity, Eds. Frank H., Holmstedt B. and Testa B., LISS A.R. INC, New York, 63–70, 1990.

90201 2 6 107 Francotte, E.; Ackermann, P.; Chirality and Biological Activity, Eds. Frank H., Holmstedt B. and Testa B., LISS A.R. INC, New York, 63–70, 1990.

90201 2 7 118 Francotte, E.; Ackermann, P.; Chirality and Biological Activity, Eds. Frank H., Holmstedt B. and Testa B., LISS A.R. INC, New York, 63–70, 1990.

90201 2 8 108 Francotte, E.; Ackermann, P.; Chirality and Biological Activity, Eds. Frank H., Holmstedt B. and Testa B., LISS A.R. INC, New York, 63–70, 1990.

90201 2 9 120 Francotte, E.; Ackermann, P.; Chirality and Biological Activity, Eds. Frank H., Holmstedt B. and Testa B., LISS A.R. INC, New York, 63–70, 1990.

90201 2 10 110 Francotte, E.; Ackermann, P.; Chirality and Biological Activity, Eds. Frank H., Holmstedt B. and Testa B., LISS A.R. INC, New York, 63–70, 1990.

90201 2 11 121 Francotte, E.; Ackermann, P.; Chirality and Biological Activity, Eds. Frank H., Holmstedt B. and Testa B., LISS A.R. INC, New York, 63–70, 1990.

90201 2 12 111 Francotte, E.; Ackermann, P.; Chirality and Biological Activity, Eds. Frank H., Holmstedt B. and Testa B., LISS A.R. INC, New York, 63–70, 1990.

90201 2 13 122 Francotte, E.; Ackermann, P.; Chirality and Biological Activity, Eds. Frank H., Holmstedt B. and Testa B., LISS A.R. INC, New York, 63–70, 1990.

90201 2 14 112 Francotte, E.; Ackermann, P.; Chirality and Biological Activity, Eds. Frank H., Holmstedt B. and Testa B., LISS A.R. INC, New York, 63–70, 1990.

90201 2 15 114 Francotte, E.; Ackermann, P.; Chirality and Biological Activity, Eds. Frank H., Holmstedt B. and Testa B., LISS A.R. INC, New York, 63–70, 1990.

90201 2 16 113 Francotte, E.; Ackermann, P.; Chirality and Biological Activity, Eds. Frank H., Holmstedt B. and Testa B., LISS A.R. INC, New York, 63–70, 1990.

90220 1 1 31515 Lin, S.; J. Liq. Chromatogr., 18, 2611–2619, 1995.

90220 1 2 31521 Lin, S.; J. Liq. Chromatogr., 18, 2611–2619, 1995.

90220 2 1 31515 Lin, S.; J. Liq. Chromatogr., 18, 2611–2619, 1995.

90220 2 2 31518 Lin, S.; J. Liq. Chromatogr., 18, 2611–2619, 1995.

90220 3 1 129823 Calabro, M.L.; Raneri, D.; Tommasini, S.; Ficarra, R.; Alcaro, S.; Gallelli, A.; Micale, N.; Zappala, M.; Ficarra, P.; J. Chromatogr., B, 838, 56–62, 2006.

90220 3 2 129829 Calabro, M.L.; Raneri, D.; Tommasini, S.; Ficarra, R.; Alcaro, S.; Gallelli, A.; Micale, N.; Zappala, M.; Ficarra, P.; J. Chromatogr., B, 838, 56–62, 2006.

90220 4 1 129823 Calabro, M.L.; Raneri, D.; Tommasini, S.; Ficarra, R.; Alcaro, S.; Gallelli, A.; Micale, N.; Zappala, M.; Ficarra, P.; J. Chromatogr., B, 838, 56–62, 2006.

90220 4 2 129835 Calabro, M.L.; Raneri, D.; Tommasini, S.; Ficarra, R.; Alcaro, S.; Gallelli, A.; Micale, N.; Zappala, M.; Ficarra, P.; J. Chromatogr., B, 838, 56–62, 2006.

90246 1 1 57329 Chilmonczyk, Z.; Ksycinska, H.; Mazgajska, M.; Cybulski, J.; Kaliszan, R.; J. Chromatogr., A, 788, 81–85, 1997.

90246 1 2 110306 Chilmonczyk, Z.; Ksycinska, H.; Lisowska–Kuzmicz, M.; Jonczyk, A.; Mazgajska, M.; Jaronczyk, M.; Aboul–Enein, H.Y.; Anal. Chim. Acta, 536, 7–13, 2005.

90246 1 3 57327 Chilmonczyk, Z.; Ksycinska, H.; Mazgajska, M.; Cybulski, J.; Kaliszan, R.; J. Chromatogr., A, 788, 81–85, 1997.
90246 1 4 110308 Chilmonczyk, Z.; Ksycinska, H.; Lisowska–Kuzmicz, M.; Jonczyk, A.; Mazgajska, M.; Jaronczyk, M.; Aboul–Enein, H.Y.; Anal. Chim. Acta, 536, 7–13, 2005.
90246 1 5 57331 Chilmonczyk, Z.; Ksycinska, H.; Mazgajska, M.; Cybulski, J.; Kaliszan, R.; J. Chromatogr., A, 788, 81–85, 1997.
90246 1 6 110340 Chilmonczyk, Z.; Sienicki, L.; Lozowicka, B;. Lisowska–Kuzmicz, M.; Jonczyk, A.; Aboul–Enein, H.Y.;; Il Farmaco, 60, 439–443, 2005.
90246 1 7 57323 Chilmonczyk, Z.; Ksycinska, H.; Mazgajska, M.; Cybulski, J.; Kaliszan, R.; J. Chromatogr., A, 788, 81–85, 1997.
90246 1 8 110310 Chilmonczyk, Z.; Ksycinska, H.; Lisowska–Kuzmicz, M.; Jonczyk, A.; Mazgajska, M.; Jaronczyk, M.; Aboul–Enein, H.Y.; Anal. Chim. Acta, 536, 7–13, 2005.
90246 1 9 57325 Chilmonczyk, Z.; Ksycinska, H.; Mazgajska, M.; Cybulski, J.; Kaliszan, R.; J. Chromatogr., A, 788, 81–85, 1997.
90246 1 10 110322 Chilmonczyk, Z.; Sienicki, L.; Lozowicka, B;. Lisowska–Kuzmicz, M.; Jonczyk, A.; Aboul–Enein, H.Y.;; Il Farmaco, 60, 439–443, 2005.
90246 2 1 113681 Cirilli, R.; Ferretti, R.; Gallinella, B.; La Torre, F.; La Regina, G.; Silvestri, R.; J. Sep. Sci., 28, 627–634, 2005.
90246 2 2 113641 Cirilli, R.; Ferretti, R.; Gallinella, B.; La Torre, F.; La Regina, G.; Silvestri, R.; J. Sep. Sci., 28, 627–634, 2005.
90246 3 1 1390 Shibata, T.; Ichida, A.; Fukui, Y.; Mori, K.; Int. Symp. Chiral Sep., Guilford, 1989.
90246 3 2 83711 Aboul–Enein, H.Y.; Schmid, M.G.; Tuscher, C.; Gubitz, G.; Laffranchini, M.; Bojarski, J.; J. Liq. Chromatogr. Relat. Technol., 24, 69–77, 2001.
90246 4 1 210534 Jin, L.M.; Xu, X.; Lu, H.J.; Cui, X.; Wojtas, L.; Zhang, X.P.; Angew. Chem., Int. Ed., 52, 5309–5313, 2013.
90246 4 2 210535 Jin, L.M.; Xu, X.; Lu, H.J.; Cui, X.; Wojtas, L.; Zhang, X.P.; Angew. Chem., Int. Ed., 52, 5309–5313, 2013.
90246 5 1 113705 Cirilli, R.; Ferretti, R.; Gallinella, B.; La Torre, F.; La Regina, G.; Silvestri, R.; J. Sep. Sci., 28, 627–634, 2005.
90246 5 2 113713 Cirilli, R.; Ferretti, R.; Gallinella, B.; La Torre, F.; La Regina, G.; Silvestri, R.; J. Sep. Sci., 28, 627–634, 2005.
90246 6 1 31206 Shieh, W.–C.; Cantrell, W.R.; Carlson, J.A.; Tetrahedron Lett., 36, 3797–3800, 1995.
90246 6 2 105119 Ko, D.H.; Kim, K.H.; Ha, D.C.; Org. Lett., 4, 3759–3762, 2002.
90246 6 3 198809 Chen, C.; Wu, K.; Gau, H.; Angew. Chem., Int. Ed., 46, 5373–5376, 2007.
90246 6 4 198811 Chen, C.; Wu, K.; Gau, H.; Angew. Chem., Int. Ed., 46, 5373–5376, 2007.
90246 7 1 117157 Cirilli, R.; Simonelli, A.; Ferretti, R.; Bolasco, A.; Chimenti, P.; Secci , D.; Maccioni, E.; La Torre, F.; J. Chromatogr., A, 1101, 198–203, 2006.
90246 7 2 117191 Cirilli, R.; Simonelli, A.; Ferretti, R.; Bolasco, A.; Chimenti, P.; Secci , D.; Maccioni, E.; La Torre, F.; J. Chromatogr., A, 1101, 198–203, 2006.
90246 8 1 117174 Cirilli, R.; Simonelli, A.; Ferretti, R.; Bolasco, A.; Chimenti, P.; Secci , D.; Maccioni, E.; La Torre, F.; J. Chromatogr., A, 1101, 198–203, 2006.

90246 8 2 117191 Cirilli, R.; Simonelli, A.; Ferretti, R.; Bolasco, A.; Chimenti, P.; Secci , D.; Maccioni, E.; La Torre, F.; J. Chromatogr., A, 1101, 198–203, 2006.

90246 9 1 71404 Saraç, S.; Yarym, M.; Ertan, M.; Anal. Lett., 32, 1245–1254, 1999.

90246 9 2 71398 Saraç, S.; Yarym, M.; Ertan, M.; Anal. Lett., 32, 1245–1254, 1999.

90246 10 1 96670 Andersson, M.E.; Aslan, D.; Clarke, A.; Roeraade, J.; Hagman, G.; J. Chromatogr., A, 1005, 83–101, 2003.

90246 10 2 20048 Soons, P.A.; Roosemalen, M.C.M.; Breimer, D.D.; J. Chromatogr. Biomed. Appl., 528, 343–356, 1990.

90246 11 1 71404 Saraç, S.; Yarym, M.; Ertan, M.; Anal. Lett., 32, 1245–1254, 1999.

90246 11 2 71395 Saraç, S.; Yarym, M.; Ertan, M.; Anal. Lett., 32, 1245–1254, 1999.

90246 12 1 113705 Cirilli, R.; Ferretti, R.; Gallinella, B.; La Torre, F.; La Regina, G.; Silvestri, R.; J. Sep. Sci., 28, 627–634, 2005.

90246 12 2 113665 Cirilli, R.; Ferretti, R.; Gallinella, B.; La Torre, F.; La Regina, G.; Silvestri, R.; J. Sep. Sci., 28, 627–634, 2005.

90246 13 1 64252 Bidlo–Igloy, M.; J. Pharm. Biomed. Anal., 14, 1389–1394, 1996.

90246 13 2 64228 Bidlo–Igloy, M.; J. Pharm. Biomed. Anal., 14, 1389–1394, 1996.

90246 14 1 107390 Cirilli, R.; Ferretti, R.; Gallinella, B.; Turchetto, L.; Bolasco, A.; Secci, D.; Chimenti, P.; Pierini, M.; ... la torre, F.; Chirality, 16, 625–636, 2004.

90246 14 2 107426 Cirilli, R.; Ferretti, R.; Gallinella, B.; Turchetto, L.; Bolasco, A.; Secci, D.; Chimenti, P.; Pierini, M.; ... la torre, F.; Chirality, 16, 625–636, 2004.

90246 15 1 31206 Shieh, W.–C.; Cantrell, W.R.; Carlson, J.A.; Tetrahedron Lett., 36, 3797–3800, 1995.

90246 15 2 31208 Shieh, W.–C.; Cantrell, W.R.; Carlson, J.A.; Tetrahedron Lett., 36, 3797–3800, 1995.

90246 16 1 198809 Chen, C.; Wu, K.; Gau, H.; Angew. Chem., Int. Ed., 46, 5373–5376, 2007.

90246 16 2 198803 Chen, C.; Wu, K.; Gau, H.; Angew. Chem., Int. Ed., 46, 5373–5376, 2007.

90246 16 3 31206 Shieh, W.–C.; Cantrell, W.R.; Carlson, J.A.; Tetrahedron Lett., 36, 3797–3800, 1995.

90246 16 4 31209 Shieh, W.–C.; Cantrell, W.R.; Carlson, J.A.; Tetrahedron Lett., 36, 3797–3800, 1995.

90246 17 1 198809 Chen, C.; Wu, K.; Gau, H.; Angew. Chem., Int. Ed., 46, 5373–5376, 2007.

90246 17 2 67346 Dosa, P.I.; Fu, G.C.; J. Am. Chem. Soc., 120, 445–446, 1998.

90246 18 1 71404 Saraç, S.; Yarym, M.; Ertan, M.; Anal. Lett., 32, 1245–1254, 1999.

90246 18 2 71392 Saraç, S.; Yarym, M.; Ertan, M.; Anal. Lett., 32, 1245–1254, 1999.

90246 19 1 107438 Cirilli, R.; Ferretti, R.; Gallinella, B.; Turchetto, L.; Bolasco, A.; Secci, D.; Chimenti, P.; Pierini, M.; ... la torre, F.; Chirality, 16, 625–636, 2004.

90246 19 2 107364 Cirilli, R.; Ferretti, R.; Gallinella, B.; Turchetto, L.; Bolasco, A.; Secci, D.; Chimenti, P.; Pierini, M.; ... la torre, F.; Chirality, 16, 625–636, 2004.

90246 19 3 107450 Cirilli, R.; Ferretti, R.; Gallinella, B.; Turchetto, L.; Bolasco, A.; Secci, D.; Chimenti, P.; Pierini, M.; ... la torre, F.; Chirality, 16, 625–636, 2004.

90246 19 4 107377 Cirilli, R.; Ferretti, R.; Gallinella, B.; Turchetto, L.; Bolasco, A.; Secci, D.; Chimenti, P.; Pierini, M.; ... la torre, F.; Chirality, 16, 625–636, 2004.

90246 20 1 210534 Jin, L.M.; Xu, X.; Lu, H.J.; Cui, X.; Wojtas, L.; Zhang, X.P.; Angew. Chem., Int. Ed., 52, 5309–5313, 2013.

90246 20 2 210536 Jin, L.M.; Xu, X.; Lu, H.J.; Cui, X.; Wojtas, L.; Zhang, X.P.; Angew. Chem., Int. Ed., 52, 5309–5313, 2013.

90292 1 1 18625 Yang, S.K.; Lu, X.L.; Chirality, 3, 212–219, 1991.

90292 1 2 18630 Yang, S.K.; Lu, X.L.; Chirality, 3, 212–219, 1991.

90292 1 3 25322 Lu, X.–L.; Yang, S.K.; J. Chromatogr., A, 666, 249–2257, 1994.

90292 1 4 25321 Lu, X.–L.; Yang, S.K.; J. Chromatogr., A, 666, 249–2257, 1994.

90292 1 5 25320 Lu, X.–L.; Yang, S.K.; J. Chromatogr., A, 666, 249–2257, 1994.

90292 1 6 25319 Lu, X.–L.; Yang, S.K.; J. Chromatogr., A, 666, 249–2257, 1994.

90292 1 7 25324 Lu, X.–L.; Yang, S.K.; J. Chromatogr., A, 666, 249–2257, 1994.

90292 1 8 25323 Lu, X.–L.; Yang, S.K.; J. Chromatogr., A, 666, 249–2257, 1994.

90292 1 9 8937 Pirkle, W.H.; Tsipouras, A.; J. Chromatogr., 291, 291–298, 1984.

90292 1 10 8949 Pirkle, W.H.; Tsipouras, A.; J. Chromatogr., 291, 291–298, 1984.

90292 1 11 25328 Lu, X.–L.; Yang, S.K.; J. Chromatogr., A, 666, 249–2257, 1994.

90292 1 12 25327 Lu, X.–L.; Yang, S.K.; J. Chromatogr., A, 666, 249–2257, 1994.

90292 1 13 25326 Lu, X.–L.; Yang, S.K.; J. Chromatogr., A, 666, 249–2257, 1994.

90292 1 14 25325 Lu, X.–L.; Yang, S.K.; J. Chromatogr., A, 666, 249–2257, 1994.

90292 1 15 8964 Pirkle, W.H.; Tsipouras, A.; J. Chromatogr., 291, 291–298, 1984.

90292 1 16 14161 Azzolina, O.; Ghislandi, V.; Vercesi, D.; Farmaco, 45(6), 603–615, 1990.

90292 1 17 8940 Pirkle, W.H.; Tsipouras, A.; J. Chromatogr., 291, 291–298, 1984.

90292 1 18 8950 Pirkle, W.H.; Tsipouras, A.; J. Chromatogr., 291, 291–298, 1984.

90292 2 1 8944 Pirkle, W.H.; Tsipouras, A.; J. Chromatogr., 291, 291–298, 1984.

90292 2 2 136938 Forjan, DM; Kontrec, D; Vinkovic, V; Chirality, 18, 857–869, 2006.

90292 3 1 5312 Pirkle, W.H.; Sowin, T.J.; J. Org. Chem., 52(14), 3011–3017, 1987.

90292 3 2 12355 Pirkle, W.H.; Sowin, T.J.; J. Chromatogr., 387, 313–321, 1987.

90292 4 1 9104 Pirkle, W.H.; Pochapsky, T.C.; Mahler, G.S.; Field, R.E.; J. Chromatogr., 348, 89–96, 1985.

90292 4 2 9116 Pirkle, W.H.; Pochapsky, T.C.; Mahler, G.S.; Field, R.E.; J. Chromatogr., 348, 89–96, 1985.

90292 4 3 9105 Pirkle, W.H.; Pochapsky, T.C.; Mahler, G.S.; Field, R.E.; J. Chromatogr., 348, 89–96, 1985.

90292 4 4 9117 Pirkle, W.H.; Pochapsky, T.C.; Mahler, G.S.; Field, R.E.; J. Chromatogr., 348, 89–96, 1985.

90292 4 5 9103 Pirkle, W.H.; Pochapsky, T.C.; Mahler, G.S.; Field, R.E.; J. Chromatogr., 348, 89–96, 1985.

90292 4 6 9115 Pirkle, W.H.; Pochapsky, T.C.; Mahler, G.S.; Field, R.E.; J. Chromatogr., 348, 89–96, 1985.

90292 5 1 8944 Pirkle, W.H.; Tsipouras, A.; J. Chromatogr., 291, 291–298, 1984.

90292 5 2 136942 Forjan, DM; Kontrec, D; Vinkovic, V; Chirality, 18, 857–869, 2006.

90589 1 1 111823 Ryu, J.S.; Marks, T.J.; McDonald, F.E.; J. Org. Chem., 69, 1038–1052, 2004.

90589 1 2 112968 Hong, S.; Tian, S.; Metz, M.V.; Marks, T.J.; J. Am. Chem. Soc., 125, 14768–14783, 2003.

90589 1 3 112965 Hong, S.; Tian, S.; Metz, M.V.; Marks, T.J.; J. Am. Chem. Soc., 125, 14768–14783, 2003.

90589 1 4 43515 Hyun, M.H.; Jin, J.S.; Lee, W.; Bull. Korean Chem. Soc., 18, 336–339, 1997.

90589 1 5 111824 Ryu, J.S.; Marks, T.J.; McDonald, F.E.; J. Org. Chem., 69, 1038–1052, 2004.

90589 1 6 43522 Hyun, M.H.; Jin, J.S.; Lee, W.; Bull. Korean Chem. Soc., 18, 336–339, 1997.

90589 1 7 112969 Hong, S.; Tian, S.; Metz, M.V.; Marks, T.J.; J. Am. Chem. Soc., 125, 14768–14783, 2003.

90589 1 8 43521 Hyun, M.H.; Jin, J.S.; Lee, W.; Bull. Korean Chem. Soc., 18, 336–339, 1997.

90589 1 9 112965 Hong, S.; Tian, S.; Metz, M.V.; Marks, T.J.; J. Am. Chem. Soc., 125, 14768–14783, 2003.

90589 1 10 43517 Hyun, M.H.; Jin, J.S.; Lee, W.; Bull. Korean Chem. Soc., 18, 336–339, 1997.

90589 1 11 112969 Hong, S.; Tian, S.; Metz, M.V.; Marks, T.J.; J. Am. Chem. Soc., 125, 14768–14783, 2003.

90589 1 12 43524 Hyun, M.H.; Jin, J.S.; Lee, W.; Bull. Korean Chem. Soc., 18, 336–339, 1997.

90589 2 1 111822 Ryu, J.S.; Marks, T.J.; McDonald, F.E.; J. Org. Chem., 69, 1038–1052, 2004.

90589 2 2 43517 Hyun, M.H.; Jin, J.S.; Lee, W.; Bull. Korean Chem. Soc., 18, 336–339, 1997.

90589 2 3 111824 Ryu, J.S.; Marks, T.J.; McDonald, F.E.; J. Org. Chem., 69, 1038–1052, 2004.

90589 2 4 43524 Hyun, M.H.; Jin, J.S.; Lee, W.; Bull. Korean Chem. Soc., 18, 336–339, 1997.

90589 3 1 148538 Dungelova, J.; Lehotay, J.; Krupcik, J.; Cizmarik, J.; Armstrong, D.W.; J. Sep. Sci., 27, 983–990, 2004.

90589 3 2 148568 Dungelova, J.; Lehotay, J.; Krupcik, J.; Cizmarik, J.; Armstrong, D.W.; J. Sep. Sci., 27, 983–990, 2004.

90589 4 1 148538 Dungelova, J.; Lehotay, J.; Krupcik, J.; Cizmarik, J.; Armstrong, D.W.; J. Sep. Sci., 27, 983–990, 2004.

90589 4 2 148574 Dungelova, J.; Lehotay, J.; Krupcik, J.; Cizmarik, J.; Armstrong, D.W.; J. Sep. Sci., 27, 983–990, 2004.

90589 5 1 111823 Ryu, J.S.; Marks, T.J.; McDonald, F.E.; J. Org. Chem., 69, 1038–1052, 2004.

90589 5 2 43517 Hyun, M.H.; Jin, J.S.; Lee, W.; Bull. Korean Chem. Soc., 18, 336–339, 1997.

90589 6 1 111822 Ryu, J.S.; Marks, T.J.; McDonald, F.E.; J. Org. Chem., 69, 1038–1052, 2004.

90589 6 2 43524 Hyun, M.H.; Jin, J.S.; Lee, W.; Bull. Korean Chem. Soc., 18, 336–339, 1997.

90589 7 1 111823 Ryu, J.S.; Marks, T.J.; McDonald, F.E.; J. Org. Chem., 69, 1038–1052, 2004.

90589 7 2 43524 Hyun, M.H.; Jin, J.S.; Lee, W.; Bull. Korean Chem. Soc., 18, 336–339, 1997.

90589 8 1 112965 Hong, S.; Tian, S.; Metz, M.V.; Marks, T.J.; J. Am. Chem. Soc., 125, 14768–14783, 2003.

90589 8 2 43524 Hyun, M.H.; Jin, J.S.; Lee, W.; Bull. Korean Chem. Soc., 18, 336–339, 1997.

90589 9 1 78057 Hyun, M.H.; Koo, H.J.; Lee, G.S.; Han, S.C; Enantiomer, 5, 499–503, 2000.

90589 9 2 78051 Hyun, M.H.; Koo, H.J.; Lee, G.S.; Han, S.C; Enantiomer, 5, 499–503, 2000.

90589 10 1 57788 Lee, W.; Bull. Korean Chem. Soc., 19(7), 715–717, 1998.

90589 10 2 57787 Lee, W.; Bull. Korean Chem. Soc., 19(7), 715–717, 1998.

90589 10 3 57791 Lee, W.; Bull. Korean Chem. Soc., 19(7), 715–717, 1998.

90589 10 4 57790 Lee, W.; Bull. Korean Chem. Soc., 19(7), 715–717, 1998.

90589 11 1 78058 Hyun, M.H.; Koo, H.J.; Lee, G.S.; Han, S.C; Enantiomer, 5, 499–503, 2000.

90589 11 2 78059 Hyun, M.H.; Koo, H.J.; Lee, G.S.; Han, S.C; Enantiomer, 5, 499–503, 2000.

90589 12 1 107901 Job, G.E.; Shvets, A.; Pirkle, W.H.; Kuwahara, S.; Kosaka, M.; Kasai, Y.; Taji, H.; Fujita, K.; Watanabe, M.; Harada, N.;; J. Chromatogr., A, 1055, 41–53, 2004.

90589 12 2 107899 Job, G.E.; Shvets, A.; Pirkle, W.H.; Kuwahara, S.; Kosaka, M.; Kasai, Y.; Taji, H.; Fujita, K.; Watanabe, M.; Harada, N.;; J. Chromatogr., A, 1055, 41–53, 2004.

90589 12 3 107904 Job, G.E.; Shvets, A.; Pirkle, W.H.; Kuwahara, S.; Kosaka, M.; Kasai, Y.; Taji, H.; Fujita, K.; Watanabe, M.; Harada, N.;; J. Chromatogr., A, 1055, 41–53, 2004.

90589 12 4 107902 Job, G.E.; Shvets, A.; Pirkle, W.H.; Kuwahara, S.; Kosaka, M.; Kasai, Y.; Taji, H.; Fujita, K.; Watanabe, M.; Harada, N.;; J. Chromatogr., A, 1055, 41–53, 2004.

90589 12 5 107898 Job, G.E.; Shvets, A.; Pirkle, W.H.; Kuwahara, S.; Kosaka, M.; Kasai, Y.; Taji, H.; Fujita, K.; Watanabe, M.; Harada, N.;; J. Chromatogr., A, 1055, 41–53, 2004.

90589 12 6 107896 Job, G.E.; Shvets, A.; Pirkle, W.H.; Kuwahara, S.; Kosaka, M.; Kasai, Y.; Taji, H.; Fujita, K.; Watanabe, M.; Harada, N.;; J. Chromatogr., A, 1055, 41–53, 2004.

90589 12 7 107907 Job, G.E.; Shvets, A.; Pirkle, W.H.; Kuwahara, S.; Kosaka, M.; Kasai, Y.; Taji, H.; Fujita, K.; Watanabe, M.; Harada, N.;; J. Chromatogr., A, 1055, 41–53, 2004.

90589 12 8 107905 Job, G.E.; Shvets, A.; Pirkle, W.H.; Kuwahara, S.; Kosaka, M.; Kasai, Y.; Taji, H.; Fujita, K.; Watanabe, M.; Harada, N.;; J. Chromatogr., A, 1055, 41–53, 2004.

90589 13 1 57776 Lee, W.; Bull. Korean Chem. Soc., 19(7), 715–717, 1998.

90589 13 2 57775 Lee, W.; Bull. Korean Chem. Soc., 19(7), 715–717, 1998.

90589 13 3 57793 Lee, W.; Bull. Korean Chem. Soc., 19(7), 715–717, 1998.

90589 13 4 57792 Lee, W.; Bull. Korean Chem. Soc., 19(7), 715–717, 1998.

90589 14 1 111822 Ryu, J.S.; Marks, T.J.; McDonald, F.E.; J. Org. Chem., 69, 1038–1052, 2004.

90589 14 2 43522 Hyun, M.H.; Jin, J.S.; Lee, W.; Bull. Korean Chem. Soc., 18, 336–339, 1997.

90589 14 3 111823 Ryu, J.S.; Marks, T.J.; McDonald, F.E.; J. Org. Chem., 69, 1038–1052, 2004.

90589 14 4 112970 Hong, S.; Tian, S.; Metz, M.V.; Marks, T.J.; J. Am. Chem. Soc., 125, 14768–14783, 2003.

90704 1 1 41899 Kleidernigg, O.P.; Kappe, C.O.; Tetrahedron: Asymmetry, 8, 2057–2067, 1997.

90704 1 2 41917 Kleidernigg, O.P.; Kappe, C.O.; Tetrahedron: Asymmetry, 8, 2057–2067, 1997.

90704 2 1 41959 Kleidernigg, O.P.; Kappe, C.O.; Tetrahedron: Asymmetry, 8, 2057–2067, 1997.

90704 2 2 41923 Kleidernigg, O.P.; Kappe, C.O.; Tetrahedron: Asymmetry, 8, 2057–2067, 1997.

90704 3 1 79426 Wang, A.X.; Lee, J.T.; Beesley, T.E.; LC–GC, 18, 626–639, 2000.

90704 3 2 86406 Lee, J.T.; Beesley, T.E.; Pittcon'2001 New Orleans, 2001.

90704 4 1 79426 Wang, A.X.; Lee, J.T.; Beesley, T.E.; LC–GC, 18, 626–639, 2000.

90704 4 2 27987 Armstrong, D.W.; Tang, Y.; Chen, S.; Zhou, Y.; Bagwill, C.; Chen, J.–R.; Anal. Chem., 66, 1473–1484, 1994.

90704 5 1 41959 Kleidernigg, O.P.; Kappe, C.O.; Tetrahedron: Asymmetry, 8, 2057–2067, 1997.

90704 5 2 41905 Kleidernigg, O.P.; Kappe, C.O.; Tetrahedron: Asymmetry, 8, 2057–2067, 1997.

90704 6 1 41959 Kleidernigg, O.P.; Kappe, C.O.; Tetrahedron: Asymmetry, 8, 2057–2067, 1997.

90704 6 2 41911 Kleidernigg, O.P.; Kappe, C.O.; Tetrahedron: Asymmetry, 8, 2057–2067, 1997.

90704 7 1 79426 Wang, A.X.; Lee, J.T.; Beesley, T.E.; LC–GC, 18, 626–639, 2000.

90704 7 2 48885 Berthod, A.; Nair, U. B.; Bagwill, C.; Armstrong, D. W.; Talanta, 43, 1767–1782, 1996.

90704 8 1 164304 Evans, C.G.; Gestwicki, J.E.; Org. Lett. , 11, 2957–2959, 2009.

90704 8 2 164296 Evans, C.G.; Gestwicki, J.E.; Org. Lett. , 11, 2957–2959, 2009.

90704 9 1 41899 Kleidernigg, O.P.; Kappe, C.O.; Tetrahedron: Asymmetry, 8, 2057–2067, 1997.

90704 9 2 42013 Kleidernigg, O.P.; Kappe, C.O.; Tetrahedron: Asymmetry, 8, 2057–2067, 1997.

90704 10 1 164304 Evans, C.G.; Gestwicki, J.E.; Org. Lett. , 11, 2957–2959, 2009.

90704 10 2 164295 Evans, C.G.; Gestwicki, J.E.; Org. Lett. , 11, 2957–2959, 2009.

90704 11 1 41959 Kleidernigg, O.P.; Kappe, C.O.; Tetrahedron: Asymmetry, 8, 2057–2067, 1997.

90704 11 2 42013 Kleidernigg, O.P.; Kappe, C.O.; Tetrahedron: Asymmetry, 8, 2057–2067, 1997.

90704 12 1 41899 Kleidernigg, O.P.; Kappe, C.O.; Tetrahedron: Asymmetry, 8, 2057–2067, 1997.

90704 12 2 41923 Kleidernigg, O.P.; Kappe, C.O.; Tetrahedron: Asymmetry, 8, 2057–2067, 1997.

90704 12 3 41905 Kleidernigg, O.P.; Kappe, C.O.; Tetrahedron: Asymmetry, 8, 2057–2067, 1997.

90704 12 4 41929 Kleidernigg, O.P.; Kappe, C.O.; Tetrahedron: Asymmetry, 8, 2057–2067, 1997.

90704 13 1 41899 Kleidernigg, O.P.; Kappe, C.O.; Tetrahedron: Asymmetry, 8, 2057–2067, 1997.

90704 13 2 41935 Kleidernigg, O.P.; Kappe, C.O.; Tetrahedron: Asymmetry, 8, 2057–2067, 1997.

90704 13 3 41941 Kleidernigg, O.P.; Kappe, C.O.; Tetrahedron: Asymmetry, 8, 2057–2067, 1997.

90704 13 4 41953 Kleidernigg, O.P.; Kappe, C.O.; Tetrahedron: Asymmetry, 8, 2057–2067, 1997.

90704 14 1 83647 Jandera, P.; Skavrada, M.; Klemmova, K.; Backovska, V.; Guiochon, G.; J. Chromatogr., A, 917, 123–133, 2001.

90704 14 2 114734 Ghassempour, A.; Abdollahpour, A.; Tabar–Heydar, K.; Nabid, M.R.; Mansouri, S.; Aboul–Enein, H.Y.; Chromatographia, 61, 151–155, 2005.

90704 15 1 41899 Kleidernigg, O.P.; Kappe, C.O.; Tetrahedron: Asymmetry, 8, 2057–2067, 1997.

90704 15 2 41905 Kleidernigg, O.P.; Kappe, C.O.; Tetrahedron: Asymmetry, 8, 2057–2067, 1997.

90704 15 3 41941 Kleidernigg, O.P.; Kappe, C.O.; Tetrahedron: Asymmetry, 8, 2057–2067, 1997.
90704 15 4 41947 Kleidernigg, O.P.; Kappe, C.O.; Tetrahedron: Asymmetry, 8, 2057–2067, 1997.
90704 15 5 41923 Kleidernigg, O.P.; Kappe, C.O.; Tetrahedron: Asymmetry, 8, 2057–2067, 1997.
90704 15 6 41929 Kleidernigg, O.P.; Kappe, C.O.; Tetrahedron: Asymmetry, 8, 2057–2067, 1997.
90704 15 7 41911 Kleidernigg, O.P.; Kappe, C.O.; Tetrahedron: Asymmetry, 8, 2057–2067, 1997.
90704 15 8 41917 Kleidernigg, O.P.; Kappe, C.O.; Tetrahedron: Asymmetry, 8, 2057–2067, 1997.
90704 16 1 41899 Kleidernigg, O.P.; Kappe, C.O.; Tetrahedron: Asymmetry, 8, 2057–2067, 1997.
90704 16 2 41911 Kleidernigg, O.P.; Kappe, C.O.; Tetrahedron: Asymmetry, 8, 2057–2067, 1997.
90704 16 3 41905 Kleidernigg, O.P.; Kappe, C.O.; Tetrahedron: Asymmetry, 8, 2057–2067, 1997.
90704 16 4 41917 Kleidernigg, O.P.; Kappe, C.O.; Tetrahedron: Asymmetry, 8, 2057–2067, 1997.
90879 1 1 124198 Chen, S.; Chromatographia, 63, 97–102, 2006.
90879 1 2 124228 Chen, S.; Chromatographia, 63, 97–102, 2006.
90879 1 3 124200 Chen, S.; Chromatographia, 63, 97–102, 2006.
90879 1 4 124230 Chen, S.; Chromatographia, 63, 97–102, 2006.
90879 1 5 124199 Chen, S.; Chromatographia, 63, 97–102, 2006.
90879 1 6 124229 Chen, S.; Chromatographia, 63, 97–102, 2006.
90879 1 7 124197 Chen, S.; Chromatographia, 63, 97–102, 2006.
90879 1 8 124227 Chen, S.; Chromatographia, 63, 97–102, 2006.
90879 2 1 42347 Armstrong, D.; Liu, Y.; Ekborgott, K.H.; Chirality, 7, 474–497, 1995.
90879 2 2 99902 Chen, S.; Ward, T.; Chirality, 16, 318–330, 2004.
90879 2 3 42339 Armstrong, D.; Liu, Y.; Ekborgott, K.H.; Chirality, 7, 474–497, 1995.
90879 2 4 99891 Chen, S.; Ward, T.; Chirality, 16, 318–330, 2004.
90879 2 5 42341 Armstrong, D.; Liu, Y.; Ekborgott, K.H.; Chirality, 7, 474–497, 1995.
90879 2 6 99919 Chen, S.; Ward, T.; Chirality, 16, 318–330, 2004.
90879 3 1 124149 Chen, S.; Chromatographia, 63, 97–102, 2006.
90879 3 2 124278 Chen, S.; Chromatographia, 63, 97–102, 2006.
90879 3 3 124150 Chen, S.; Chromatographia, 63, 97–102, 2006.
90879 3 4 124279 Chen, S.; Chromatographia, 63, 97–102, 2006.
90879 4 1 130487 Chen, S.; Biomed. Chromatogr., 20, 718–728, 2006.
90879 4 2 130484 Chen, S.; Biomed. Chromatogr., 20, 718–728, 2006.
90879 4 3 130541 Chen, S.; Biomed. Chromatogr., 20, 718–728, 2006.
90879 4 4 130538 Chen, S.; Biomed. Chromatogr., 20, 718–728, 2006.
90879 4 5 130549 Chen, S.; Biomed. Chromatogr., 20, 718–728, 2006.

90879 4 6 130547 Chen, S.; Biomed. Chromatogr., 20, 718–728, 2006.

90879 4 7 130525 Chen, S.; Biomed. Chromatogr., 20, 718–728, 2006.

90879 4 8 130522 Chen, S.; Biomed. Chromatogr., 20, 718–728, 2006.

90879 4 9 130533 Chen, S.; Biomed. Chromatogr., 20, 718–728, 2006.

90879 4 10 130531 Chen, S.; Biomed. Chromatogr., 20, 718–728, 2006.

90879 4 11 130555 Chen, S.; Biomed. Chromatogr., 20, 718–728, 2006.

90879 4 12 130553 Chen, S.; Biomed. Chromatogr., 20, 718–728, 2006.

90879 5 1 130463 Chen, S.; Biomed. Chromatogr., 20, 718–728, 2006.

90879 5 2 130613 Chen, S.; Biomed. Chromatogr., 20, 718–728, 2006.

90879 5 3 130464 Chen, S.; Biomed. Chromatogr., 20, 718–728, 2006.

90879 5 4 130614 Chen, S.; Biomed. Chromatogr., 20, 718–728, 2006.

90879 6 1 124149 Chen, S.; Chromatographia, 63, 97–102, 2006.

90879 6 2 124297 Chen, S.; Chromatographia, 63, 97–102, 2006.

90879 6 3 124150 Chen, S.; Chromatographia, 63, 97–102, 2006.

90879 6 4 124298 Chen, S.; Chromatographia, 63, 97–102, 2006.

90879 7 1 42339 Armstrong, D.; Liu, Y.; Ekborgott, K.H.; Chirality, 7, 474–497, 1995.

90879 7 2 99894 Chen, S.; Ward, T.; Chirality, 16, 318–330, 2004.

90879 8 1 130464 Chen, S.; Biomed. Chromatogr., 20, 718–728, 2006.

90879 8 2 130594 Chen, S.; Biomed. Chromatogr., 20, 718–728, 2006.

90879 9 1 135510 Dalmolen, J.; Tiemersma–Wegman, T.D.; Nieuwenhuijzen, J.W.; van der Sluis, M.; vzn Echten, E.; Vries, T.R.; Kaptein, B.; Broxterman, Q.B.; Kellogg, R.M.; Chem. Eur. J., 11, 5619–5624, 2005.

90879 9 2 117044 Boesten, J.M.M.; Berkheij, M. ; Schoemaker, H.E.; Hiemstra, H.; Duchateau, A.L.L.; J. Chromatogr., A, 1108, 26–30, 2006.

90879 10 1 41900 Kleidernigg, O.P.; Kappe, C.O.; Tetrahedron: Asymmetry, 8, 2057–2067, 1997.

90879 10 2 41906 Kleidernigg, O.P.; Kappe, C.O.; Tetrahedron: Asymmetry, 8, 2057–2067, 1997.

90879 10 3 41942 Kleidernigg, O.P.; Kappe, C.O.; Tetrahedron: Asymmetry, 8, 2057–2067, 1997.

90879 10 4 41948 Kleidernigg, O.P.; Kappe, C.O.; Tetrahedron: Asymmetry, 8, 2057–2067, 1997.

90879 10 5 41924 Kleidernigg, O.P.; Kappe, C.O.; Tetrahedron: Asymmetry, 8, 2057–2067, 1997.

90879 10 6 41930 Kleidernigg, O.P.; Kappe, C.O.; Tetrahedron: Asymmetry, 8, 2057–2067, 1997.

90879 10 7 41912 Kleidernigg, O.P.; Kappe, C.O.; Tetrahedron: Asymmetry, 8, 2057–2067, 1997.

90879 10 8 41918 Kleidernigg, O.P.; Kappe, C.O.; Tetrahedron: Asymmetry, 8, 2057–2067, 1997.

90879 11 1 42339 Armstrong, D.; Liu, Y.; Ekborgott, K.H.; Chirality, 7, 474–497, 1995.

90879 11 2 100088 Chen, S.; Ward, T.; Chirality, 16, 318–330, 2004.

90879 12 1 42339 Armstrong, D.; Liu, Y.; Ekborgott, K.H.; Chirality, 7, 474–497, 1995.

90879 12 2 99896 Chen, S.; Ward, T.; Chirality, 16, 318–330, 2004.
90879 13 1 42444 Armstrong, D.; Liu, Y.; Ekborgott, K.H.; Chirality, 7, 474–497, 1995.
90879 13 2 128806 Xiao, T.L.; Tesarova, E.; Anderson, J.L.; Egger, M.; Armstrong, D.W.; J. Sep. Sci., 29, 429–445, 2006.
90879 14 1 42339 Armstrong, D.; Liu, Y.; Ekborgott, K.H.; Chirality, 7, 474–497, 1995.
90879 14 2 99893 Chen, S.; Ward, T.; Chirality, 16, 318–330, 2004.
90879 14 3 42347 Armstrong, D.; Liu, Y.; Ekborgott, K.H.; Chirality, 7, 474–497, 1995.
90879 14 4 99904 Chen, S.; Ward, T.; Chirality, 16, 318–330, 2004.
90879 15 1 124159 Chen, S.; Chromatographia, 63, 97–102, 2006.
90879 15 2 124278 Chen, S.; Chromatographia, 63, 97–102, 2006.
90879 15 3 124160 Chen, S.; Chromatographia, 63, 97–102, 2006.
90879 15 4 124279 Chen, S.; Chromatographia, 63, 97–102, 2006.
90879 16 1 130464 Chen, S.; Biomed. Chromatogr., 20, 718–728, 2006.
90879 16 2 130406 Chen, S.; Biomed. Chromatogr., 20, 718–728, 2006.
90879 17 1 130590 Chen, S.; Biomed. Chromatogr., 20, 718–728, 2006.
90879 17 2 130594 Chen, S.; Biomed. Chromatogr., 20, 718–728, 2006.
90879 18 1 130596 Chen, S.; Biomed. Chromatogr., 20, 718–728, 2006.
90879 18 2 130598 Chen, S.; Biomed. Chromatogr., 20, 718–728, 2006.
90879 19 1 130463 Chen, S.; Biomed. Chromatogr., 20, 718–728, 2006.
90879 19 2 130593 Chen, S.; Biomed. Chromatogr., 20, 718–728, 2006.
90879 20 1 130561 Chen, S.; Biomed. Chromatogr., 20, 718–728, 2006.
90879 20 2 130592 Chen, S.; Biomed. Chromatogr., 20, 718–728, 2006.
91119 1 1 147344 Schmid, M.G.; Holbling, M.; Schnedlitz, N.; Gubitz, G.; J. Biochem. Biophys. Methods, 61, 1–10, 2004.
91119 1 2 128921 Xiao, T.L.; Tesarova, E.; Anderson, J.L.; Egger, M.; Armstrong, D.W.; J. Sep. Sci., 29, 429–445, 2006.
91119 2 1 69233 Berthod, A.; Chen, X.; Kullman, J.P.; Armstrong, D.W.; Gasparrini, F.; D'Acquarica, I.; Villani, C.; Anal. Chem., 72, 1767–1780, 2000.
91119 2 2 98697 Peter, A.; Arki, A.; Tourwe, D.; Forro, E.; Fulop, F.; Armstrong, D.W.; J. Chromatogr., A, 1031, 159–170, 2004.
91119 3 1 69328 Berthod, A.; Chen, X.; Kullman, J.P.; Armstrong, D.W.; Gasparrini, F.; D'Acquarica, I.; Villani, C.; Anal. Chem., 72, 1767–1780, 2000.
91119 3 2 128661 Xiao, T.L.; Tesarova, E.; Anderson, J.L.; Egger, M.; Armstrong, D.W.; J. Sep. Sci., 29, 429–445, 2006.
91119 4 1 69328 Berthod, A.; Chen, X.; Kullman, J.P.; Armstrong, D.W.; Gasparrini, F.; D'Acquarica, I.; Villani, C.; Anal. Chem., 72, 1767–1780, 2000.
91119 4 2 128667 Xiao, T.L.; Tesarova, E.; Anderson, J.L.; Egger, M.; Armstrong, D.W.; J. Sep. Sci., 29, 429–445, 2006.
91119 5 1 69328 Berthod, A.; Chen, X.; Kullman, J.P.; Armstrong, D.W.; Gasparrini, F.; D'Acquarica, I.; Villani, C.; Anal. Chem., 72, 1767–1780, 2000.
91119 5 2 128685 Xiao, T.L.; Tesarova, E.; Anderson, J.L.; Egger, M.; Armstrong, D.W.; J. Sep. Sci., 29, 429–445, 2006.

91119 6 1 147350 Schmid, M.G.; Holbling, M.; Schnedlitz, N.; Gubitz, G.; J. Biochem. Biophys. Methods, 61, 1–10, 2004.

91119 6 2 128915 Xiao, T.L.; Tesarova, E.; Anderson, J.L.; Egger, M.; Armstrong, D.W.; J. Sep. Sci., 29, 429–445, 2006.

91423 1 1 168845 Liu,X.–W.; Yuan, M.; Ji, H.; Journal of Chinese Pharmaceutical Sciences, 19, 293–299, 2010.

91423 1 2 168788 Liu,X.–W.; Yuan, M.; Ji, H.; Journal of Chinese Pharmaceutical Sciences, 19, 293–299, 2010.

91423 2 1 168845 Liu,X.–W.; Yuan, M.; Ji, H.; Journal of Chinese Pharmaceutical Sciences, 19, 293–299, 2010.

91423 2 2 168797 Liu,X.–W.; Yuan, M.; Ji, H.; Journal of Chinese Pharmaceutical Sciences, 19, 293–299, 2010.

91423 3 1 168845 Liu,X.–W.; Yuan, M.; Ji, H.; Journal of Chinese Pharmaceutical Sciences, 19, 293–299, 2010.

91423 3 2 168829 Liu,X.–W.; Yuan, M.; Ji, H.; Journal of Chinese Pharmaceutical Sciences, 19, 293–299, 2010.

91423 4 1 171775 CHIRAL TECHNOLOGIES EUROPE; Application Guide Software, 2010.

91423 4 2 216325 Zhang, T.; Franco, P.; Nguyen, D.; Hamasaki, R.; Miyamoto, S.; Ohnishi, A.; Murakami, T.; J. Chromatogr., A, 1269, 178–188, 2012.

91423 5 1 168845 Liu,X.–W.; Yuan, M.; Ji, H.; Journal of Chinese Pharmaceutical Sciences, 19, 293–299, 2010.

91423 5 2 168805 Liu,X.–W.; Yuan, M.; Ji, H.; Journal of Chinese Pharmaceutical Sciences, 19, 293–299, 2010.

91423 6 1 168845 Liu,X.–W.; Yuan, M.; Ji, H.; Journal of Chinese Pharmaceutical Sciences, 19, 293–299, 2010.

91423 6 2 168813 Liu,X.–W.; Yuan, M.; Ji, H.; Journal of Chinese Pharmaceutical Sciences, 19, 293–299, 2010.

91423 7 1 212400 Wang, Z.; Zhang, Z.L.; Yao, Q.; Liu, X.H.; Cai, Y.F.; Lin, L.L.; Feng, X.M.; Chemistry–A European Journal, 19, 8591–8596, 2013.

91423 7 2 212411 Wang, Z.; Zhang, Z.L.; Yao, Q.; Liu, X.H.; Cai, Y.F.; Lin, L.L.; Feng, X.M.; Chemistry–A European Journal, 19, 8591–8596, 2013.

91423 8 1 168861 Liu,X.–W.; Yuan, M.; Ji, H.; Journal of Chinese Pharmaceutical Sciences, 19, 293–299, 2010.

91423 8 2 168788 Liu,X.–W.; Yuan, M.; Ji, H.; Journal of Chinese Pharmaceutical Sciences, 19, 293–299, 2010.

91423 9 1 168861 Liu,X.–W.; Yuan, M.; Ji, H.; Journal of Chinese Pharmaceutical Sciences, 19, 293–299, 2010.

91423 9 2 168797 Liu,X.–W.; Yuan, M.; Ji, H.; Journal of Chinese Pharmaceutical Sciences, 19, 293–299, 2010.

91423 10 1 168853 Liu,X.–W.; Yuan, M.; Ji, H.; Journal of Chinese Pharmaceutical Sciences, 19, 293–299, 2010.

91423 10 2 168788 Liu,X.–W.; Yuan, M.; Ji, H.; Journal of Chinese Pharmaceutical Sciences, 19, 293–299, 2010.

91423 11 1 168853 Liu,X.–W.; Yuan, M.; Ji, H.; Journal of Chinese Pharmaceutical Sciences, 19, 293–299, 2010.
91423 11 2 168797 Liu,X.–W.; Yuan, M.; Ji, H.; Journal of Chinese Pharmaceutical Sciences, 19, 293–299, 2010.

4. Computational Modeling

Our general approach for conformational sampling including the detailed computational workflow, has been published elsewhere [1]. A subset of the details of the computational methodology is provided here. Conformers of each structure were geometry optimized at the B3LYP/6–31G** level and stationary points were confirmed by performing frequency calculations (scaled by 0.98) [2–11]. All calculations were performed using Gaussian 09 [11]. Conformations contributing to the *in vacuo* Boltzmann distribution were calculated and used to interpret conformational differences induced by various substitution.

1. Sherer, E.C.; Lee, C.H.; Shpungin, J.; Cuff, J.F.; Da, C.X.; Ball, R.R.; Crespo, A.; Gong, X.Y.; Welch, C.J. Systematic Approach to Conformational Sampling for Assigning Absolute Configuration Using Vibrational Circular Dichroism. *J. Med. Chem.* **2014**, *57*, 477–494.
2. Petersson, G.A.; Allaham, M.A. A complete basis set model chemistry. II. Open-shell systems and the total energies of the first-row atoms. *J. Chem. Phys.* **1991**, *94*, 6081–6090.
3. Petersson, G.A.; Bennett, A.; Tensfeldt, T.G.; Allaham, M.A.; Shirley,W.A.; Mantzaris, J. A complete basis set model chemistry. I. The total energies of closed–shell atoms and hydrides of the first–row elements. *J. Chem. Phys.,* **1988**, *89*, 2193–2218.
4. Rassolov, V.A.; Pople, J.A.; Ratner, M.A.; Windus, T.L. 6–31G* basis set for atoms K through Zn. *J. Chem. Phys.* **1998**, *109*, 1223–1229.
5. Rassolov, V.A.; Ratner, M.A.; Pople, J.A.; Redfern, P.C.; Curtiss, L.A. 6–31G* basis set for third–row atoms. *J. Comput. Chem.* **2001**, *22*, 976–984.
6. Francl, M.M.; Pietro, W.K.; Hehre, W.J.; Binkley, J.S.; Gordon, M.S.; Defrees, D.J.; Pople, J.A. Self-consistent molecular orbital methods. XXIII. A polarization-type basis set for second-row elements. *J. Chem. Phys.* **1982**, *77*, 3654–3665.
7. Hehre, W.J.; Ditchfield, R.; Pople, J.A. Self—Consistent Molecular Orbital Methods. XII. Further Extensions of Gaussian—Type Basis Sets for Use in Molecular Orbital Studies of Organic Molecules. *J. Chem. Phys.* **1972**, *56*, 2257–2261.
8. Becke, A.D. Density-functional thermochemistry. III. The role of exact exchange. *J. Chem. Phys.* **1993**, *98*, 5648–5652.
9. Lee, C.T.; Yang, W.T.; Parr, R.G. Development of the Colle–Salvetti correlation–energy formula into a functional of the electron density. *Phys. Rev. B* **1988**, *37*, 785–789.
10. Miehlich, B.; Savin, A.; Stoll, H.; Preuss, H. Results obtained with the correlation energy density functionals of becke and Lee, Yang and Parr. *Chem. Phys. Lett.* **1989**, *157*, 200–206.
11. Frisch, M.J.; Trucks, G.W.; Schlegel, H.B.; Scuseria, G.E.; Robb, M.A.; Cheeseman, J.R.; Scalmani, G.; Barone, V.; Mennucci, B.; Petersson, G.A.; et al. Gaussian 09, Revision A.02. Gaussian, Inc: Wallingford, CT, USA; 2009.

5. Minimized Conformations for Nitro and Methyl ketone Analogs from MMP Shown in Figure 2 from manuscript

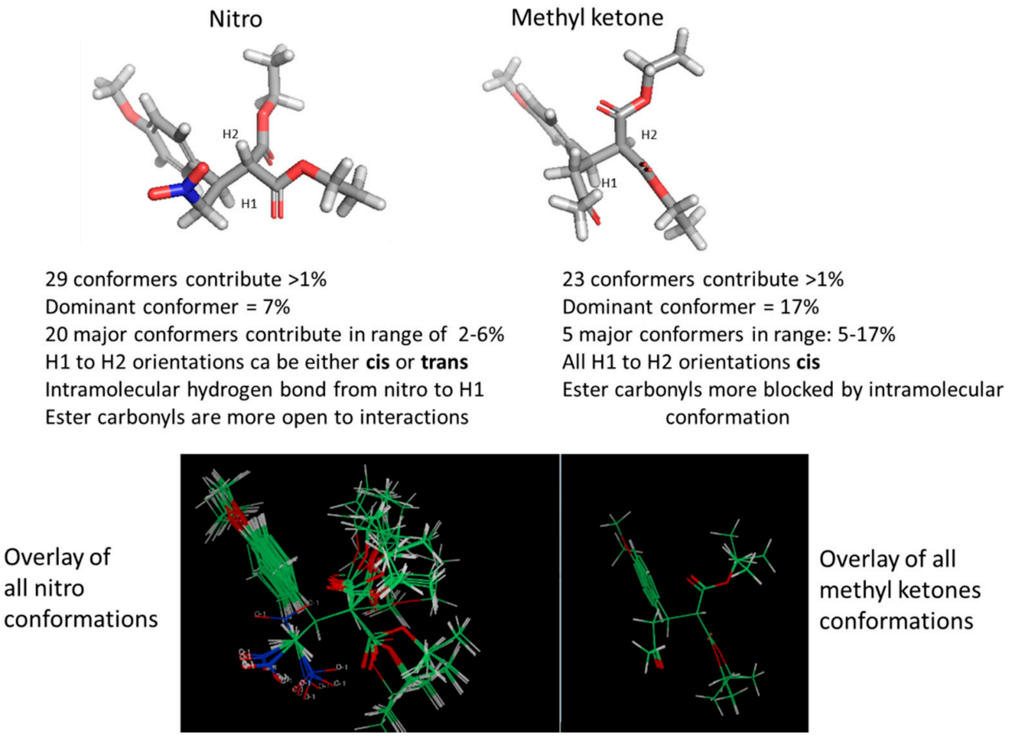

Figure S1. Minimized Conformations for Nitro and Methyl ketone Analogs from MMP.

Coordinates for lowest energy conformer of each species. All conformations for each compound available upon request.

Lowest energy nitro compound
B3LYP/6–31G** Electronic energy = –203.620229 hartrees
6 –5.48697 0.804433 0.701257
1 –5.0763 0.459098 1.65289
1 –5.25714 1.86699 0.582481
1 –6.57531 0.688973 0.733903
6 –4.92109 –0.005031 –0.453985
1 –5.32622 0.321628 –1.41396
1 –5.10999 –1.07278 –0.323911
6 –2.69203 –0.564694 0.180437
6 –1.22627 –0.191059 –0.051374
1 –1.03683 –0.260564 –1.12248
6 –1.05546 1.26601 0.387512
8 –3.48882 0.199941 –0.586279
8 –3.09146 –1.41638 0.946878
8 –1.19817 1.64275 1.53135
8 –0.730971 2.06151 –0.642229
6 –0.53763 3.46263 –0.321028
1 –1.46142 3.85005 0.11917
1 0.248745 3.54047 0.435419

6 –0.170984 4.17595 –1.60704
1 –0.014137 5.24044 –1.40753
1 0.749456 3.76597 –2.03189
1 –0.967562 4.07839 –2.3499
6 –0.244929 –1.07691 0.758556
1 –0.548879 –0.9699 1.80536
6 3.89128 0.234333 0.496072
6 3.18149 –0.063328 –0.672555
6 1.84919 –0.473795 –0.5885
6 1.19827 –0.599282 0.644036
6 1.92623 –0.28708 1.80331
6 3.25103 0.12255 1.73834
1 3.64871 0.016376 –1.64655
1 1.32244 –0.698192 –1.51098
1 1.44246 –0.361456 2.77363
1 3.81116 0.364235 2.63544
8 5.19239 0.641918 0.533455
6 5.89818 0.752076 –0.693366
1 6.90684 1.07469 –0.431847
1 5.44262 1.49742 –1.3578
1 5.9515 –0.210311 –1.21774
6 –0.377591 –2.5938 0.499353
1 0.279379 –3.14879 1.16687
1 –1.41351 –2.91019 0.624873
7 0.017223 –3.01652 –0.889572
8 0.913255 –3.84346 –1.00423
8 –0.600323 –2.51064 –1.83077

Lowest energy methyl ketone compound
B3LYP/6–31G** Electronic energy = –1151.768991hartrees
6 4.44005 –1.17952 –2.88048
1 3.7951 –2.02205 –3.1443
1 4.28571 –0.380958 –3.61193
1 5.48136 –1.50977 –2.94639
6 4.14587 –0.696321 –1.47458
1 4.7727 0.152951 –1.18751
1 4.27475 –1.48739 –0.731073
6 2.3145 0.202129 –0.272748
6 0.79866 0.394062 –0.295728
1 0.514496 0.641483 –1.32171
6 0.46189 1.60002 0.577857
8 2.75539 –0.279462 –1.44259
8 3.0175 0.416283 0.695125

8 0.018598 1.58115 1.70641
8 0.750395 2.73533 –0.08731
6 0.551597 3.96938 0.645132
1 –0.497303 4.0295 0.951065
1 1.1608 3.93762 1.55342
6 0.947138 5.11068 –0.270783
1 0.812269 6.06488 0.247834
1 1.99576 5.0266 –0.569352
1 0.330578 5.11995 –1.17404
6 0.070227 –0.938378 0.091368
1 0.451969 –1.67547 –0.62307
6 –4.20497 –0.66051 –0.658029
6 –3.69295 –0.251545 0.577658
6 –2.31968 –0.337683 0.824375
6 –1.43195 –0.830253 –0.137835
6 –1.96951 –1.23644 –1.37091
6 –3.33008 –1.15451 –1.6355
1 –4.34522 0.134565 1.35193
1 –1.93958 0.002135 1.78131
1 –1.30646 –1.63302 –2.13627
1 –3.74106 –1.47555 –2.58702
8 –5.52568 –0.622483 –1.00657
6 –6.45655 –0.13951 –0.051668
1 –7.43603 –0.198592 –0.528606
1 –6.25253 0.903352 0.223505
1 –6.46121 –0.753934 0.85793
6 0.46319 –1.46001 1.48797
1 –0.309985 –2.15768 1.84049
1 0.504483 –0.655813 2.22577
8 2.22824 –2.76746 0.500609
6 1.76771 –2.25334 1.50644
6 2.45525 –2.37562 2.84885
1 1.73883 –2.5669 3.65442
1 2.9417 –1.41668 3.06403
1 3.21094 –3.16185 2.81595

6. General Differences in Preferred Conformations for Keto and Keto Ester Analogs Shown in Figure 4e from manuscript.

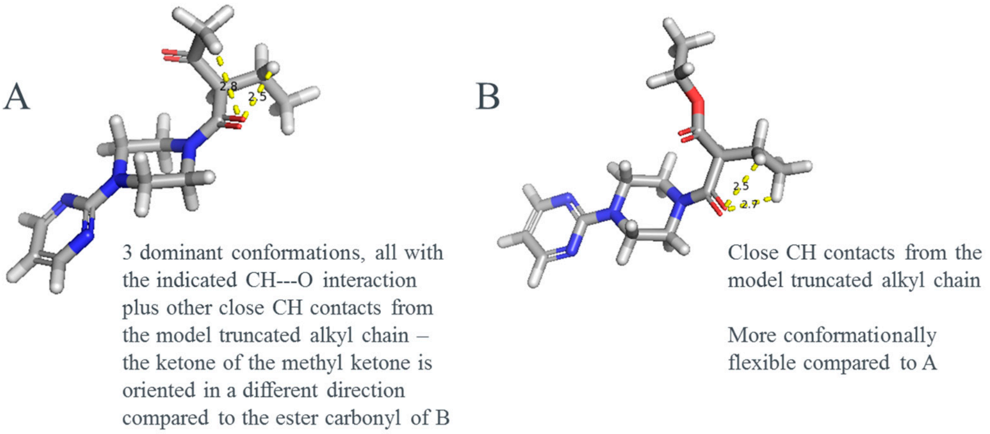

Figure S2. General Differences in Preferred Conformations for Keto and Keto Ester Analogs.

Coordinates for lowest energy conformer of each species. All conformations for each compound available upon request.

Lowest energy ketone compound A
B3LYP/6–31G** Electronic energy = –15.039736 hartrees
6 –5.26297 –0.850081 0.410776
6 –5.86033 0.1668 –0.332345
6 –4.98723 1.12362 –0.847704
7 –3.66992 1.08897 –0.657713
6 –3.19772 0.055441 0.076274
7 –3.9511 –0.924399 0.626036
7 –1.84324 –0.009194 0.272145
6 –1.21243 –1.05078 1.07485
6 –0.058282 –1.68902 0.297193
7 0.872812 –0.667809 –0.193247
6 0.233698 0.382578 –0.992048
6 –0.928608 1.01551 –0.214793
6 2.20291 –0.77912 0.116622
6 3.17723 0.273595 –0.464094
8 2.63023 –1.66754 0.852789
6 3.11716 1.51622 0.441806
6 3.83543 1.44384 1.77195
8 2.4876 2.50223 0.099116
6 4.59353 –0.320436 –0.59487
6 4.6889 –1.38822 –1.68845
1 –5.86307 –1.64271 0.856376
1 –6.93001 0.2122 –0.495499
1 –5.35982 1.95834 –1.44044
1 –1.96325 –1.79671 1.33054
1 –0.824212 –0.611786 2.00608

1 –0.461795 –2.24147 –0.56267
1 0.504692 –2.37668 0.927428
1 –0.147907 –0.057465 –1.92421
1 0.946629 1.16775 –1.23359
1 –1.48111 1.703 –0.853085
1 –0.51886 1.5806 0.635002
1 2.83839 0.60163 –1.44984
1 3.53573 2.28708 2.39534
1 4.91913 1.48369 1.61326
1 3.62274 0.493062 2.27114
1 4.88715 –0.756653 0.363126
1 5.29183 0.496072 –0.817607
1 5.71278 –1.76564 –1.77165
1 4.40199 –0.987569 –2.66741
1 4.03868 –2.23696 –1.45908

Lowest energy ester compound B
B3LYP/6–31G** Electronic energy = –1029.591846 hartrees
6 5.72826 0.162163 0.786964
6 6.24742 –0.625839 –0.239067
6 5.30449 –1.18134 –1.10257
7 3.9936 –0.985251 –0.977503
6 3.60082 –0.198666 0.0508
7 4.42589 0.38514 0.950203
7 2.25758 0.035755 0.185709
6 1.70378 0.797508 1.30049
6 0.685592 1.82033 0.790153
7 –0.326713 1.17193 –0.048243
6 0.240177 0.419989 –1.17069
6 1.26356 –0.605468 –0.667304
6 –1.6526 1.35524 0.252738
6 –2.70663 0.676432 –0.664265
8 –2.01565 2.02457 1.21446
6 –2.75106 –0.817321 –0.348132
8 –3.48742 –1.0714 0.747277
8 –2.17291 –1.67294 –0.992147
6 –4.07746 1.36112 –0.511511
6 –4.10776 2.7683 –1.11385
6 –3.58769 –2.45688 1.16573
6 –4.70631 –3.17577 0.428999
1 6.38621 0.636464 1.51439
1 7.31034 –0.798462 –0.354724
1 5.61283 –1.81506 –1.93345

1 1.20714 0.112496 2.00443
1 2.52065 1.2938 1.82205
1 0.171233 2.30606 1.61865
1 1.20463 2.58547 0.196084
1 –0.537724 –0.114256 –1.71071
1 0.736238 1.1228 –1.85546
1 0.73109 –1.38489 –0.102853
1 1.77459 –1.07583 –1.50567
1 –2.38293 0.745194 –1.7063
1 –4.32466 1.40166 0.550729
1 –4.83137 0.733229 –1.00085
1 –5.104 3.20931 –1.00965
1 –3.86246 2.755 –2.18247
1 –3.39793 3.42499 –0.60416
1 –2.62487 –2.9448 0.998008
1 –3.78524 –2.39741 2.2381
1 –4.81327 –4.19267 0.820615
1 –5.65849 –2.65457 0.563233
1 –4.48421 –3.24207 –0.638737

7. General Differences in Preferred Conformations for Urea and Thiourea Analogs Shown in Figure 4f from manuscript

Percentages are *in vacuo* Boltzmann population, where ranges cover the estimates of percent contribution determined from electronic (E) or free energies (G).

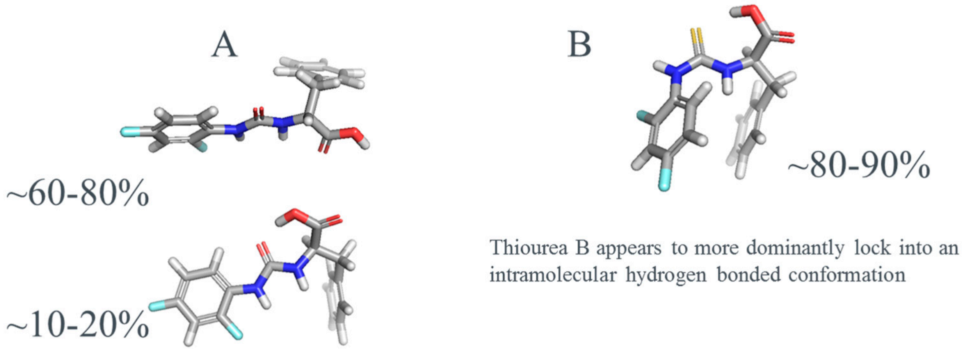

Figure S3. Preferred Conformations for Urea and Thiourea Analogs.

Coordinates for lowest energy conformer of each species. All conformations for each compound available upon request.

Lowest energy urea compound A
B3LYP/6–31G** Electronic energy = –1153.051329 hartrees
6 4.98933 0.849111 0.823067
6 3.63645 0.634741 1.01566

6 2.83144 –0.080689 0.117368
6 3.44752 –0.598797 –1.03077
6 4.81143 –0.397518 –1.2518
6 5.56111 0.31845 –0.329
7 1.47714 –0.217394 0.456271
6 0.484543 –0.83589 –0.285364
7 –0.756068 –0.77676 0.310066
6 –1.85919 –1.51723 –0.269204
6 –2.62683 –0.718725 –1.36376
8 0.665962 –1.36615 –1.37809
9 6.87944 0.511162 –0.545105
9 3.03635 1.13743 2.12817
6 –2.76441 –1.95509 0.871409
8 –3.79711 –2.70584 0.438866
8 –2.59168 –1.67728 2.03937
6 –3.3067 0.537752 –0.867251
6 –4.65714 0.520912 –0.490148
6 –5.28246 1.6732 –0.012256
6 –4.56534 2.86515 0.095676
6 –3.22175 2.89725 –0.281695
6 –2.59979 1.74392 –0.76002
1 5.57234 1.40852 1.54398
1 2.8454 –1.15144 –1.73746
1 5.29305 –0.794941 –2.1379
1 1.216 0.243988 1.31565
1 –0.851893 –0.582712 1.29941
1 –1.46768 –2.42064 –0.753601
1 –1.88355 –0.480863 –2.13
1 –3.35899 –1.39404 –1.81627
1 –4.32105 –2.93343 1.22585
1 –5.2227 –0.403087 –0.57756
1 –6.33101 1.64077 0.269951
1 –5.05123 3.76397 0.463898
1 –2.65835 3.82329 –0.209774
1 –1.55672 1.7773 –1.06072

Lowest energy thiourea compound B
B3LYP/6–31G** Electronic energy = −1475.997487 hartrees
6 –3.9684 –0.204968 0.339802
6 –2.83303 –0.632233 1.00967
6 –1.74929 –1.23303 0.353529
6 –1.84691 –1.42044 –1.03283
6 –2.96478 –0.977155 –1.73945

6 –4.00626 –0.377911 –1.04043
7 –0.652924 –1.68002 1.126
6 0.684684 –1.4666 0.925007
7 1.03851 –0.752298 –0.163949
6 2.39862 –0.283466 –0.461456
6 2.34665 1.05645 –1.21409
16 1.79458 –2.11513 2.03381
9 –5.09231 0.042242 –1.71148
9 –2.76366 –0.48917 2.34604
6 3.16462 –1.3449 –1.29375
8 3.42457 –2.49554 –0.663366
8 3.50519 –1.15082 –2.43782
6 1.58991 2.14337 –0.48011
6 0.3637 2.61375 –0.966735
6 –0.346177 3.60717 –0.287977
6 0.163941 4.14646 0.891987
6 1.3883 3.68947 1.38606
6 2.09339 2.69866 0.705465
1 –4.78964 0.25536 0.875096
1 –1.04957 –1.93705 –1.55624
1 –3.04493 –1.11456 –2.8113
1 –0.858562 –2.0897 2.02728
1 0.296808 –0.295448 –0.680909
1 2.91315 –0.164456 0.498927
1 3.38283 1.3578 –1.39576
1 1.91474 0.884537 –2.20515
1 3.08119 –2.47775 0.263882
1 –0.03309 2.21039 –1.89594
1 –1.29319 3.96061 –0.685531
1 –0.383802 4.92019 1.42155
1 1.79584 4.10823 2.30153
1 3.04773 2.35549 1.09754
